# Synthetic lethal screens reveal p300/CBP as a vulnerability for *GNAQ*-driven uveal melanomas

**DOI:** 10.1038/s41467-026-77999-8

**Published:** 2026-10-02

**Authors:** Gui-Ming Zhang, Junling Yuan, Liang Chen, Xuan-Yu Jin, Zhong-Lu Ren, Yan-Ling Zhou, Runhua Zhou, Ya-Fei Xiao, Jing-Wei Wang, Xi-An Zhang, Chongzhi Bai, Zhijie Li, Yi-Lei Li, Le Yu

**Affiliations:** 1 Department of Pharmacy and Clinical Pharmacy Center, Nanfang Hospital, Southern Medical University, Guangzhou, P. R. China; 2 Guangdong Provincial Key Laboratory of New Drug Screening, School of Pharmaceutical Sciences, Southern Medical University, Guangzhou, P. R. China; 3 Department of Neurosurgery, Nanfang Hospital, Southern Medical University, Guangzhou, Guangdong P.R. China; 4 School of Medical Information and Engineering, Guangdong Pharmaceutical University, Guangzhou, P. R. China; 5 Central Laboratory, Shanxi Province Hospital of Traditional Chinese Medicine, Taiyuan, P. R. China; 6 Guangdong Provincial Clinical Research Center for Geriatrics, Shenzhen People’s Hospital (The First Affiliated Hospital, Southern University of Science and Technology; The Second Clinical Medical College, Jinan University), Shenzhen, P. R. China

**Keywords:** Targeted therapies, Eye cancer

## Abstract

Uveal melanoma is the most common intraocular cancer in adults, driven by activating mutations in *GNAQ* or *GNA11*, which encode the G proteins Gαq and Gα11. Currently, therapeutic options for uveal melanoma, particularly metastatic uveal melanoma, remain limited. Through an isogenic CRISPR screening approach, we identify *EP300*, which encodes the protein lysine acetyltransferase p300, as a top synthetic lethal vulnerability in the context of *GNAQ* mutation. Notably, *CREBBP*, which encodes the closely related paralogue CBP, also emerges among the top-ranked candidates. Mechanistically, we find that p300 and CBP support YAP-dependent transcription downstream of oncogenic Gαq in uveal melanoma through interactions with YAP at chromatin. These interactions enhance H3K27 acetylation at the promoters and putative enhancers of YAP target genes and promote the recruitment of RNA polymerase II and CDK9. Using multiple in vivo models representing clinically relevant stages of Gαq-mutant uveal melanoma, we find that p300 inhibition, by both genetic and pharmacological approaches, profoundly impairs tumor growth and metastasis. Taken together, this study establishes p300 as a potential therapeutic target for Gαq-driven uveal melanoma, which is dependent on unrestrained YAP signaling.

## Abstract (web summary)

Uveal melanoma (UM) is often driven by mutations in GNAQ. Here, authors identify p300 as a synthetic lethal target in GNAQ-mutant UM and show that p300 inhibition impairs tumour growth and metastasis.

## Introduction

Uveal melanoma (UM) is the most common primary malignant intraocular tumor in adults^1^. While primary UM can often be effectively controlled, a significant proportion of cases (40% or more) eventually develop distant metastases, most commonly in the liver^2^. Once metastatic, UM remains a lethal disease with limited treatment options. Metastatic UM is generally unresponsive to chemotherapy or immune checkpoint inhibitors^3,4^. Although currently approved treatments such as tebentafusp (IMCgp100) and melphalan (Hepzato) have demonstrated survival benefits, there are still unmet needs due to the limitations of these drugs. Tebentafusp is only approved for patients who are HLA-A*02:01 positive, while Hepzato is limited to those with unresectable hepatic metastases involving less than 50% of the liver and no extrahepatic disease, or extrahepatic disease amenable to resection or radiation^5–7^.

UM is molecularly distinct from cutaneous melanoma (CM) and exhibits a different pattern of driver mutations. Unlike CM, where *BRAF* or *NRAS* mutations are common, UM predominantly harbors mutually exclusive somatic mutations in the G protein-coupled receptor (GPCR) signal transducers *GNAQ* and *GNA11* (encoding Gαq G proteins) in 92.5% of cases. Additional mutations occur in the GPCR *CYSLTR2* (4%) and the downstream effector *PLCβ4* (2.5%)^8^. CYSLTR2 is known to couple with Gαq to activate PLCβ4, suggesting that these genes operate within the same signaling pathway. These findings highlight the central role of constitutive activation of Gαq signaling in driving tumorigenesis in virtually all cases of UM^1,8^.

Mutations in *GNAQ*/*11* impair GTPase activity, leading to constitutive activation of G proteins in their GTP-bound state. Because Gαq proteins play essential roles in numerous physiological processes, direct targeting of Gαq remains unfeasible. Compounds such as FR900359 and YM-254890, which inhibit Gαq directly, do not distinguish between wild-type and mutant Gαq, resulting in toxicity and rendering them unsuitable for clinical use^9,10^. Consequently, targeting the downstream signaling pathways of Gαq has emerged as a more viable therapeutic strategy. Oncogenic Gαq can engage multiple downstream signaling branches, including the canonical PLCβ-PKC-MAPK cascade and YAP signaling^11^. Although oncogenic Gαq activates multiple downstream pathways, available evidence points to YAP signaling as a particularly important effector in Gαq-driven UM^12–15^. In this context, MEK inhibition has shown limited clinical benefit in metastatic UM^12^, whereas YAP has been demonstrated to mediate key tumorigenic outputs of mutant Gαq/11 in experimental UM models^13–15^. These findings support a model in which, although oncogenic Gαq activates multiple downstream pathways, YAP functions as a major downstream transcriptional effector in UM. However, YAP lacks enzymatic activity and is generally considered difficult to target directly because it does not contain a suitable small-molecule-binding pocket. Currently, no specific YAP inhibitors are in clinical use^16^. Although YAP-targeting approaches such as antisense RNA are under clinical investigation (NCT04659096), these strategies still face delivery challenges compared with conventional small-molecule therapeutics. Therefore, identifying druggable regulators of this critical downstream signaling axis may provide a more tractable therapeutic strategy for Gαq-mutant UM. Guided by this rationale, we sought to identify therapeutically actionable vulnerabilities in Gαq-mutant UM. Prior patient-level and experimental studies suggest that Gαq-driven UM is associated with transcriptional dysregulation and increased sensitivity to perturbation of transcription-associated regulators^14,17^. As key modulators of transcriptional output and chromatin state, epigenetic regulators were therefore prioritized for focused vulnerability screening.

In this work, through parallel CRISPR/Cas9 screens in an isogenic cell pair differing only in *GNAQ* status, we identify p300 as a synthetic lethal vulnerability in *GNAQ*-mutant UM. We show that p300 supports YAP-dependent transcription downstream of oncogenic Gαq and that both genetic depletion and pharmacological inhibition of p300 suppress tumor growth in vitro and in vivo, as well as metastasis in vivo. Together, these findings establish p300 as a therapeutically actionable dependency in *GNAQ*-mutant UM and support p300 inhibition as a potential strategy for targeting oncogenic Gαq-YAP signaling.

## Results

### CRISPR/Cas9 knockout screens identify *EP300* as a candidate synthetic lethal target in *GNAQ*-mutant UM

Because oncogenic Gαq signaling is associated with distinct transcriptional features in UM^14^, we asked whether Gαq(Q209P) knock-in could establish a coordinated transcriptional and chromatin state linked to exploitable vulnerabilities. To address this question, we performed RNA-seq and ATAC-seq analyzes in an isogenic pair of wild-type and Gαq(Q209P) knock-in HEK293A cells established in our previous study^17^. Differential expression analysis identified 997 significantly upregulated genes and 1,038 downregulated genes in Gαq(Q209P) knock-in cells relative to wild-type cells, indicating extensive transcriptomic rewiring (Supplementary Fig. 1a). Because transcriptional output is closely linked to chromatin accessibility, we next profiled genome-wide accessibility using ATAC-seq. Principal component analysis showed clear separation between Gαq(Q209P) knock-in and wild-type cells, indicating widespread alterations in chromatin accessibility associated with the knock-in mutation (Supplementary Fig. 1b). Integration of the RNA-seq and ATAC-seq datasets further revealed that genes upregulated in Gαq(Q209P) knock-in cells displayed increased accessibility around their transcription start sites (TSSs), whereas downregulated genes showed reduced accessibility at the corresponding regions (Supplementary Fig. 1c). Consistent with these changes, Gαq(Q209P) knock-in cells exhibited enhanced anchorage-independent growth, as evidenced by increased colony number and size in soft-agar assays (Supplementary Fig. 1d). Moreover, inducible knockdown of Gαq selectively suppressed colony formation in Gαq(Q209P) knock-in cells but had minimal effect in wild-type cells, indicating that the knock-in mutation confers a selective dependency on oncogenic Gαq signaling for sustained growth (Supplementary Fig. 1e).

To further assess the disease relevance of this Gαq(Q209P)-associated transcriptional and chromatin state, we compared the gene expression changes induced by Gαq(Q209P) knock-in in HEK293A cells with the TCGA UM cohort using a framework adapted from a prior study of Gαq-driven UM, in which Gαq-negative skin cutaneous melanoma (SKCM) samples were used as the reference control because of the limited number of Gαq-negative UM cases^14^. Using this approach, we found a highly significant overlap between genes upregulated in Gαq(Q209P) knock-in HEK293A cells and genes overexpressed in Gαq-positive human UM, defined as tumors harboring mutation or amplification in *GNAQ*, *GNA11*, or *CYSLTR2* (hypergeometric test, *p* < 3.598 × 10^−39^; see Methods). These findings support the use of the isogenic HEK293A Gαq(Q209P) system as a tractable model for identifying vulnerabilities associated with oncogenic Gαq signaling.

UM typically has a low mutation burden and remains relatively stable at the nucleotide level during metastasis^18^, suggesting that non-genetic regulatory mechanisms may contribute to disease progression. Patient-level transcriptomic analyses have also revealed distinct gene expression programs associated with Gαq-driven UM^14^. In addition, in our previous study, oncogenic Gαq mutations induced broad transcriptional changes and conferred enhanced sensitivity to transcription-associated inhibitors^17^. Together with the transcriptional and chromatin rewiring observed here in Gαq(Q209P) knock-in HEK293A cells, these findings suggested that epigenetic regulators, as key modulators of transcriptional output and chromatin state, might represent a tractable source of vulnerabilities in Gαq-driven UM. We therefore performed a CRISPR/Cas9 knockout screen using a previously characterized focused sgRNA library targeting 450 epigenetic regulators, with seven sgRNAs per gene^19^, together with 100 non-targeting control sgRNAs^20^.

**Fig. 1:**
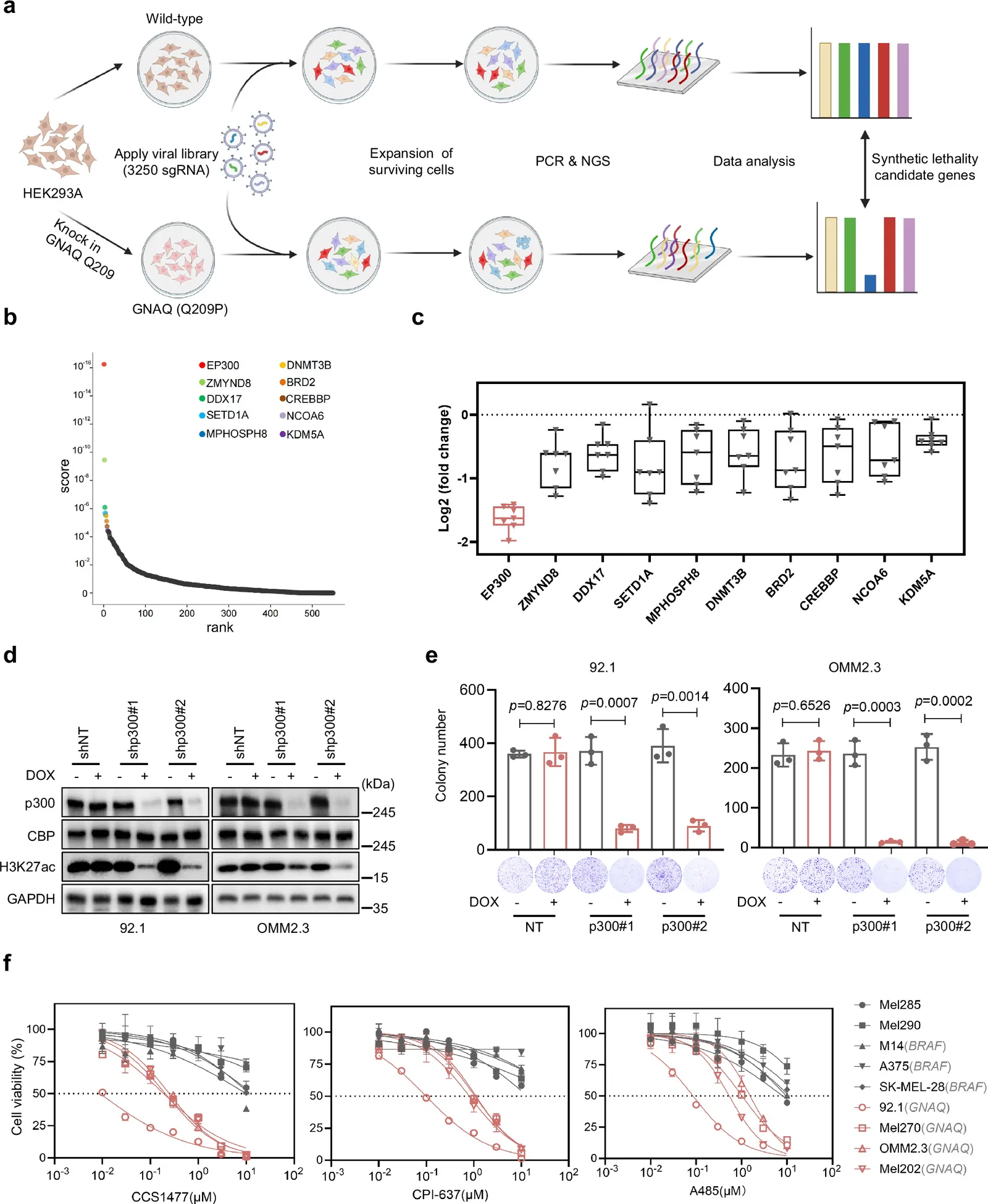
CRISPR/Cas9 synthetic lethality screens identify *EP300* as an essential gene in Gαq-mutant cells. **a** Schematic of the CRISPR/Cas9 synthetic lethality screen performed in an isogenic pair of wild-type and Gαq(Q209P)-mutant HEK293A cells. Created in BioRender. Zhang, G. (2026) https://BioRender.com/kmlqf1r. **b** Ranked gene-level depletion scores from the CRISPR/Cas9 screen in Gαq(Q209P)-mutant HEK293A cells relative to wild-type controls. Genes were ranked by robust rank aggregation (RRA) score calculated by MAGeCK; lower RRA scores indicate stronger negative selection. The top ten genes are highlighted, including *EP300* (rank 1) and *CREBBP* (rank 8). **c** Depletion of sgRNAs targeting the top ten candidate genes in Gαq(Q209P)-mutant HEK293A cells relative to wild-type controls. Each gene was targeted by seven independent sgRNAs (*n* = 7 sgRNAs per gene). Boxes span the 25th-75th percentiles, center lines indicate the medians, and whiskers extend to the minimum and maximum values. **d** Immunoblot analysis of p300 and CBP in 92.1 and OMM2.3 cells expressing doxycycline-inducible non-targeting (NT) or two independent p300-targeting shRNAs (shp300#1 and shp300#2), treated with or without doxycycline (200 ng/ml) for 72 h. **e** Colony-formation assays of 92.1 and OMM2.3 cells following inducible knockdown of p300 by doxycycline (200 ng/ml) for 14 days. Data are presented as mean ± SD (*n* = 3 biological replicates). Statistical significance was assessed using a two-sided Student’s *t* test. **f** Cell viability of Gαq-mutated UM cell lines (92.1, Mel202, Mel270, and OMM2.3), Gαq-wild-type UM cell lines (Mel285 and Mel290), and *BRAF*-mutant CM cell lines (A375, M14, and SK-MEL-28) treated with the p300/CBP bromodomain inhibitors CCS1477 and CPI-637 or the HAT inhibitor A485 for 6 days. Data are presented as mean ± SD (*n* = 3 biological replicates). Experiments in panels (**d**–**f**) were repeated three times with similar results. Exact *P-*values are indicated in the graphs. Source data are provided as a Source Data file.

We performed the CRISPR/Cas9 library screen in duplicate under conditions favoring the transduction of individual sgRNAs into each cell. Following puromycin selection, cells were cultured for 14 days, after which sgRNA abundance was quantified by next-generation sequencing (Fig. 1a). Genes that were selectively depleted in Gαq(Q209P)-mutant HEK293A cells relative to wild-type controls were identified using the MAGeCK algorithm, with lower robust rank aggregation (RRA) scores indicating stronger negative selection^21^. As expected, the non-targeting control sgRNAs were not differentially represented between Gαq(Q209P)-mutant and wild-type cells (Supplementary Fig. 2a). Among the negatively selected genes, *EP300* emerged as the top-ranked candidate, with sgRNAs targeting *EP300* showing marked depletion. Notably, *CREBBP*, which encodes the closely related paralogue CBP, was also identified as a negatively selected hit and ranked eighth in the screen (Fig. 1b, c). Because *EP300* showed the strongest negative selection, we selected p300 for further investigation.

The *EP300* gene encodes the transcriptional coactivator p300, a close paralogue of CBP encoded by *CREBBP*; both proteins possess intrinsic histone acetyltransferase activity^22^. To validate the requirement of p300 in Gαq-mutant UM cells, we generated stable 92.1 and OMM2.3 cell lines expressing doxycycline-inducible non-targeting shRNA or two independent p300-targeting shRNAs. Doxycycline treatment efficiently reduced p300 expression without affecting CBP levels (Fig. 1d), and p300 knockdown markedly suppressed colony formation in both cell lines (Fig. 1e). Because *CREBBP* also scored among the top negatively selected genes in the screen, we next assessed whether CBP similarly contributes to the growth of Gαq-mutant UM cells. In parallel inducible knockdown experiments, depletion of either p300 or CBP suppressed colony formation in both OMM2.3 and 92.1 cells (Supplementary Fig. 3a,b).

We next examined the sensitivity of Gαq-mutant UM cell lines (92.1, Mel202, Mel270, and OMM2.3) to pharmacological inhibition of p300/CBP, in comparison with Gαq wild-type UM cell lines (Mel285 and Mel290) and BRAF-mutant CM cell lines (A375, M14, and SK-MEL-28). CCS1477 and CPI-637 target the bromodomain, while A485 targets the HAT domain. Because of the high sequence similarity between p300 and CBP, specific small-molecule inhibitors for either protein alone are not currently available. Gαq-mutant UM cell lines displayed marked dose-dependent sensitivity to all three inhibitors, with half-maximal inhibitory concentration (IC50) values approximately at or below 1 μM. In contrast, Gαq-wild-type UM cell lines and BRAF-mutant CM cell lines showed minimal sensitivity, with IC50 values around or above 10 μM (Fig. 1f). Similarly, Gαq(Q209P)-mutant HEK293A cells were more sensitive to these inhibitors than their wild-type counterparts (Supplementary Fig. 2b).

To further assess the functional relationship between p300 and CBP in this setting, we compared the effects of individual and combined depletion of the two proteins. In both OMM2.3 and 92.1 cells, co-depletion of p300 and CBP did not further reduce colony formation beyond that observed with depletion of either protein alone (Supplementary Fig. 3c,d). Collectively, these data identify *EP300* as the top synthetic lethal candidate from the screen and support a functional requirement for p300/CBP in the growth of Gαq-driven UM cells.

### p300 supports YAP-dependent transcription downstream of oncogenic Gαq in UM

To gain mechanistic insight into the synthetic lethality between *EP300* and oncogenic *GNAQ*, we first examined the transcriptional changes induced by p300 depletion in 92.1 cells, a *GNAQ*-mutant UM cell line, by RNA sequencing (RNA-seq). KEGG enrichment analysis of the downregulated genes identified the Hippo pathway as the most significantly enriched pathway (Supplementary Fig. 4a,b). YAP is a major transcriptional coactivator downstream of Hippo signaling, and previous studies in UM have established YAP as a key transcriptional effector downstream of oncogenic Gαq^13–15^. We therefore investigated whether p300 contributes to YAP-dependent transcription in *GNAQ*-mutant UM cells.

**Fig. 2:**
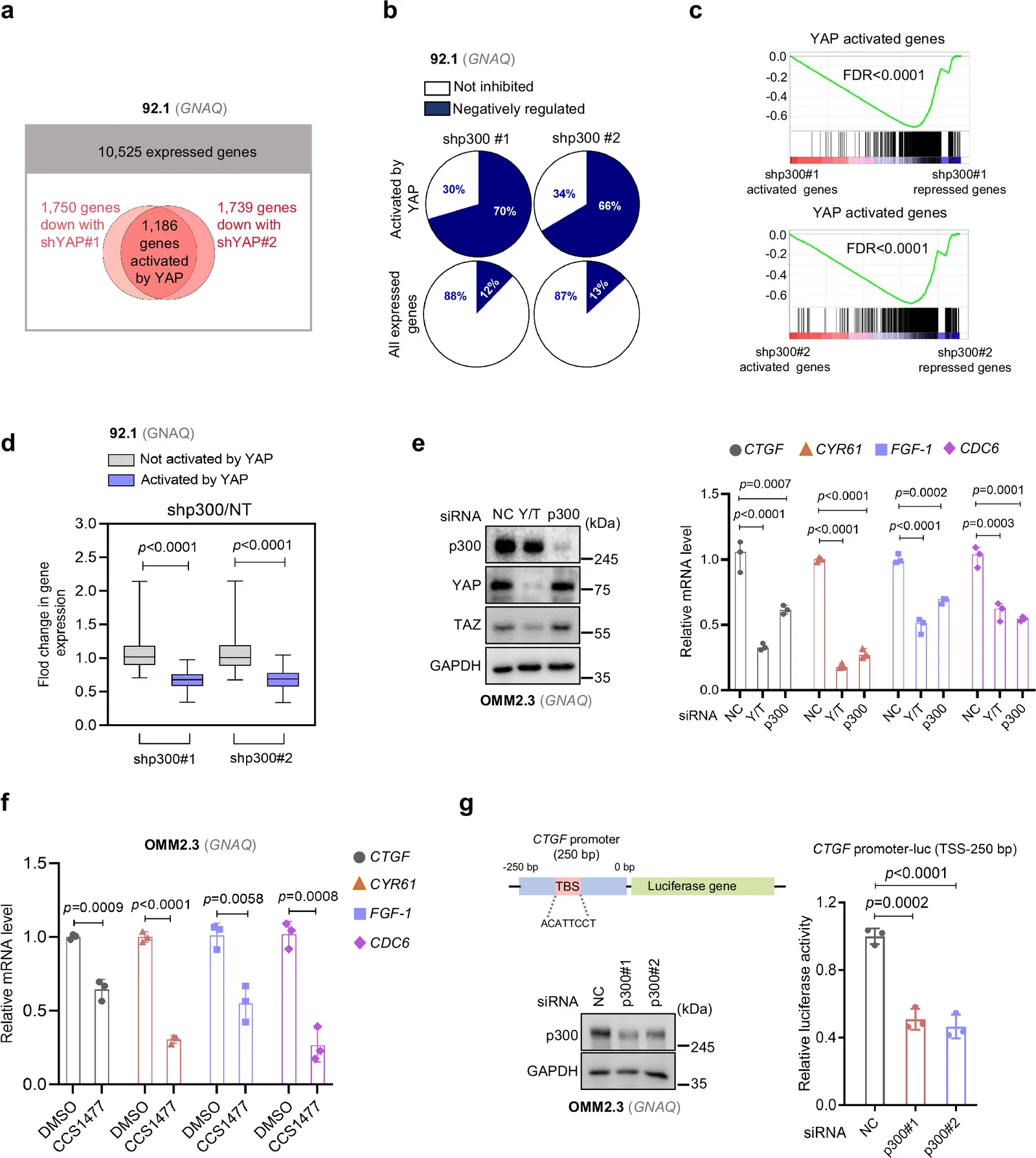
YAP transcriptional output is sensitive to p300 inhibition. **a** YAP-activated genes were defined as genes significantly downregulated by both YAP shRNA#1 and shRNA#2 in 92.1 cells based on RNA-seq analysis (*n* = 3 biological replicates; adjusted *p* < 0.05 and fold-change < 0.75). **b** Proportions of YAP-activated genes (*n *= 1186 genes) and all expressed genes (*n* = 10,525 genes) that were downregulated by p300 shRNA#1 or p300 shRNA#2 in 92.1 cells. **c** Gene Set Enrichment Analysis (GSEA) showing suppression of the YAP-activated gene set upon p300 knockdown in 92.1 cells. **d** Fold change in expression of non-YAP-activated genes (*n* = 9339 genes) and YAP-activated genes (*n* = 1186 genes) upon p300 knockdown in 92.1 cells, relative to cells transfected with NT shRNA. Boxes indicate the 25th to 75th percentiles, center lines indicate the medians, and whiskers indicate the 2.5th to 97.5th percentiles. Statistical significance was assessed using a two-sided Mann–Whitney U test. **e** RT-qPCR analysis of representative YAP target genes in OMM2.3 cells after transfection with the indicated siRNAs. Knockdown efficiency was confirmed by immunoblotting. Data are presented as mean ± SD (*n* = 3 biological replicates). Statistical significance was assessed by one-way ANOVA followed by Tukey’s multiple comparisons test. **f** Inhibition of p300 by CCS1477 suppresses mRNA expression of representative YAP target genes in OMM2.3 cells. Data are presented as mean ± SD (*n* = 3 biological replicates). Statistical significance was assessed using a two-sided Student’s *t* test. **g** *CTGF* luciferase reporter assay in OMM2.3 cells transfected with the indicated siRNAs. The reporter contains the *CTGF* promoter region (− 250 to 0 bp) with the TEAD-binding site. Knockdown efficiency was confirmed by immunoblotting. Luciferase activity was measured 48 h after transfection. TBS, TEAD binding site. Data are presented as mean ± SD (*n* = 3 biological replicates). Statistical significance was assessed using one-way ANOVA followed by Tukey’s multiple comparisons test. Experiments in panels (**e**–**g**) were repeated three times with similar results. Exact *P-*values are indicated in the graphs; values below 0.0001 are reported as *P* < 0.0001. Source data are provided as a Source Data file.

To establish a framework for assessing this possibility, we first defined the YAP-activated transcriptional program in 92.1 cells by RNA-seq following YAP knockdown. Efficient YAP depletion by two independent shRNAs was confirmed by immunoblotting (Supplementary Fig. 4c). Genes significantly downregulated by both YAP shRNAs were defined as YAP-activated genes (*n* = 1186) (Fig. 2a). This gene set was then used to assess the effect of p300 depletion on YAP-dependent transcriptional output. We compared the transcriptomes of control and p300-depleted 92.1 cells. A substantial proportion of YAP-activated genes was downregulated upon p300 knockdown, with 70% (833 out of 1186) and 66% (787 out of 1186) reduced in the p300 shRNA#1 and p300 shRNA#2 groups, respectively. By contrast, the corresponding fractions among all expressed genes were much smaller, at 12% (1294 out of 10,525) and 13% (1399 out of 10,525) (Fig. 2b). Gene Set Enrichment Analysis (GSEA) further confirmed that most YAP-activated genes were suppressed upon p300 knockdown (Fig. 2c). Consistently, p300 depletion reduced the transcript abundance of YAP-activated genes more strongly than that of the remaining expressed genes (Fig. 2d). Together, these analyses indicate that p300 preferentially supports the YAP-activated transcriptional program.

To validate the RNA-seq findings at the level of individual genes, we examined the expression of representative YAP target genes in OMM2.3 cells. siRNA-mediated p300 knockdown reduced the mRNA levels of representative YAP target genes, including *CTGF*, *CYR61*, *FGF-1* and *CDC6* (Fig. 2e). Similar results were observed following treatment with CCS1477 (Fig. 2f). We next focused on *CTGF*, a representative YAP target gene, to further examine the effect of p300 depletion on YAP target gene regulation. In OMM2.3 cells, p300 knockdown significantly reduced *CTGF* promoter-luciferase reporter activity (Fig. 2g). Because a transiently transfected promoter-luciferase reporter does not fully recapitulate the chromatin organization of the endogenous *CTGF* locus, we further examined H3K27ac enrichment at the endogenous *CTGF* promoter in the same cellular context. H3K27ac ChIP–qPCR at the endogenous *CTGF* promoter region spanning the TEAD-binding site showed that p300 depletion reduced local H3K27ac enrichment (Supplementary Fig. 4d). Together, these results indicate that p300 supports *CTGF* promoter-driven reporter activity and is also required for H3K27ac enrichment at the endogenous *CTGF* promoter in OMM2.3 cells.

Given the close paralogy between p300 and CBP, we also examined whether CBP depletion affects the same YAP-associated transcriptional program. In 92.1 cells, GSEA showed that most YAP-activated genes were suppressed upon CBP knockdown (Supplementary Fig. 5a). Consistently, YAP-activated genes were more strongly reduced than the remaining expressed genes upon CBP knockdown (Supplementary Fig. 5b). These data indicate that CBP depletion largely phenocopies p300 depletion with respect to suppression of YAP-activated genes. Together, these results indicate that p300 is required to sustain YAP-dependent transcriptional output in *GNAQ*-mutant UM cells.

### p300 interacts with YAP and TAZ

**Fig. 3:**
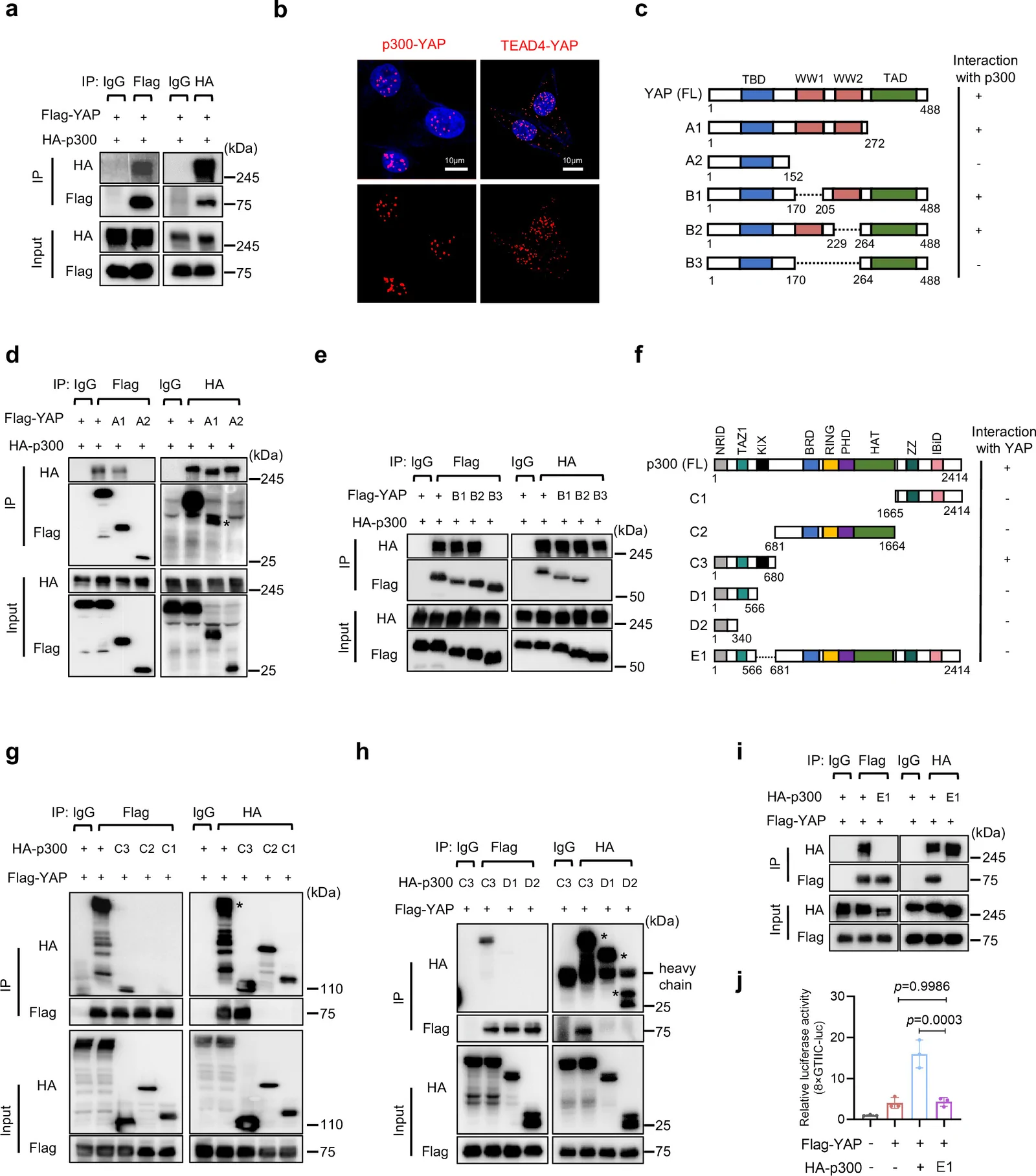
p300 interacts with YAP and enhances YAP-dependent transcription. **a** Reciprocal co-immunoprecipitation (co-IP) assays showing the interaction between p300 and YAP. HEK293T cells were transfected with the indicated constructs. YAP or p300 was immunoprecipitated with anti-Flag or anti-HA antibodies, respectively, and co-immunoprecipitated proteins were detected by immunoblotting with the indicated antibodies. **b** Endogenous interaction between p300 and YAP in the nucleus of OMM2.3 cells, as assessed by proximity ligation assay (PLA). Red dots indicate PLA signals. Nuclei were counterstained with DAPI (blue). Endogenous TEAD4-YAP interaction was used as a positive control. **c** Schematic diagram of Flag-tagged YAP constructs used for mapping the p300-binding region. **d** Amino acids 153-272 of YAP are required for interaction with p300. **e** The WW domains of YAP mediate its interaction with p300. **f** Schematic diagram of HA-tagged p300 constructs used for mapping the YAP-binding region. **g** The N-terminal region of p300 interacts with YAP. **h** Amino acids 567-680 of p300 are required for interaction with YAP. **i** The KIX domain of p300 mediates its interaction with YAP. Interactions in panels (**d**, **e** and **g**–**i**) were assessed by co-IP followed by immunoblotting, as in panel (**a**). Asterisks indicate the expected bands. **j** The KIX domain is required for p300-mediated enhancement of YAP-dependent transcription. HEK293T cells were transfected with the 8 × GTIIC-Luc reporter together with YAP alone or in combination with p300 or p300-ΔKIX. Firefly luciferase activity was normalized to Renilla luciferase activity. Data are presented as mean ± SD (*n* = 3 biological replicates). Statistical significance was assessed by one-way ANOVA followed by Tukey’s multiple comparisons test. Experiments in panels (**a**, **b**, **d**, **e**, **g**–**j**) were repeated three times with similar results. Exact *P-*values are indicated in the graphs. Source data are provided as a Source Data file.

Various nuclear partners of YAP, including transcription factors and epigenetic regulators, have been shown to modulate YAP transcriptional activity^16,23^. Although an association between YAP and p300 has been reported^24^, whether this interaction is direct and how it contributes to YAP-dependent transcription remain incompletely defined. To characterize the interaction between p300 and YAP, we first performed reciprocal co-immunoprecipitation (co-IP) assays in HEK293T cells. HA-tagged p300 co-immunoprecipitated with Flag-tagged YAP, confirming the association between ectopically expressed p300 and YAP (Fig. 3a). In addition, proximity ligation assay (PLA) demonstrated an endogenous interaction between p300 and YAP in the nucleus of OMM2.3 cells (Fig. 3b). Together, these results establish that p300 interacts with YAP under both overexpression and endogenous conditions.

To define the YAP region responsible for p300 binding, we generated a series of YAP deletion mutants (Fig. 3c). Progressive carboxy-terminal truncation analyses identified amino acids 153-272 of YAP as the minimal region required for interaction with p300 (Fig. 3d). This region contains the WW1 and WW2 domains. Deletion of either WW1 or WW2 alone had a modest effect on p300 binding, whereas simultaneous deletion of both WW domains markedly impaired the interaction (Fig. 3e), indicating that the WW domains cooperatively mediate the association between YAP and p300. We next sought to define the YAP-binding region within p300. A series of p300 truncation mutants was generated (Fig. 3f) and co-expressed with YAP in HEK293T cells. Co-IP analyses showed that the N-terminal region of p300 (1-680 aa) interacted with YAP (Fig. 3g). Further truncation mapping localized the minimal YAP-binding region to amino acids 567-680, a segment encompassing the KIX domain (Fig. 3h). Consistent with this result, deletion of the KIX domain abolished the interaction between p300 and YAP (Fig. 3i), indicating that the KIX domain is required for the association with YAP. Given the high sequence similarity between the KIX domains of p300 and CBP, we next asked whether CBP also interacts with YAP through its KIX domain. Co-immunoprecipitation assays showed that CBP interacted with YAP, whereas deletion of the CBP KIX domain abolished this interaction (Supplementary Fig. 6a–c). These results indicate that YAP binding through the KIX domain is not unique to p300.

Given that TEAD family proteins have been implicated in enhancer-associated transcriptional regulation involving p300^25^, we next asked whether the YAP-p300 association depends on TEAD proteins. Simultaneous knockdown of TEAD1/2/3/4 did not impair the interaction between YAP and p300 in co-IP assays (Supplementary Fig. 7a), arguing against a TEAD-mediated bridging mechanism. To determine whether YAP and p300 bind directly, we purified recombinant p300 (567-680 aa) and GST-YAP (155-290 aa) proteins and examined their interaction using surface plasmon resonance (SPR). p300 bound GST-YAP in a concentration-dependent manner, whereas binding to GST alone was not detected (Supplementary Fig. 7b,c). Together, these results demonstrate that YAP directly binds to p300 in a TEAD-independent manner, and that this interaction is mediated by the WW domains of YAP and the KIX domain of p300.

We next asked whether disruption of the p300-YAP interaction affects YAP-dependent transcription. Full-length p300 or a KIX-deleted mutant of p300 (p300-ΔKIX) was co-expressed with YAP in HEK293T cells, and transcriptional activity was assessed using the 8×GTIIC-Luc reporter, which contains multimerized TEAD-binding motifs and is widely used to monitor YAP/TAZ-dependent transcription^26^. Full-length p300 significantly enhanced YAP-dependent reporter activity, whereas p300-ΔKIX failed to do so (Fig. 3j). These results indicate that the KIX domain is required for p300-mediated enhancement of YAP transcriptional activity. To further define the p300 domains required for YAP-dependent gene expression, we generated p300 mutants lacking the KIX domain, bromodomain (BRD), or HAT domain and co-expressed them with YAP in HEK293T cells (Supplementary Fig. 8a,b). RT-qPCR analysis showed that deletion of any of these domains markedly impaired the ability of p300 to induce the expression of the YAP target genes *CTGF* and *CYR61* (Supplementary Fig. 8c). These results indicate that the KIX-mediated p300-YAP interaction, together with the functional contributions of the BRD and HAT domains, contributes to p300-dependent regulation of YAP target gene expression.

Because YAP and TAZ are closely related transcriptional coactivators that share TEAD-dependent transcriptional outputs, we then examined whether a similar mechanism also applies to TAZ. Co-IP assays showed that TAZ also interacted with p300, whereas deletion of the TAZ WW domain or the p300 KIX domain abolished this interaction (Supplementary Fig. 9a–e). Functionally, p300 knockdown markedly suppressed TAZ-dependent reporter activity, and wild-type p300 enhanced TAZ-dependent reporter activity, whereas p300-ΔKIX failed to do so (Supplementary Fig. 9f–h). These results indicate that p300 regulates both YAP and TAZ through a domain-dependent interaction mechanism.

To gain structural insight into the p300-YAP interaction, we used AlphaFold3 to predict the structures of p300 (567-680 aa) and YAP (171-266 aa), followed by protein-protein docking analysis using HDOCK to generate a structural model of the complex (Supplementary Fig. 10a). Structural analysis of the modeled complex suggested that the interaction may be stabilized by multiple hydrogen bonds, one salt bridge, and hydrophobic contacts (Supplementary Fig. 10b). These modeling results are consistent with the biochemical evidence supporting direct binding between p300 and YAP.

In summary, our data demonstrate that p300 directly binds YAP in a TEAD-independent manner through the KIX domain of p300 and the WW domains of YAP, and that this interaction contributes to p300-mediated enhancement of YAP-dependent transcription. A similar domain-dependent mechanism also applies to TAZ.

### p300 regulates YAP-dependent transcription independently of YAP acetylation

**Fig. 4:**
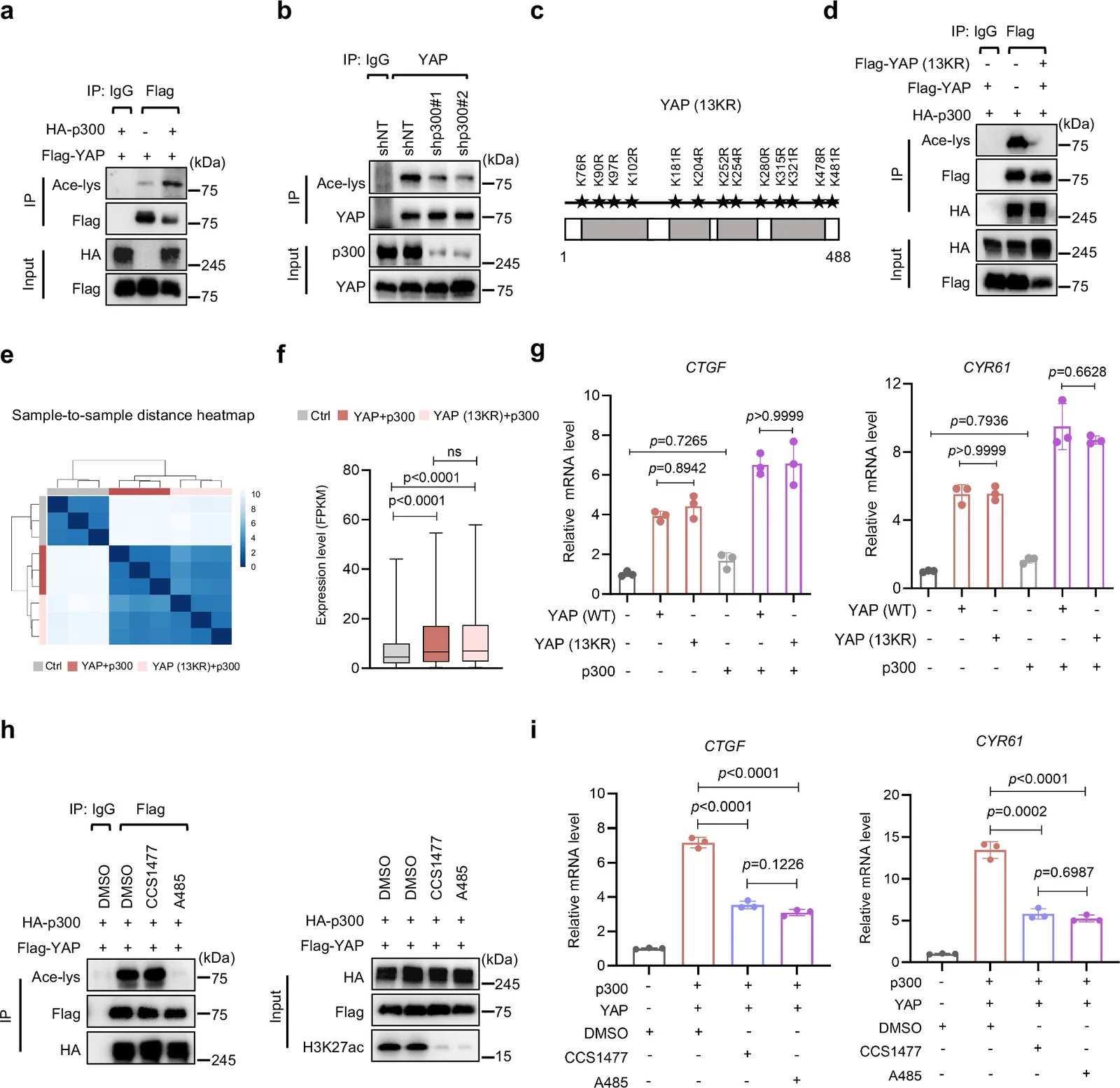
YAP acetylation is dispensable for p300-mediated YAP-dependent transcription. **a** HEK293T cells were co-transfected with Flag-tagged YAP and HA-tagged p300 constructs. YAP acetylation was assessed using an anti-acetyl-lysine antibody following immunoprecipitation with an anti-Flag antibody. **b** HEK293T cells stably expressing non-targeting (shNT) or either of two independent p300-targeting shRNAs were subjected to immunoprecipitation with an anti-YAP antibody. Endogenous YAP acetylation was assessed using an anti-acetyl-lysine antibody. **c** Schematic of the acetylation-deficient YAP (13KR) mutant, in which all 13 lysine residues were substituted with arginine. **d** YAP (13KR) showed no detectable acetylation. HEK293T cells were co-transfected with HA-tagged p300 and Flag-tagged YAP or YAP (13KR) constructs, and YAP acetylation was assessed as in panel (**a**). p300 increased acetylation of wild-type YAP but not YAP (13KR). **e** Sample-to-sample distance heatmap showing similar expression profiles between the YAP + p300 and YAP (13KR)  +  p300 groups for a predefined set of 1186 YAP-activated genes. RNA-seq was performed with 3 biological replicates. **f** Co-expression of p300 with wild-type YAP or YAP (13KR) similarly increased expression of the 1186 YAP-activated genes. RNA-seq expression values are shown as FPKM box-and-whisker plots. Boxes span the 25th-75th percentiles, center lines indicate medians, and whiskers extend to the 5th and 95th percentiles. Statistical significance was assessed using a Friedman test. **g** Wild-type YAP and YAP (13KR) similarly increased *CTGF* and *CYR61* mRNA levels with or without p300 co-expression. **h** HEK293T cells co-transfected with HA-tagged p300 and Flag-tagged YAP constructs were treated with CCS1477 (2 μM) or A485 (2 μM) for 48 h. YAP acetylation and global H3K27ac were assessed by immunoprecipitation and immunoblotting, respectively. **i** CCS1477 and A485 similarly reduced *CTGF* and *CYR61* mRNA levels in cells from panel (**h**). For (**g**, **i**), data are mean ± SD (*n*  =  3 biological replicates); statistical significance was assessed by one-way ANOVA followed by Tukey’s multiple-comparisons test. Experiments in panels (**a**, **b**, **d** and **g**–**i**) were independently repeated three times with similar results. Exact *P-*values are indicated in the graphs; values below 0.0001 are reported as *P*  <  0.0001. Source data are provided as a Source Data file.

Previous studies have shown that YAP can be acetylated by p300 under certain conditions^27^. However, whether p300 regulates YAP-dependent transcription through direct acetylation of YAP remains unclear. Consistent with previous reports, ectopic expression of p300 increased YAP acetylation in HEK293T cells (Fig. 4a), whereas stable knockdown of p300 using two independent shRNAs reduced endogenous YAP acetylation (Fig. 4b). To test whether YAP acetylation is required for YAP-dependent transcription, we generated an acetylation-deficient YAP mutant, YAP (13KR), in which all 13 lysine residues were substituted with arginine (Fig. 4c). As expected, co-expression of p300 markedly increased acetylation of wild-type YAP but not YAP (13KR) (Fig. 4d), confirming that the 13KR mutant abolishes p300-mediated acetylation of YAP.

We next examined whether loss of YAP acetylation alters p300-enhanced YAP-dependent transcriptional output. RNA-seq analysis in HEK293T cells co-expressing p300 with either wild-type YAP or YAP (13KR) showed that the two groups remained highly similar within the predefined YAP-activated gene set (*n* = 1186), as illustrated by sample-to-sample distance analysis (Fig. 4e). Consistently, co-expression of p300 with either wild-type YAP or YAP (13KR) increased expression of the same YAP-activated gene set to a comparable extent (Fig. 4f). In agreement with these transcriptome-level analyses, ectopic expression of either wild-type YAP or YAP (13KR) similarly increased mRNA levels of the canonical YAP target genes *CTGF* and *CYR61*, and co-expression of p300 further enhanced their expression to a comparable extent (Fig. 4g). Together, these results indicate that loss of YAP acetylation does not substantially impair p300-enhanced YAP-dependent transcriptional output.

We then asked whether pharmacological inhibition of p300 suppresses YAP-dependent transcription by blocking YAP acetylation. The p300 HAT domain inhibitor A485 abolished YAP acetylation, whereas the p300 bromodomain inhibitor CCS1477 had no detectable effect on YAP acetylation (Fig. 4h). Nevertheless, both inhibitors reduced global H3K27ac levels (Fig. 4h) and similarly suppressed expression of YAP target genes (Fig. 4i). In addition, treatment with either inhibitor did not alter the intracellular localization of YAP in OMM2.3 cells (Supplementary Fig. 11). These results indicate that the transcriptional effects of p300 inhibition are better associated with reduced chromatin acetylation than with loss of YAP acetylation. Together, these results indicate that p300 regulates YAP-dependent transcription primarily through modulation of chromatin acetylation rather than through direct acetylation of YAP.

### Genome-wide association between YAP and p300

**Fig. 5:**
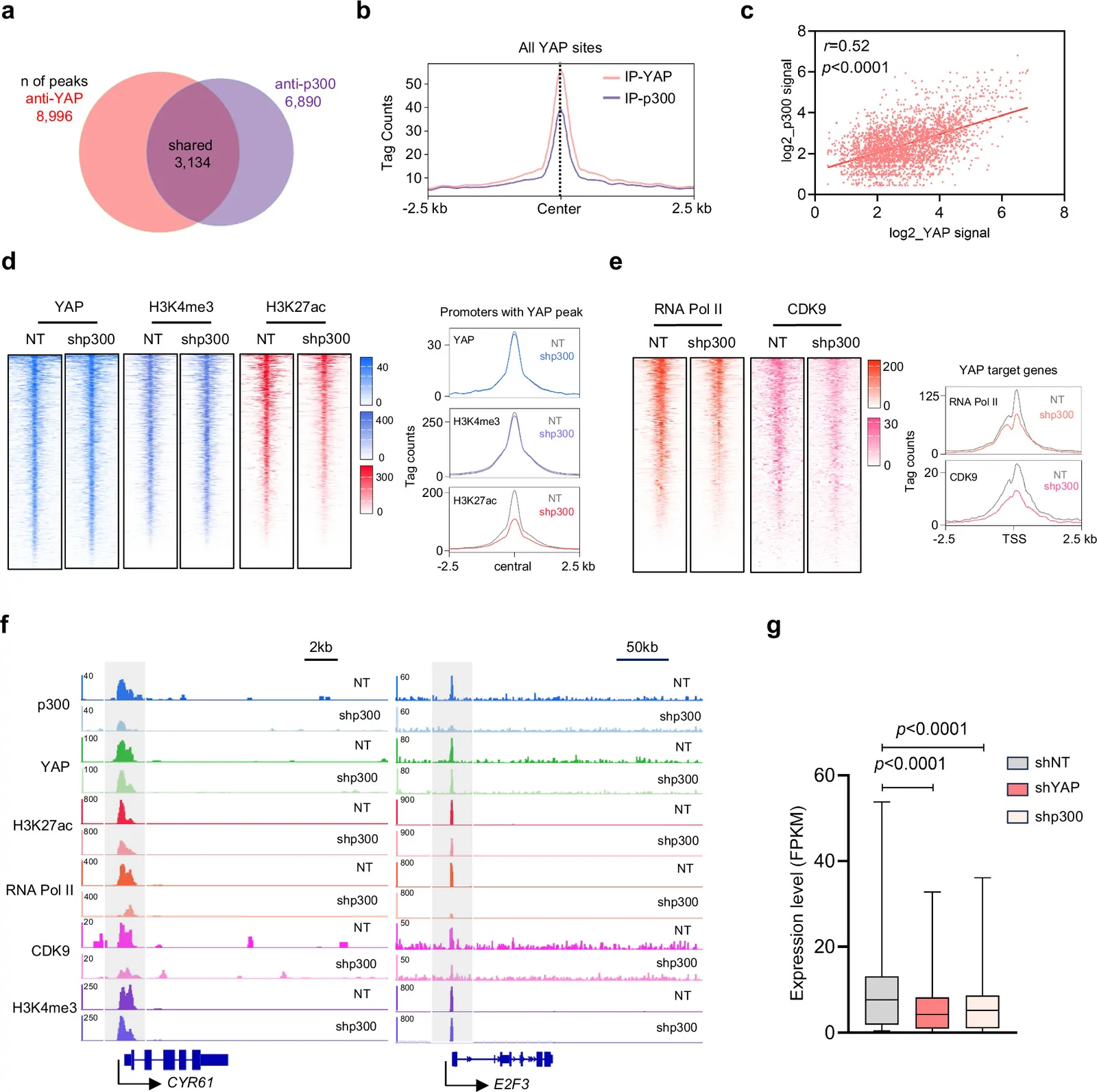
Co-occupancy of YAP and p300 at genomic loci. **a** Association of YAP and p300 ChIP-seq peaks using a 5 kb proximity window in 92.1 cells. ChIP-seq was performed with 3 biological replicates. **b** Average signal profiles of p300 ChIP-seq within a ± 2.5 kb window centered on YAP peaks (*n* = 8996 YAP peaks). **c** Positive correlation between YAP and p300 ChIP-seq signal intensities at the 3134 associated binding sites. *r* denotes the Pearson correlation coefficient. Statistical significance was assessed using a two-sided Pearson correlation test. **d** Heatmaps (left) and average line plots (right) showing YAP, H3K4me3, and H3K27ac ChIP-seq signals at promoters bound by YAP in 92.1 cells, with or without p300 depletion. **e** Heatmaps (left) and average line plots (right) showing RNA Pol II and CDK9 ChIP-seq signals at the promoters of YAP target genes in 92.1 cells, with or without p300 depletion, analyzed within a ± 2.5 kb window centered on the transcription start site (TSS). **f** Genome browser views of p300, YAP, H3K27ac, H3K4me3, RNA Pol II and CDK9 ChIP-seq profiles at representative YAP target genes, *CYR61* and *E2F3*. **g** p300 knockdown reduces the expression of YAP target genes. Gene expression levels, quantified as FPKM from RNA-seq data (*n* = 3 biological replicates), are shown as box-and-whisker plots. Boxes indicate the 25th-75th percentiles, center lines indicate the medians, and whiskers indicate the 5th-95th percentiles. Statistical significance was assessed using a Friedman test. Exact *P-*values are indicated in the graphs; values below 0.0001 are reported as *P*  <  0.0001. Source data are provided as a Source Data file.

Given the role of p300 in epigenetic regulation^22^, we investigated whether p300 influences YAP-dependent transcription through its association with YAP on chromatin. ChIP-seq analysis of endogenous YAP and p300 in 92.1 cells identified 8996 YAP-binding sites and 6890 p300-binding sites, with p300 associated with 35% of YAP-binding sites (3134/8996; Fig. 5a). The summits of p300 peaks coincided with those of YAP peaks when centered on YAP peaks (Fig. 5b). Moreover, p300 signal intensity was positively correlated with YAP signal intensity at the associated binding sites (Fig. 5c). Together, these analyses indicate a genome-wide association between YAP and p300 on chromatin.

To further investigate how p300 regulates YAP-dependent transcription on chromatin, we examined the occupancy of transcriptional machinery at the promoters of YAP target genes, defined here as genes whose promoters or proximal putative enhancers were occupied by YAP in ChIP-seq analyses. YAP occupied both promoters and proximal putative enhancers (Supplementary Fig. 12a,b). At YAP-bound promoters, p300 depletion markedly reduced H3K27ac levels, whereas H3K4me3 remained largely unchanged (Fig. 5d). Notably, YAP ChIP-seq analysis showed that YAP occupancy at these promoters was not affected by p300 depletion, indicating that YAP chromatin binding is largely independent of p300 (Fig. 5d). Similarly, p300 depletion reduced H3K27ac levels at YAP-bound putative enhancers (Supplementary Fig. 12c). This reduction in histone acetylation was accompanied by diminished recruitment of CDK9 and RNA polymerase II (RNA Pol II) at the promoters of YAP target genes (Fig. 5e), indicating reduced assembly of the transcriptional machinery. Consistently, genome browser tracks showed reduced CDK9 and RNA Pol II occupancy around the transcription start sites (TSSs) of representative YAP target genes, including *CYR61* and *E2F3*, upon p300 depletion (Fig. 5f). Integration with RNA-seq data further showed that p300 knockdown decreased expression of YAP target genes, with YAP knockdown serving as a positive control (Fig. 5g). Guided by the YAP ChIP-seq analysis, we also selected representative YAP target genes whose promoters were occupied by YAP, including *TP73*, *CYR61*, *E2F3*, *DAB2*, and *TGFB1*. ChIP-qPCR validated YAP occupancy at these promoters and showed that CBP knockdown also markedly reduced H3K27ac enrichment at these regions (Supplementary Fig. 13), suggesting that both p300 and CBP contribute to local histone acetylation at YAP-bound promoters.

Together, these results indicate that p300 promotes YAP-dependent transcription by maintaining H3K27 acetylation at YAP-bound promoters and enhancers, thereby facilitating recruitment of transcriptional machinery.

### p300 depletion selectively suppresses Gαq-mutant UM growth in vivo

**Fig. 6:**
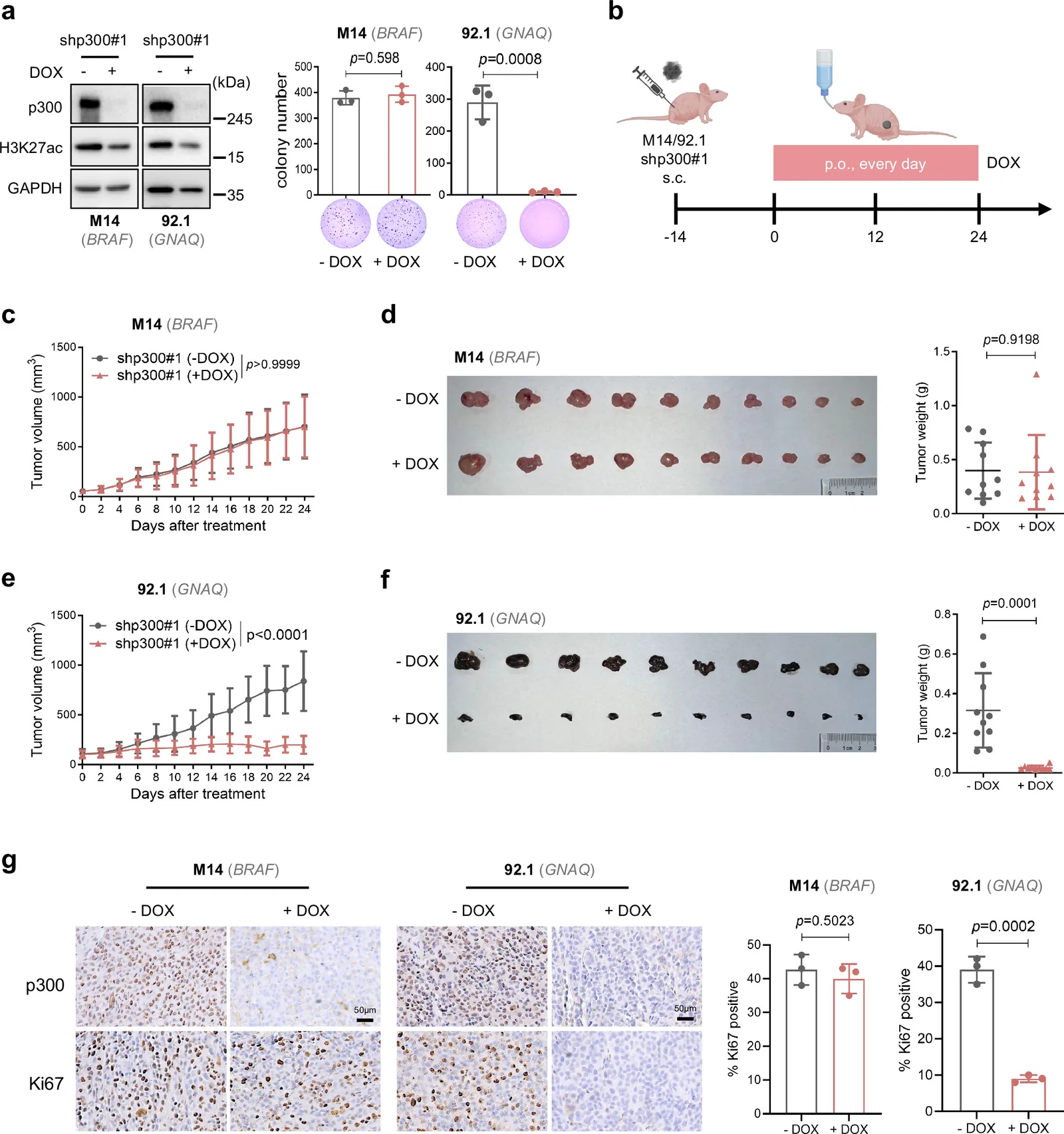
p300 depletion restricts Gαq-mutant UM growth in vitro and in vivo. **a** Effects of p300 knockdown on anchorage-independent growth of Gαq-mutant UM 92.1 cells and BRAF-mutant CM M14 cells. Cells stably expressing doxycycline-inducible p300 shRNA#1 were treated with or without doxycycline (200 ng/ml) for 72 h, and p300 knockdown was confirmed by immunoblotting. Soft-agar colony formation was assessed after 14 days of doxycycline treatment, followed by crystal violet staining and quantification. Data are mean ± SD (*n* = 3 biological replicates) from one of three independent experiments. **b** Schematic of the xenograft experiments. Created in BioRender. Zhang, G. (2026) https://BioRender.com/r0jvadb. **c** Growth curves of xenografts established by subcutaneous implantation of M14 cells stably expressing doxycycline-inducible p300 shRNA#1 into nude mice treated with or without doxycycline (0.5 mg/ml in drinking water). Data are mean ± SD (*n* = 10 mice per group). **d** Weights of M14-derived xenografts harvested on day 24. Data are mean ± SD (*n* = 10 mice per group). **e** Growth curves of xenografts established by subcutaneous implantation of 92.1 cells stably expressing doxycycline-inducible p300 shRNA#1 into nude mice treated with or without doxycycline (0.5 mg/ml in drinking water). Data are mean ± SD (*n* = 10 mice per group). **f** Weights of 92.1-derived xenografts harvested on day 24. Data are mean ± SD (*n* = 10 mice per group). **g** Representative immunohistochemical staining of p300 and Ki67 in M14- and 92.1-derived xenografts. Quantification of Ki67-positive cells is shown on the right. Data are mean ± SD (*n*  =  3 mice per group). Statistical significance was assessed using two-sided Student’s *t* tests in panels (**a**, **d**, **f** and **g**) and two-way repeated-measures ANOVA in panels (**c**, **e**). Exact *P-*values are indicated in the graphs; values below 0.0001 are reported as *P*  <  0.0001. Source data are provided as a Source Data file.

To determine whether p300 depletion selectively suppresses the growth of Gαq-mutant UM cells, we first assessed anchorage-independent growth in soft agar. Doxycycline-induced p300 knockdown preferentially inhibited colony formation of Gαq-mutant UM 92.1 cells, while having no significant effect on BRAF-mutant CM M14 cells (Fig. 6a). In contrast, doxycycline treatment did not affect anchorage-independent growth of 92.1 or M14 cells expressing inducible non-targeting shRNA (shNT) (Supplementary Fig. 14a), indicating that the growth-inhibitory effect was not due to doxycycline itself.

We next examined the impact of p300 depletion on tumor growth in vivo using xenograft models (Fig. 6b). Doxycycline-induced p300 knockdown had no detectable effect on the growth of M14 xenografts (Fig. 6c, d), but induced sustained regression of 92.1 xenografts (Fig. 6e, f). Likewise, doxycycline treatment did not affect the growth of xenografts derived from shNT-expressing 92.1 cells (Supplementary Fig. 14b–d). Notably, tumor suppression in 92.1 xenografts was accompanied by a marked reduction in the percentage of Ki67-positive cells (Fig. 6g). Together, these results indicate that p300 depletion selectively suppresses the growth of Gαq-mutant UM cells in vitro and in vivo.

### p300 inhibition suppresses metastatic progression of Gαq-mutant UM in vivo

**Fig. 7:**
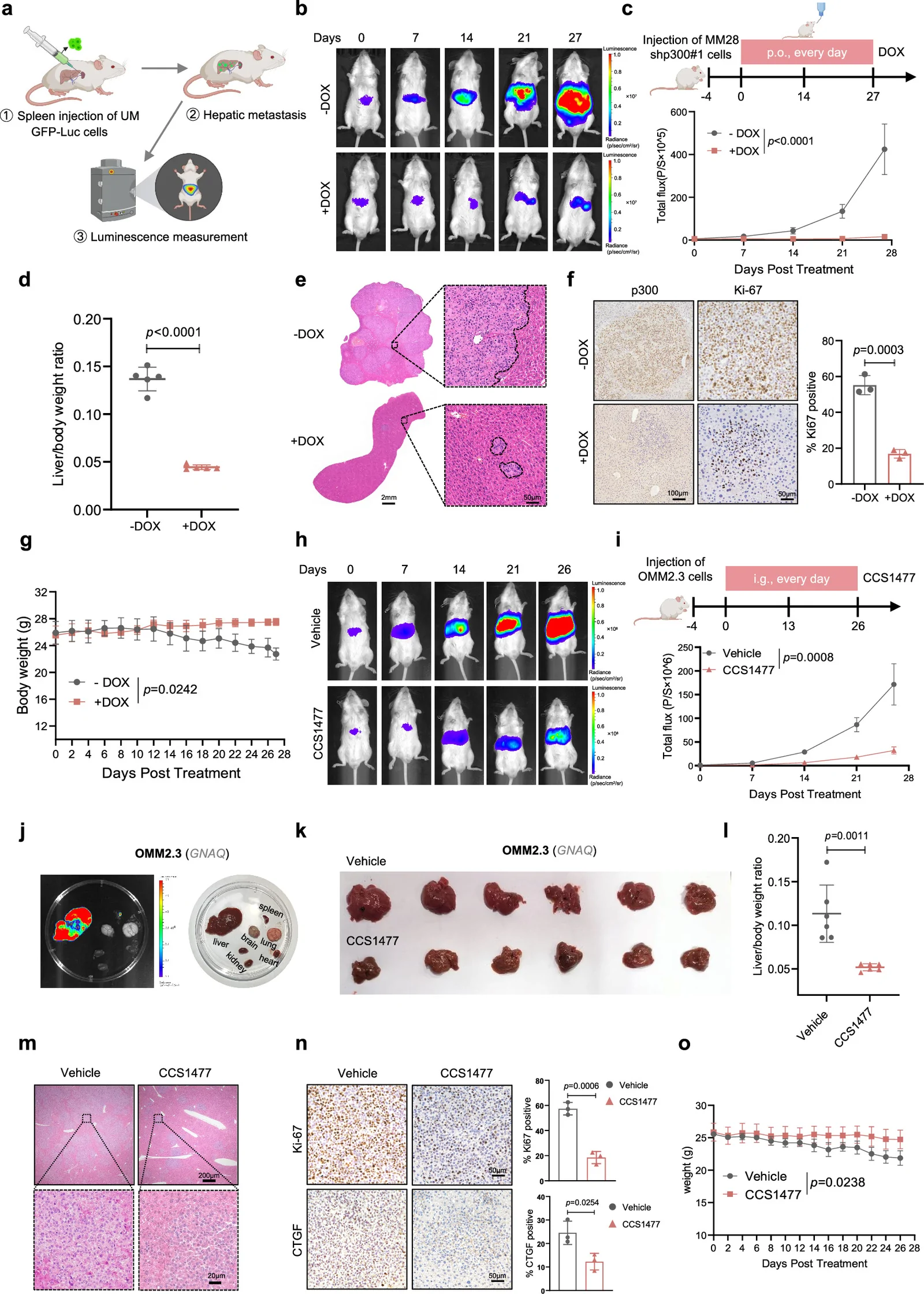
p300 depletion or inhibition suppresses UM metastasis in vivo. **a** Schematic of the intrasplenic UM-Luc metastasis model. Stable UM-Luc cells were injected into the spleens of NCG mice, and hepatic metastasis was monitored using an in vivo imaging system (IVIS). **b**, **c** Representative IVIS images (**b**) and quantification of metastatic tumor burden (**c**) in NCG mice injected with MM28-Luc cells stably expressing doxycycline-inducible p300 shRNA#1 and treated with or without doxycycline (0.5 mg/ml in drinking water). **d** Liver-to-body weight ratios in mice bearing MM28-Luc metastases. **e** Representative H&E-stained liver sections. **f** Representative immunohistochemical staining of liver sections; quantification is shown on the right. **g** Body weights of mice bearing MM28-Luc metastases. **h**, **i** Representative IVIS images (**h**) and quantification of metastatic tumor burden (**i**) in NCG mice injected with OMM2.3-Luc cells and treated with vehicle or CCS1477 (30 mg/kg). **j** Representative ex vivo images of organs collected at the experimental endpoint. **k** Livers from mice bearing OMM2.3-Luc metastases. **l** Liver-to-body weight ratios. **m** Representative H&E-stained liver sections. **n** Representative immunohistochemical staining of liver sections; quantification is shown on the right. **o** Body weights of mice bearing OMM2.3-Luc metastases. Data are mean ± SD; *n*  =  5 mice per group in panels (**c**, **d**, **g**), *n*  =  3 mice per group in panel (**f**), *n*  =  6 mice per group in panels (**i**, **l** and **o**) and *n*  =  3 mice per group in panel (**n**). Statistical significance was assessed using two-way repeated-measures ANOVA in panels (**c**, **g**, **i** and **o**) and two-sided Student’s *t* tests in panels (**d**, **f**, **l**, **n**). Exact *P-*values are indicated in the graphs; values below 0.0001 are reported as *P*  <  0.0001. BioRender elements were created by Zhang, G. (2026): panel (**a**), https://BioRender.com/kdvr27; panel (**c**) https://BioRender.com/flr9mwd; panel **i**, https://BioRender.com/moohmbk. Source data are provided as a Source Data file.

We next asked whether p300 depletion could suppress metastatic progression of Gαq-mutant UM in vivo. To address this question, we used MM28 cells, a UM cell line derived from a BAP1-deficient liver metastasis^28^. MM28 cells lack BAP1 expression (Supplementary Fig. 15a), and doxycycline-induced p300 knockdown efficiently reduced both p300 and H3K27ac levels (Supplementary Fig. 15b) and markedly impaired clonogenic growth (Supplementary Fig. 15c). We evaluated the effect of p300 depletion in an intrasplenic metastasis model using luciferase-labeled MM28 cells (Fig. 7a). Doxycycline-induced p300 knockdown significantly reduced tumor bioluminescence (Fig. 7b, c). At the experimental endpoint, this model showed strong hepatotropism (Supplementary Fig. 15d), consistent with the liver-dominant metastatic pattern of UM in patients. p300 knockdown also decreased hepatic tumor burden, as indicated by gross liver appearance (Supplementary Fig. 15e), liver-to-body weight ratio (Fig. 7d), and whole-liver H&E sections showing reduced metastatic involvement (Fig. 7e). Moreover, p300 knockdown reduced the percentage of Ki67-positive cells in liver metastatic lesions (Fig. 7f) and alleviated body weight loss (Fig. 7g).

Similar results were obtained in an additional intrasplenic metastasis model using 92.1-luciferase cells, in which doxycycline-induced p300 knockdown reduced tumor bioluminescence and hepatic metastatic burden, accompanied by a reduced percentage of Ki67-positive cells in liver metastatic lesions (Supplementary Fig. 16a–h). Consistent with these observations, an orthotopic zebrafish embryo model further showed that doxycycline-induced p300 knockdown significantly suppressed the dissemination of fluorescently labeled 92.1 cells from the injected eye to the head and trunk (Supplementary Fig. 16i).

Beyond genetic knockdown, we next evaluated the effects of pharmacological p300 inhibition using CCS1477. In an intrasplenic injection model with OMM2.3-luciferase cells in NCG mice, CCS1477 treatment significantly reduced tumor bioluminescence (Fig. 7h, i). At the experimental endpoint, this model also showed strong hepatotropism (Fig. 7j), and CCS1477 treatment alleviated hepatomegaly associated with tumor invasion (Fig. 7k, l). Histological analysis further showed a marked reduction in hepatic metastatic involvement on H&E sections (Fig. 7m). In liver metastatic lesions, CCS1477 treatment reduced the percentage of Ki67-positive cells and the percentage of CTGF-positive cells (Fig. 7n). The reduction in CTGF-positive cells indicates attenuated YAP transcriptional activity. CCS1477 treatment also mitigated body weight loss (Fig. 7o).

Together, these findings indicate that both genetic depletion and pharmacological inhibition of p300 suppress metastatic progression of Gαq-mutant UM, supporting p300 as a potential therapeutic target in this disease.

## Discussion

**Fig. 8:**
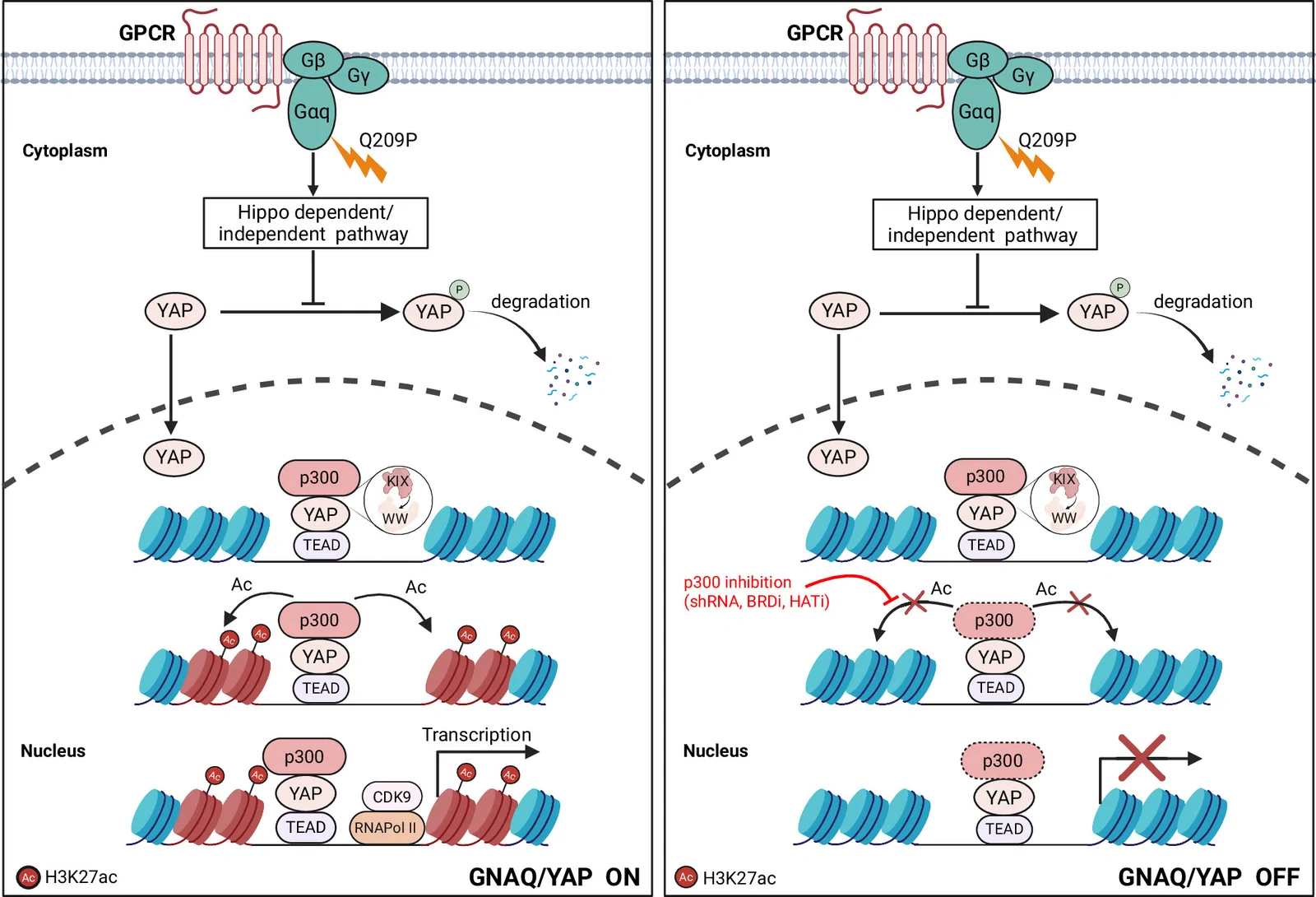
Proposed model underlying the synthetic-lethal vulnerability of *GNAQ*-mutant UM to p300 inhibition. Oncogenic Gαq promotes YAP nuclear accumulation. In the nucleus, YAP associates with TEAD to form a transcriptional complex. p300 associates with YAP through an interaction between its KIX domain and the WW domains of YAP and maintains H3K27 acetylation and the occupancy of CDK9 and RNA polymerase II at the promoters of YAP target genes, thereby sustaining YAP-dependent transcription and tumor progression (left). Genetic depletion of p300 or pharmacological inhibition of p300/CBP reduces H3K27 acetylation and the occupancy of CDK9 and RNA polymerase II at these promoters, suppressing the transcription of YAP target genes and thereby constraining *GNAQ*-mutant UM progression (right). Created in BioRender. Zhang, G. (2026) https://BioRender.com/30acp6h.

UM is a cancer characterized by stereotyped oncogenic events, notably activating Gαq mutations. This offers a promising opportunity to identify synthetic lethal targets that could benefit the majority of UM patients. Given that results from non-isogenic cell lines may be confounded by heterogeneous genetic backgrounds, we employed an isogenic CRISPR screening strategy. This approach led to the identification of p300 as a vulnerability specific to Gαq-mutant cells. Importantly, inhibition of p300, using both genetic and pharmacological methods, demonstrated potent antitumor effects in in vivo models representing clinically relevant stages of UM harboring Gαq mutations. Mechanistically, p300 sustains YAP-dependent transcription downstream of oncogenic Gαq by maintaining H3K27 acetylation and the occupancy of CDK9 and RNA polymerase II at YAP target-gene promoters, providing a mechanistic basis for the selective dependence of *GNAQ*-mutant UM on p300 (Fig. 8). Our study highlights the potential of targeting p300 in Gαq-mutant UM, particularly because direct targeting of mutant Gαq itself or its primary downstream effector YAP remains challenging.

p300 and CBP are closely related paralogous transcriptional coactivators that regulate gene expression and chromatin state, and both have been implicated in sustaining malignant transcriptional programs^29,30^. Prior studies, largely based on co-immunoprecipitation, have suggested that YAP forms complexes with p300/CBP in other biological contexts^24,31,32^. However, how p300/CBP coordinates the transcriptional activity of constitutively activated YAP downstream of oncogenic Gαq in UM remains unclear. Our data show that p300 and YAP interact in the nucleus and further demonstrate direct binding between the KIX domain of p300 and the WW1/2 domains of YAP. A similar WW-KIX-dependent interaction was also observed for TAZ, suggesting that both YAP and TAZ may be regulated by p300/CBP through a shared domain-dependent interaction. Previous studies have shown that p300/CBP can promote transcriptional activity by acetylating transcription factors, such as MYB, which increases DNA binding and transactivation^33,34^. However, our data suggest that, although p300 promotes the acetylation of YAP, this modification does not affect YAP’s transcriptional activity. Specifically, acetylation-deficient YAP and wild-type YAP exhibited similar transcriptional activity, indicating that YAP acetylation is dispensable for YAP-dependent transcription in this setting. Moreover, HAT and bromodomain inhibitors of p300/CBP suppressed the transcriptional activity of YAP despite their differing effects on YAP acetylation. Remarkably, our data suggest that the interaction between p300 and YAP at chromatin plays an important role in YAP’s transcriptional activity. Specifically, the YAP-p300 interaction enhances H3K27 acetylation at the promoters and putative enhancers of YAP target genes, thereby facilitating recruitment of transcriptional machinery. Consistent with this, CBP depletion produced effects similar to those of p300 depletion, reducing global H3K27ac, decreasing H3K27ac at YAP target gene promoters, and suppressing YAP-associated transcriptional output. These findings support the idea that both paralogues contribute to the YAP-dependent transcriptional program in Gαq-mutant UM.

The potential selectivity of targeting p300/CBP is unlikely to arise from direct discrimination between mutant and wild-type Gαq. Rather, it may reflect a context-dependent transcriptional dependency created by oncogenic Gαq signaling. In our study, this interpretation is supported by the selective depletion of *EP300* and *CREBBP* in the isogenic Gαq(Q209P) screen, the preferential sensitivity of Gαq-mutant UM cells to p300/CBP inhibition compared with Gαq-wild-type or *BRAF*-mutant melanoma cells, and the antitumor activity of p300 inhibition in vivo at tolerated doses. Conceptually, this is consistent with previous studies showing that BET inhibition can preferentially suppress YAP/TAZ-dependent transcriptional addiction through BRD4^23^. Although p300/CBP have been proposed as general regulators of RNA polymerase II-dependent transcription, genome-wide studies have instead shown that p300/CBP inhibition does not globally or indiscriminately suppress cellular transcription but rather more selectively impedes transcription of distinct oncogenic networks across different cancer types^29^. In line with these findings, we demonstrated that p300/CBP inhibition selectively disrupts the oncogenic YAP transcriptional network, which may contribute to the marked sensitivity of Gαq-mutant UM to p300/CBP inhibition. This hypersensitivity likely arises because Gαq-mutant UM is highly dependent on YAP for survival and growth^13,14,35^. In addition, this sensitivity may also reflect activation of p300/CBP HAT activity by YAP-associated complexes assembled on chromatin. It has been reported that not all p300/CBP recruitment events result in chromatin acetylation and gene expression, indicating that HAT activity at certain sites may be restrained^36,37^. Indeed, p300/CBP exists in two states: basal and active. In the basal state, the auto-inhibitory loop (AIL) occupies the substrate-binding groove and interacts with the RING domain, thereby blocking the active site of the enzyme. In the active state, the AIL is autoacetylated and disengages from the active site, allowing the RING domain to shift position and the enzyme to become active^22^. Interestingly, transcription factor dimerization has been shown to activate p300 HAT^38^. Specifically, when transcription factors dimerize, they adopt a DNA-binding-competent conformation, enabling them to engage two p300 molecules in the nucleus. This interaction increases the likelihood of AIL disengagement from its inhibitory position in cis and its capture in trans by a second p300 molecule^38^. Notably, YAP can form stable homodimers, which exhibit enhanced oncogenic behavior compared to their monomeric forms^39^. In light of these studies, together with our data showing that both CBP and p300 are required for YAP-associated transcriptional output in UM cells, we propose that YAP dimerization may promote the formation of YAP-associated transcriptional complexes that engage both p300 and CBP and facilitate HAT activation within these complexes to support YAP target gene expression in Gαq-mutant UM. The marked reduction of global H3K27ac after depletion of either paralogue, together with reduced H3K27ac at YAP target promoters, raises the possibility that YAP-associated p300/CBP complexes regulate H3K27ac as part of a dynamic acetylation-deacetylation process rather than through a simple static deposition mechanism. In this context, endogenous HDAC activity may also influence the turnover and homeostasis of H3K27ac at YAP target loci, adding another layer of complexity to YAP-dependent transcriptional regulation in Gαq-mutant UM. The precise composition, organization and functional regulation of these YAP-associated p300/CBP complexes, however, remain to be determined. This mechanism may help sustain and reinforce YAP transcriptional activity in Gαq-mutant UM, and the functional interdependency between YAP and p300/CBP may help explain the marked sensitivity of Gαq-mutant UM to p300/CBP inhibition.

Dependency on p300 and CBP appears to be context-dependent across cancers. In some tumor types, p300 dependency arises in the setting of CBP deficiency, consistent with a paralog addiction model^40,41^. In other contexts, however, tumor cells may remain dependent on both p300 and CBP^42^. Our findings in Gαq-mutant UM instead support the latter possibility. In UM cells, depletion of either p300 or CBP impaired clonogenic growth, and co-depletion was not more inhibitory than depletion of either paralogue alone. In vivo, our study focused on p300, and p300 depletion was sufficient to induce robust anti-growth and anti-metastatic effects. Analysis of the UM TCGA cohort further showed that *CREBBP* expression was not lower than *EP300*, and *CREBBP* mutations were not observed (Supplementary Fig. 17), arguing against a CBP-deficient background in this disease. Consistent with functional involvement of both paralogues, *EP300* and *CREBBP* mRNA levels positively correlated with the YAP target genes *CYR61* and *CTGF* in the UM TCGA cohort (Supplementary Fig. 18). Together, these observations suggest that dependency on p300 or CBP in Gαq-mutant UM is unlikely to be explained by loss of the other paralogue. Evidence from knockout studies in the mouse has shown that CBP and p300 are critical for the development and function of normal cells and tissues^43,44^. Therefore, inhibition of either p300 or CBP alone may be sufficient to exert anti-tumor effects and could still offer an expanded therapeutic index in Gαq-mutant UM. Because *EP300* emerged as the top-ranked hit in our screen and p300 was the paralogue validated most extensively in vivo in this study, p300-directed strategies provide a practical example for therapeutic development. While current HAT and BRD small molecule inhibitors cannot discriminate between CBP and p300, recently developed small-molecule and peptide proteolysis-targeting chimera (PROTAC) drugs demonstrate potent and selective p300 degradation^45,46^. This highlights the potential of p300-targeting PROTACs as a promising therapeutic approach. In addition, we propose that new therapeutics may be designed around the YAP-p300 interaction, particularly by targeting the p300 KIX domain. Interestingly, there has been growing interest in investigating KIX domain inhibitors, and several small molecule KIX inhibitors have been reported^47–49^.

We demonstrate that p300 inhibition suppresses tumor growth and metastasis of Gαq-mutant UM cells in xenograft mouse models and blunts the dissemination of UM cells in orthotopically xenografted zebrafish embryos. Future studies could expand on these findings by utilizing more sophisticated models of UM, such as patient-derived xenografts or organoids, to further validate the sensitivity of Gαq-mutant UM cells to p300 inhibition. We also note that the HEK293A Gαq(Q209P) knock-in system used for the discovery screen is not a lineage-faithful model of UM. Because suitable lineage-faithful isogenic models are currently not available for pooled genetic screening, we used this system as a tractable discovery platform; future studies using uveal melanocyte-derived isogenic Gαq-wild-type and Gαq-mutant models may provide a more physiologically relevant framework. This study also underscores the need for additional immunocompetent in vivo models to assess the anticancer efficacy of p300 inhibition in Gαq-mutant UM, as p300/CBP have been shown to regulate anti-tumor immune responses^50,51^. Furthermore, our findings support a functional interdependency between YAP and p300, while also supporting a role for CBP in YAP-associated transcriptional output, suggesting that YAP-dependent transcription in this context is supported by both p300 and CBP. In addition to oncogenic Gαq mutations, various mechanisms, including genetic alterations in Hippo pathway components, epigenetic dysregulation, cross-talk with other oncogenic signaling pathways, and tumor microenvironmental cues, may also contribute to YAP activation^16,52^. Therefore, future studies are warranted to explore the therapeutic potential of p300 inhibition in cancers in which YAP is aberrantly activated and functionally important.

## Methods

This study complies with all relevant biosafety, animal procedures, and ethical regulations. All animal experiments were approved by the Institutional Animal Care and Use Committee of Southern Medical University (Protocol Registry Number: SMUL2021069).

### Cell culture and reagents

Human uveal melanoma cell lines (92.1, Mel202, Mel270, OMM2.3, Mel285, and Mel290) were provided by Dr. Martine Jager (Leiden University). Human cutaneous melanoma cell lines (A375, M14, and SK-MEL-28) were obtained from Cellcook Biotech Co., Ltd. (Guangzhou, China). The human uveal melanoma cell line MM28 was obtained from MeisenCTCC (Zhejiang, China). HEK293A and HEK293T were obtained from ATCC. All cell lines were maintained in a humidified incubator at 37 ^o^C with 5% CO_2_. 92.1, Mel202, Mel270, OMM2.3, MM28, Mel285, Mel290, and M14 were cultured in RPMI 1640 (Thermo Fisher Scientific, #C11875500BT) supplemented with 10% FBS (Excell Bio, #FSP500) and 50 μg/ml penicillin/streptomycin (Invitrogen, #15140122). A375, SK-MEL-28, HEK293A, and HEK293T were cultured in DMEM (Thermo Fisher Scientific, #C11995500BT) with the same supplements. All cell lines were routinely tested for mycoplasma contamination and were negative. None of the cell lines used in this study are listed in the database of commonly misidentified cell lines. Reagents included CCS1477 (TargetMol, #T10717), CPI-637 (TargetMol, #T6811), and A485 (TargetMol, #T14073).

### Generation of Gαq (Q209P) knock-in HEK293A cells

Gαq (Q209P) knock-in HEK293A cells were generated using a CRISPR/Cas9-mediated homology-directed repair (HDR) strategy. A gene-specific single guide RNA (sgRNA) and a donor template encoding the Q209P mutation were designed using Benchling. The sgRNA was cloned into the px459 v2 plasmid (Addgene, #62988), which co-expressed Cas9 and the sgRNA. A single-stranded oligodeoxynucleotide (ssODN) donor template containing the Q209P mutation and flanking homology arms was used to mediate HDR. HEK293A cells were co-transfected with the sgRNA/Cas9 plasmid and the ssODN donor template, followed by puromycin selection (1 μg/ml) for 3 days. Surviving cells were subsequently isolated as single cells into 96-well plates by flow cytometric sorting. Individual clones were expanded, and genomic DNA was extracted using the PureLink Genomic DNA Mini Kit (Thermo Fisher Scientific, #K1820-02). Clones were screened by PCR and Sanger sequencing to identify knock-in events, and a validated heterozygous clone was used for subsequent experiments. Functional characterization of this clone, including activation of YAP signaling downstream of mutant Gαq, has been reported previously^17^. The sgRNA sequence, ssODN donor template, and PCR primers used for screening are listed below:

sgRNA: TGGTCGATGTAGGGGGCCAA;

ssODN: CCACGAGAACTTGATCATATTCACTAAGCGCTACTAGAAACATGATAGAGGTGACATTTTCAAAGCAGTGGATCCATTTTCTTCTCTCTGATCTTGGGCCCCCTACATCGACCATTCTGCAAGGTTAACA;

Primers: TTTGCCAGTAGGGCTTAACACA, GGATGACTCACTCCAACACTCC.

### CRISPR screen

A customized sgRNA library targeting 450 epigenetic regulators^19^ was obtained from Genewiz. The library comprises 3,250 sgRNAs, with 7 sgRNAs per gene and 100 non-targeting control sgRNAs^19,20^. The lentiviral vector pLentiCRISPR v2 (Addgene, #52961) was used to construct the library. Six million HEK293A and isogenic Gαq-mutant HEK293A cells (Q209P knock-in)^17^ were infected with the sgRNA library at a multiplicity of infection (MOI) of 0.3, ensuring at least 500 × coverage. After three days of puromycin selection (1 μg/ml), cells were kept in culture for two weeks. At the end of the screen, ten million cells were collected for genomic DNA extraction using the Genomic DNA Mini Kit (Invitrogen, #K1820-02). The sgRNA sequences were amplified^20^ and sequenced by Genewiz. The MAGeCK algorithm was applied to identify genes showing significant dropout in Gαq-mutant HEK293A cells compared to control HEK293A cells. Candidate hits were ranked based on robust rank aggregation (RRA) scores generated by MAGeCK^21^.

### Cell viability assay

Cells were seeded in 96-well plates, allowed to attach, and treated with the tested compounds at specified concentrations for 6 days. Viability was assessed using the MTT assay.

### Colony formation assay

Stable cells expressing doxycycline-inducible shRNAs or cells transfected with siRNAs were seeded into 12-well plates at 1500 cells per well for 92.1 cells, 1000 cells per well for OMM2.3 cells, 1500 cells per well for MM28 cells, and 600 cells per well for HEK293A cells. MM28 cells were maintained for ~ 25 days, whereas the other cell lines were maintained for ~ 2 weeks. In doxycycline-inducible experiments, medium containing 200 ng/ml doxycycline was replaced every 2 days. Colonies were fixed with 4% paraformaldehyde for 15 min and stained with 0.1% crystal violet in 5% methanol before quantification.

### Soft agar colony formation assay

Six-well plates were coated with 1.5 ml bottom agar consisting of RPMI 1640 supplemented with 10% FBS and 0.5% Difco Noble Agar. Cells were suspended in 1.5 ml top agar and seeded at the indicated densities: 5000 cells per well for 92.1 cells, 3000 cells per well for M14 cells, and 5000 cells per well for HEK293A cells. Top agar was prepared in RPMI 1640 for 92.1 and M14 cells and in DMEM for HEK293A cells, each supplemented with 10% FBS and 0.25% Difco Noble Agar. Growth medium was added on top of the agar and replaced every 2 days. In doxycycline-inducible experiments, the overlaid medium contained 200 ng/ml doxycycline. Cells were cultured for ~ 3 weeks. At the end of the assay, the overlaid medium was removed, and the wells were washed three times with PBS. Colonies were stained overnight with 0.01% crystal violet in 5% methanol and quantified.

### RNA extraction, cDNA synthesis, and quantitative real-time PCR analysis

Total RNA was extracted by the RNAsimple Total RNA kit (FOREGENE, #RE-03113). Reverse transcription was performed with the PrimeScript RT Master Mix (Takara, #RR036A). Quantitative real-time PCR (qPCR) was conducted using GoTaq qPCR Master Mix (Promega, #A6002) on a LightCycler 480 real-time PCR system (Roche). Relative mRNA levels were normalized to endogenous *GAPDH* mRNA. Human primer pairs are:

*CYR61*: AGCCTCGCATCCTATACAACC, TTCTTTCACAAGGCGGCACTC;

*CTGF*: CCAATGACAACGCCTCCTG, TGGTGCAGCCAGAAAGCTC;

*CDC6*: CGCAAAGCACTGGATGTTT, CAACCCTCTTGGGAATCAGA;

*FGF-1*: TATACGGCTCACAGACACC, TCTCTGCATGCTTCTTGGA;

*GAPDH*: CTCCTGCACCACCAACTGCT, GGGCCATCCACAGTCTTCTG.

### In situ proximity ligation assay (PLA)

In situ PLA was performed using DuoLink In Situ Reagents (Sigma, #DUO92002, #DUO92004, and #DUO92008) following the manufacturer’s instructions. The primary antibodies used were YAP (Cell Signaling Technology, #14074, 1:100), p300 (Santa Cruz Biotechnology, #sc-48343, 1:50), and TEAD4 (Abcam, #ab58310, 1:100). Images were acquired with a Zeiss LSM880 confocal microscope equipped with Airyscan; for each field, a Z-stack was acquired; images were processed using ZEN software.

### Immunohistochemistry (IHC)

Tumor xenografts were fixed in 10% aqueous buffered zinc formalin and embedded in paraffin. Sections were deparaffinized, rehydrated and subjected to heat-induced antigen retrieval using 10 mM sodium citrate buffer. Endogenous peroxidase activity was quenched with 3% H_2_O_2_ for 30 min. Sections were incubated overnight at 4 ^o^C with primary antibodies: p300 (Santa Cruz Biotechnology, #sc-48343, 1:100), Ki67 (Cell Signaling Technology, #9449, 1:500), or CTGF (Proteintech, #25474-1-AP, 1:200). Staining was visualized using Vectastain ABC kit and DAB peroxidase substrate kit (ZSGB-BIO, #ZLI-9018).

### Western blot and immunoprecipitation

Western blot was performed using standard protocols. Antibodies for Acetylated-lysine (#9441, 1:1000), CBP (#7389, 1:1000), HA tag (#3724, 1:1000), HA tag (HRP) (#14031, 1:1000), V5 tag (#13202, 1:1000), H3K27ac (#8173, 1:5000), YAP (#14074, 1:1000), TAZ (#4883, 1:1000), pan-TEAD (#13295, 1:1000) were from Cell Signaling Technology. GAPDH (#sc-25778, 1:10000), Gαq (#sc-393, 1:100) and p300 (#sc-48343, 1:100) were purchased from Santa Cruz Biotechnology. Flag tag (#FI1804, 1:1000) was purchased from Sigma-Aldrich.

For immunoprecipitations, cells were rinsed with ice-cold PBS and lysed in ice-cold lysis buffer (150 mM NaCl, 25 mM Tris-HCl pH7.4, 1 mM EDTA, 1% NP-40, 5% glycerol) supplemented with protease inhibitors (Roche, #11873580001) and phosphatase inhibitors (Thermo Fisher Scientific, #88667) on ice for 30 min and centrifuged at 12,000 rpm for 10 min. Supernatants were incubated with primary antibodies and Protein A/G magnetic beads (Invitrogen, #10004D) at 4 °C for 8 h. Immunoprecipitates were washed four times with lysis buffer, denatured in sample buffer, boiled for 5 min, resolved by SDS-PAGE, and analyzed by Western blot.

### Protein-protein docking and interaction analysis

Protein-protein docking and interaction analyses were performed as previously described^53^. Briefly, the structures of p300 (567-680 aa) and YAP (171-266 aa) were independently predicted using AlphaFold3. The predicted structures were subsequently subjected to protein-protein docking analysis using the HDOCK server to predict and rank the most probable binding mode. The resulting complex model was then analyzed using the PLIP (Protein-Ligand Interaction Profiler) platform to identify non-covalent interactions, including hydrophobic interactions, hydrogen bonds, and salt bridges. Additional interaction details were visualized and further explored using PyMOL. These analyses generated the predicted p300-YAP interaction interface and the corresponding residue-level intermolecular interactions presented in Supplementary Fig. 10.

### Immunofluorescent microscopy

Cells were fixed with 4% paraformaldehyde (15 min) and permeabilized with 0.1% Triton X-100 in PBS (5 min). After blocking, cells were incubated with anti-YAP (Cell Signaling Technology, #14074, 1:100) and Alexa Fluor 555-conjugated secondary antibodies (Thermo Fisher Scientific, #A21428, 1:500). DAPI (0.5 μg/ml) and phalloidin (Thermo Fisher Scientific, #A12379, 1:400) were used for nuclear and actin staining, respectively. Images were acquired using a Zeiss LSM880 confocal microscope equipped with Airyscan.

### Transfection

Transient transfections were performed using PolyJet DNA transfection reagent (Signagen Laboratories, #SL100688) following the manufacturer’s protocol. HEK293T cells were transfected with (pcDNA3) Flag-YAP and (pcDNA3) HA-p300 constructs.

### RNA interference

The lentiviral vector pLKO.1-TetOn-puromycin (Addgene, #24150) was used to generate shRNA constructs. The shRNA sequences targeting p300, CBP, Gαq, TEAD1/3/4 and YAP are listed in Supplementary Table 1. Lentiviruses were produced by co-transfecting HEK293T cells with the lentiviral expression plasmid, psPAX2 and pMD2.G using PolyJet DNA transfection reagent. Viral supernatants were collected 48 and 72 h after transfection, filtered through a 0.45 μm membrane, and used to infect target cells in the presence of 5 μg/mL polybrene (Sigma-Aldrich, #H9268). Infected cells were selected with puromycin (Selleck, #S7417) to establish stable cell lines, as described previously^17^.

Small interfering RNAs (siRNAs) were transfected using Lipofectamine RNAiMAX (Invitrogen, #13778030) according to the manufacturer’s protocol. Non-targeting siRNAs (Sigma-Aldrich, #SIC001) were used as negative controls. The siRNA sequences are listed in Supplementary Table 1.

### Luciferase assay

Cells were transfected with either pGL3-CTGF promoter (250 bp in length) or 8 × GTIIC-Luc reporter (Addgene, #34615), along with pRL-CMV (Renilla luciferase internal control plasmid). Following treatment, luciferase activity was measured using the Dual-Glo Luciferase Assay System (Promega, #E2920). Firefly luciferase signals were normalized with the Renilla control.

### Protein purification and binding affinity measurement

Recombinant GST-YAP (155-290 aa) was expressed in *Escherichia coli* BL21(DE3) cells, induced with 0.2 mM IPTG at 16 °C overnight, and purified using glutathione affinity chromatography. Similar procedures have been described previously^54^. Recombinant p300 (567-680 aa)-His protein was expressed in Expi293F cells and purified at 4 °C using Ni-NTA agarose affinity chromatography. Briefly, clarified culture supernatant was loaded onto a Ni-NTA column at a flow rate of 0.5 ml/min, and the flow-through was reloaded once to improve binding efficiency. The column was washed with equilibration buffer, followed by impurity-washing buffer, and the bound protein was eluted with buffer containing 250 mM imidazole. Eluted fractions were dialyzed against PBS at 4 °C to remove imidazole. Protein concentration was determined by BCA assay, and purified protein was aliquoted and stored at − 80 °C until use.

The binding affinity between GST-YAP (155-290 aa) and p300 (567-680 aa) was determined by surface plasmon resonance (SPR) on a Biacore S100 system essentially as described previously^54^. GST-YAP (155-290 aa) or GST was immobilized on a CM5 sensor chip, and serial dilutions of purified p300 (567-680 aa) protein were injected in running buffer. Sensorgrams were analyzed using Biacore S100 Evaluation Software to calculate the equilibrium dissociation constant (K_D_).

### RNA sequencing and analysis

Total RNA was extracted from cells using the RNAsimple Total RNA Kit (FOREGENE, RE-03113). Three biological replicates were prepared for each condition, and RNA samples were sent to Genewiz for sequencing. Raw reads were adapter-trimmed using Trim Galore and aligned to the human reference genome (GRCh38) using HISAT2. Gene-level read counts were generated with FeatureCounts, and FPKM values were calculated for expression visualization. Genes were considered expressed if FPKM was > 1 in at least two samples. Differential expression analysis was performed using edgeR. Upregulated differentially expressed genes (DEGs) were defined as those with an adjusted *p*-value < 0.05 and fold change > 1.33, whereas downregulated DEGs were defined as those with an adjusted *p-*value < 0.05 and fold change < 0.75. Gene set enrichment analysis (GSEA) was performed to evaluate YAP-activated gene signatures. Sample-to-sample distance analysis for YAP-activated gene sets was performed in DESeq2 using normalized count data.

To examine whether the transcriptional program induced by Gαq knock-in in HEK293A cells captures transcriptional features observed in human tumors driven by oncogenic Gαq signaling, we performed a TCGA-based comparative analysis adapted from a previously reported UM-centered framework, with UM as the tumor cohort and skin cutaneous melanoma (SKCM) as the reference cohort^14^. Gene expression profiles and copy number alteration data of TCGA-UM and TCGA-SKCM cases were retrieved from cBioPortal on March 18, 2026. The analysis included 80 UM samples and 448 SKCM samples. cBioPortal was used because it harmonizes genomic annotations across TCGA contributing centers and thereby reduces potential center-dependent inconsistencies in mutation calls. Tumors were classified as Gαq-positive (Gαq + ) if at least one Gαq-pathway gene (*GNAQ*, *GNA11*, or *CYSLTR2*) harbored either a mutation or copy-number gain. In this study, copy-number gain was defined as a GISTIC score ≥ 1. Tumors lacking mutation or amplification in all three genes were classified as Gαq-negative (Gαq − ). We then applied a two-step expression filter to identify UM-associated genes linked to oncogenic Gαq signaling. First, gene expression in Gαq + UM tumors (*n* = 77; excluding 3 Gαq − UM cases) was compared with that in Gαq − SKCM tumors (*n* = 287) using the Wilcoxon rank-sum test, and genes with *p* < 0.05 were retained, yielding 5746 candidates. Second, to minimize lineage-related differences unrelated to Gαq status, we excluded genes that were also differentially expressed between UM and the full SKCM cohort regardless of Gαq classification; specifically, only genes that were not significantly different in this comparison (Wilcoxon rank-sum *p* > 0.05) were retained. This filtering step resulted in 687 genes out of 19,433 tested genes. To determine whether the UM-derived Gαq-associated gene set significantly overlapped with the transcriptional program induced by Gαq(Q209P) in HEK293A cells, we compared these 687 genes with the 997 genes upregulated in HEK293A Gαq(Q209P) knock-in cells relative to parental HEK293A cells. The significance of overlap was assessed using a hypergeometric test with the 19,433 tested genes as the background set.

### ATAC sequencing and analysis

HEK293A cells and HEK293A Gαq (Q209P) knock-in cells were prepared in three biological replicates each. After cell lysis, transposition was performed using Tn5 transposase (Illumina, #FC-121-1030) at 37 °C for 30 min. Transposed DNA was purified using the MinElute PCR Purification Kit (Qiagen, #28004). Libraries were pre-amplified for five cycles using custom Nextera PCR primers and NEBNext Q5 Hot Start HiFi PCR Master Mix (NEB, #M0543S), and the additional number of amplification cycles was determined by qPCR using an aliquot of the pre-amplified product. Final PCR products were purified using Agencourt AMPure XP beads (Beckman Colter, #A63881) according to the manufacturer’s instructions.

Libraries were sequenced on an Illumina NovaSeq X Plus platform (PE150; Novogene Biotech, China). Raw reads were adapter-trimmed using Trim Galore and aligned to the hg38 genome using Bowtie2. Aligned reads were filtered for low-quality reads and duplicates using SAMtools. Peaks shared across biological replicates were identified using BEDtools intersect. Heatmaps and average profile plots were generated using deepTools, and principal component analysis (PCA) was performed using the R package ggplot2.

### Chromatin immunoprecipitation (ChIP), ChIP-seq and ChIP-qPCR

For ChIP-seq, stable 92.1 cells expressing doxycycline-inducible non-targeting or p300 shRNAs were treated with doxycycline (200 ng/ml) for 96 h before the assay. For ChIP-qPCR, OMM2.3 cells were transfected with siRNAs targeting p300, and stable 92.1 cells were treated with doxycycline (200 ng/ml) for 96 h to induce CBP knockdown. For each immunoprecipitation, approximately 5 × 10^6^ cells were processed using the SimpleChIP Plus Enzymatic Chromatin IP Kit (Cell Signaling Technology, #9005) following the manufacturer’s protocol. The following antibodies were used for ChIP: YAP (Abcam, #ab52771, 1:50), p300 (Sigma-Aldrich, #05-257, 1:50), H3K4me3 (Cell Signaling Technology, #9751, 1:100), H3K27ac (Cell Signaling Technology, #8173, 1:100), RNA Pol II (Cell Signaling Technology, #2629, 1:100) and CDK9 (Santa Cruz Biotechnology, #sc-13130, 1:100). Normal rabbit IgG was included as a negative control in both ChIP-seq and ChIP-qPCR experiments. Three biological replicates were prepared for each ChIP-seq condition.

For ChIP-seq, sequencing libraries were sequenced on the Illumina NovaSeq X Plus PE150 platform (Novogene Biotech, China). Raw reads were adapter-trimmed using Trim Galore and aligned to the hg38 genome using Bowtie2. Uniquely mapped reads were retained using SAMtools. Peaks were called with MACS2 and annotated using ChIPseeker. Data were visualized in IGV, and heatmaps and average profile plots were generated using deepTools. Reproducible peaks were identified by intersecting peak sets from biological replicates using BEDtools. YAP peaks were considered associated with p300 peaks when a p300 peak was located within 5 kb of the YAP peak. Promoters were defined as the 3 kb region upstream of each transcription start site (TSS). Putative enhancers were defined as non-promoter regions enriched for H3K27ac^55,56^.

For ChIP-qPCR, immunoprecipitated DNA was quantified by quantitative real-time PCR and normalized to input DNA. Primer sequences are listed in Supplementary Table 2.

### Animals

Nude mice were purchased from Guangdong Medical Laboratory Animal Center (Guangzhou, China), and NCG mice were obtained from GemPharmatech (Jiangsu, China). The mice were housed in the specific pathogen-free (SPF) animal facility at Southern Medical University. Prior to the initiation of the experiments, a one-week acclimation period was allowed for the mice to adjust to the laboratory environment. All mice were housed under controlled conditions with a 12 h light/dark cycle, a temperature range of 22–24 °C, and 50–60% humidity. Throughout the study, they had continuous free access to food and water. Only male mice were used in this study to minimize variability associated with sex and to maintain consistency across experiments. Accordingly, sex was not included as an experimental variable in the study design or analysis. The maximal tumor volume permitted by the Institutional Animal Care and Use Committee was 2000 mm^3^, and this limit was not exceeded in any of the animal experiments. Wild-type AB zebrafish were purchased from China Zebrafish Resource Center (Wuhan, China). Zebrafish embryos were maintained at 28 °C under standard laboratory conditions.

### Tumor xenograft experiments

To evaluate the effect of p300 knockdown on tumor growth, stable 92.1 cells expressing doxycycline-inducible p300 shRNA#1 (5 × 10^6^ cells), stable M14 cells expressing doxycycline-inducible p300 shRNA#1 (3 × 10^6^ cells), and stable 92.1 cells expressing doxycycline-inducible non-targeting shRNA (5 × 10^6^ cells) were subcutaneously injected into the left back flanks of 6-week-old male nude mice. Once tumors became palpable, mice were randomly assigned to either the vehicle control group or the doxycycline treatment group. Mice in the vehicle control group received drinking water supplemented with 5% sucrose, whereas mice in the treatment group received 0.5 mg/ml doxycycline in drinking water supplemented with 5% sucrose. The doxycycline-containing water was changed every 2 days. Tumor height and width were measured using a caliper, and tumor volume was calculated as width^2 ^× height × 0.5. Mice were euthanized after 24 days of doxycycline treatment. Tumor xenografts were excised, recorded and fixed for further analysis.

To assess the effect of p300 knockdown on tumor cell dissemination, stable 92.1 cells expressing doxycycline-inducible p300 shRNA#1 were fluorescently labeled with CM-DiI (Thermo Fisher Scientific, #C7000) and injected into the posterior eye region of zebrafish embryos (48 h post-fertilization). Misinjected embryos were removed, and the remaining embryos were treated with or without 10 µg/ml doxycycline and maintained at 34 °C. At 4 days post-injection (dpi), tumor cell dissemination was assessed using an Olympus MVX10 Zoom Fluorescence Macro System Microscope. Tumor cells migrating from the injection site (eye) to distal regions (head, trunk, and tail) were manually counted and defined as disseminated cells.

### Liver metastasis mouse model

Six-week-old male NCG mice were injected intrasplenically with stable MM28-Luc (1 × 10^6^) or 92.1-Luc (1 × 10^6^) cells expressing doxycycline-inducible p300 shRNA#1 or OMM2.3-Luc (1 × 10^6^) cells. Tumor implantation was monitored weekly via bioluminescence imaging using the In Vivo Imaging System (IVIS) (PerkinElmer). Before imaging, mice were intraperitoneally injected with 150 mg/kg D-luciferin potassium salt (PerkinElmer, #122799) dissolved in sterile PBS (Gibco, #10010023) 15 min prior to imaging. On day 4 post-surgery, mice were randomized into treatment groups. For the MM28-Luc and 92.1-Luc models, mice were given 0.5 mg/ml doxycycline in drinking water supplemented with 5% sucrose, while the control group received vehicle (5% sucrose in drinking water). For the OMM2.3-Luc model, mice were treated daily with 30 mg/kg CCS1477 (TargetMol, #T10717) via oral gavage, while the control group received vehicle (200 μl of 0.5% CMC-Na solution). Mice were euthanized at the indicated time points, and tumors were collected for histological and IHC analysis.

### Statistical analysis

Data are presented as the mean ± standard deviation (SD) unless otherwise specified. Statistical analyses were performed using GraphPad Prism software. Statistical significance was determined using two-sided Student’s *t* tests for comparisons between two groups and analysis of variance (ANOVA) for comparisons among multiple groups. The specific ANOVA models, post hoc tests and other statistical methods are indicated in the corresponding figure legends. *P*  <  0.05 was considered statistically significant.

### Reporting summary

Further information on research design is available in the Nature Portfolio Reporting Summary linked to this article.

## Supplementary information

Supplementary Information

Reporting Summary

Transparent Peer Review file

## Source data

Source Data

## Data Availability

The raw sequencing data generated from the CRISPR screen have been deposited in the NCBI Sequence Read Archive (SRA) database under accession number PRJNA1518397. The sequencing data generated in this study have been deposited in the NCBI SRA database under accession numbers PRJNA1175074, PRJNA1222236, PRJNA1397940, and PRJNA1397882. All other data are available within the article and its Supplementary Information. No custom code was generated for this study. Source data are provided in this paper.
