# Supplementary material for: Synthetic lethal screens reveal p300/CBP as a vulnerability for GNAQ-driven uveal melanomas
Source: Nat Commun. 2026 Oct 2;17:11303. doi: 10.1038/s41467-026-77999-8 (PMC13724638; doi:10.1038/s41467-026-77999-8)
Supplement: Supplementary file 1 [file 41467_2026_77999_MOESM1_ESM.pdf]

Supplementary information for:

Synthetic lethal screens reveal p300/CBP as a vulnerability for *GNAQ*-driven uveal melanomas

Supplementary Figures 1-18

Supplementary Tables 1 and 2

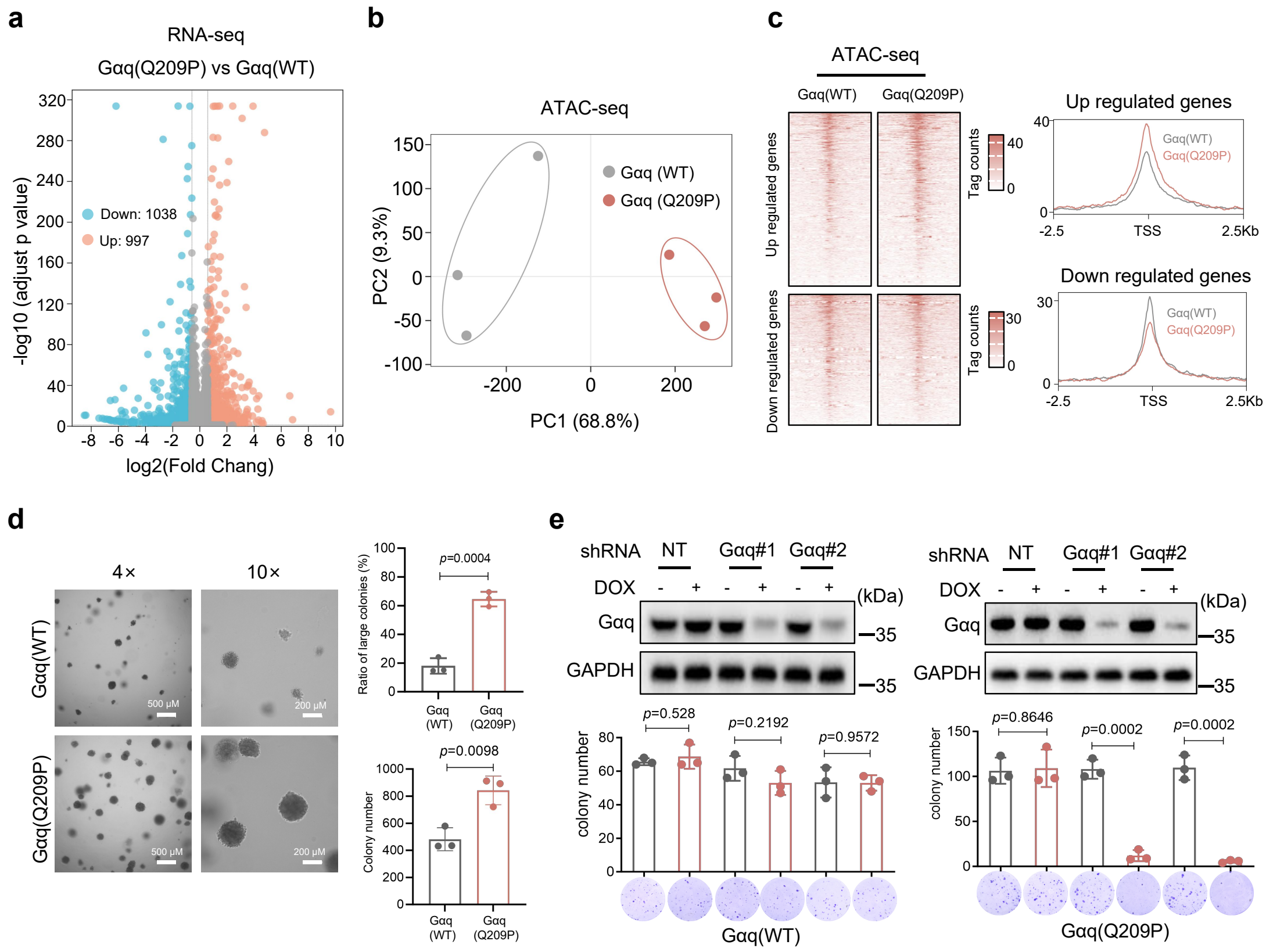

Supplementary Fig. 1: Gαq(Q209P) knock-in induces transcriptomic rewiring and global alterations in chromatin accessibility.

a, Volcano plot showing transcriptional changes in Gαq(Q209P)-mutant HEK293A cells relative to wild-type cells. A total of 997 genes were significantly upregulated and 1,038 genes were significantly downregulated, as defined in the Methods. RNA-seq was performed with 3 biological replicates.

b, Principal component analysis of ATAC-seq profiles in Gαq(Q209P)-mutant and wild-type HEK293A cells. Biological replicates cluster according to genotype, indicating global differences in chromatin accessibility. ATAC-seq was performed with 3 biological replicates.

c, Integration of RNA-seq and ATAC-seq datasets. Heatmaps (left) and average accessibility profiles (right) show chromatin accessibility around transcription start sites (TSSs; ± 2.5 kb) of genes significantly upregulated (n = 997 genes) or downregulated (n = 1,038 genes) in Gαq(Q209P)-mutant HEK293A cells. Upregulated genes show increased accessibility around their TSSs, whereas downregulated genes show reduced accessibility.

d, Soft-agar assays comparing anchorage-independent growth of Gαq(Q209P)-mutant and wild-

type HEK293A cells. Colonies with diameters >100 μm were scored as positive, and the fraction of colonies ≥ 200 μm was quantified (>1,000 colonies counted per sample). Data are presented as mean ± SD (n = 3 biological replicates) from one representative experiment out of three. Statistical significance was assessed by two-sided Student's *t*-test.

e, Immunoblot analysis of Gαq in Gαq(Q209P)-mutant HEK293A cells expressing doxycycline-inducible non-targeting (NT) or Gαq-targeting shRNAs, treated with or without doxycycline (200 ng/ml). Colony-formation assays show that inducible Gαq knockdown selectively suppresses growth of Gαq(Q209P)-mutant cells. Data are presented as mean ± SD (n = 3 biological replicates) from one representative experiment out of three. Statistical significance was determined by two-sided Student's *t*-test.

Exact *P* values are indicated in the graphs. Source data are provided as a Source Data file.

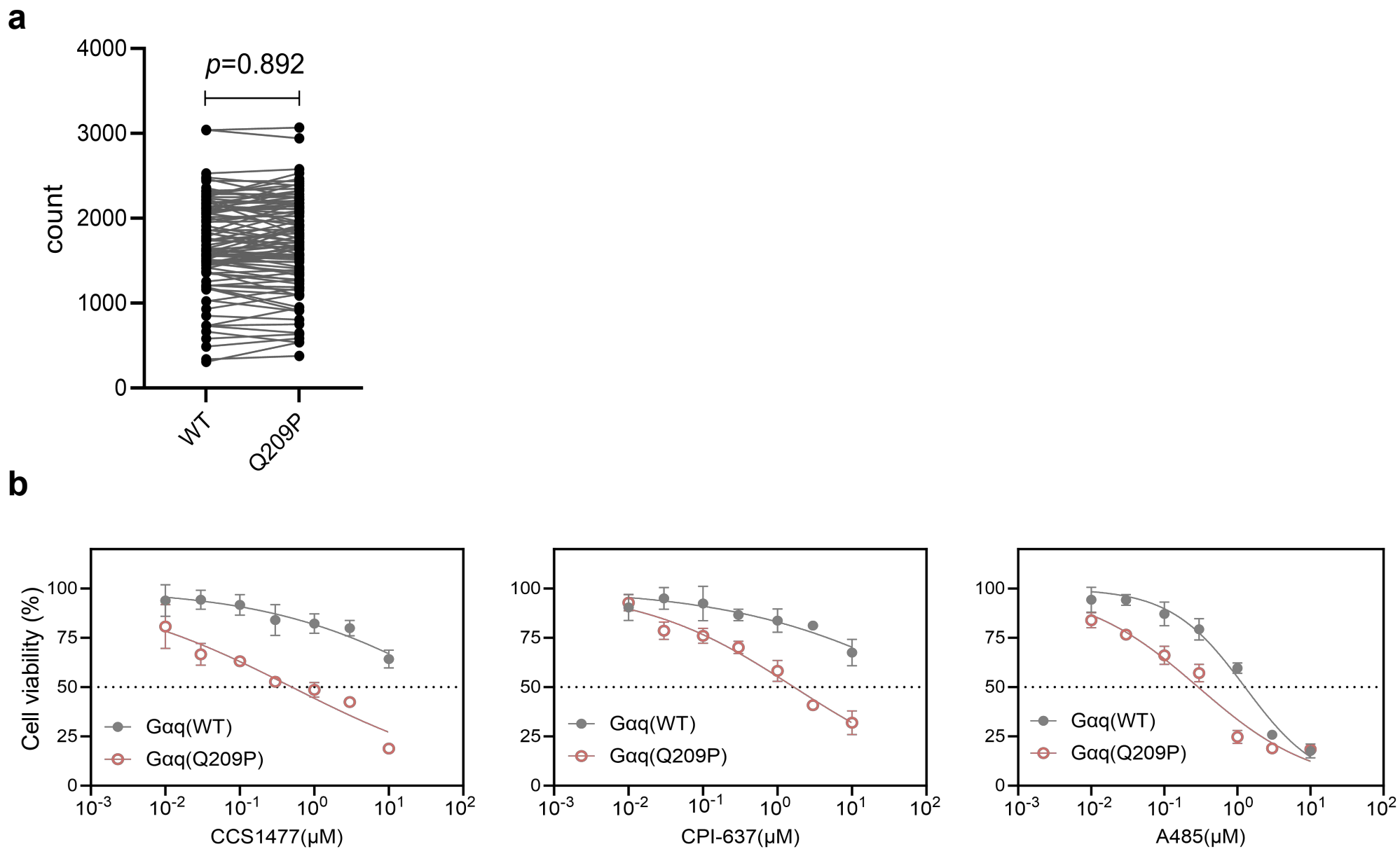

Supplementary Fig. 2: CRISPR/Cas9 synthetic lethality screens support a selective requirement for p300/CBP-associated functions in Gαq-mutant cells.

a, Abundance of non-targeting control sgRNAs (n = 100) in Gαq(Q209P)-mutant and wild-type HEK293A cells from the CRISPR/Cas9 screen. No significant difference was observed. Statistical significance was determined by a paired two-sided Student's *t*-test.

b, Cell viability of Gαq(Q209P)-mutant HEK293A cells and their wild-type parental counterparts treated with the p300/CBP bromodomain inhibitors CCS1477 and CPI-637 or the HAT inhibitor A485 for 6 days. Cell viability was measured by MTT assay. Data are presented as mean ± SD (n = 3 biological replicates) from one representative experiment out of three.

Exact *P* values are indicated in the graphs. Source data are provided as a Source Data file.

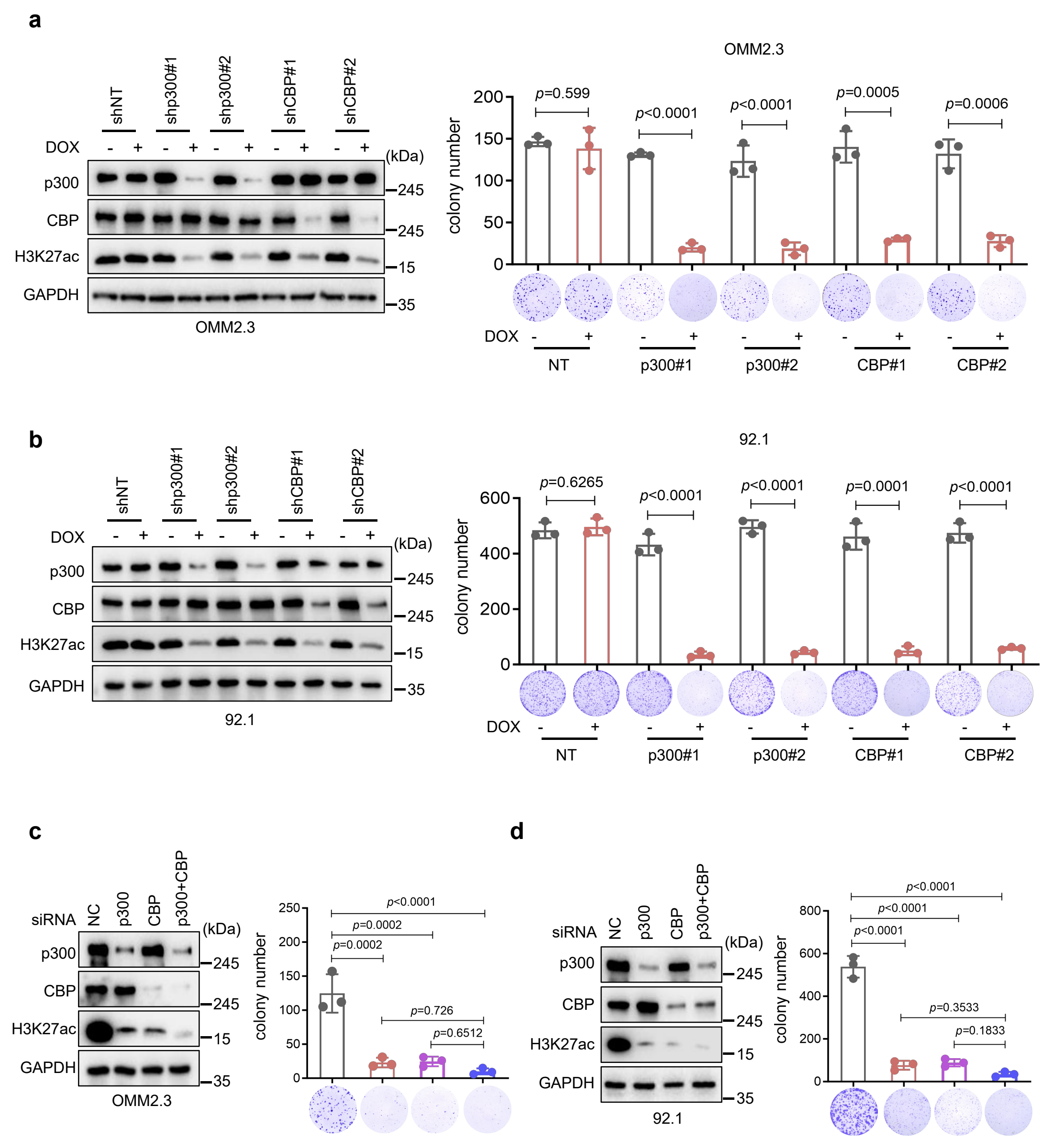

Supplementary Fig. 3: Individual depletion of p300 or CBP suppresses clonogenic growth of Gαq-**mutant UM cells.**

a and b, Immunoblot analysis and colony-formation assays in OMM2.3 (a) and 92.1 (b) cells expressing doxycycline-inducible non-targeting (NT), p300-targeting, or CBP-targeting shRNAs. Cells were treated with doxycycline (200 ng/ml) for 72 h for immunoblot analysis, or for 14 days for colony-formation assays. Data are presented as mean ± SD (n = 3 biological replicates) from one representative experiment out of three. Statistical significance was determined by a two-sided Student's *t*-test.

c and d, Immunoblot analysis and colony-formation assays in OMM2.3 (c) and 92.1 (d) cells transfected with negative control (NC), p300-targeting, CBP-targeting, or combined p300- and

CBP-targeting siRNAs. Knockdown efficiency was assessed by immunoblotting at 48 h after transfection, and cell growth was evaluated by colony-formation assay. Data are presented as mean ± SD (n = 3 biological replicates) from one representative experiment out of three. Statistical significance was determined by one-way ANOVA followed by Tukey's multiple comparisons test. Exact *P* values are indicated in the graphs; values below 0.0001 are reported as $P < 0.0001$. Source data are provided as a Source Data file.

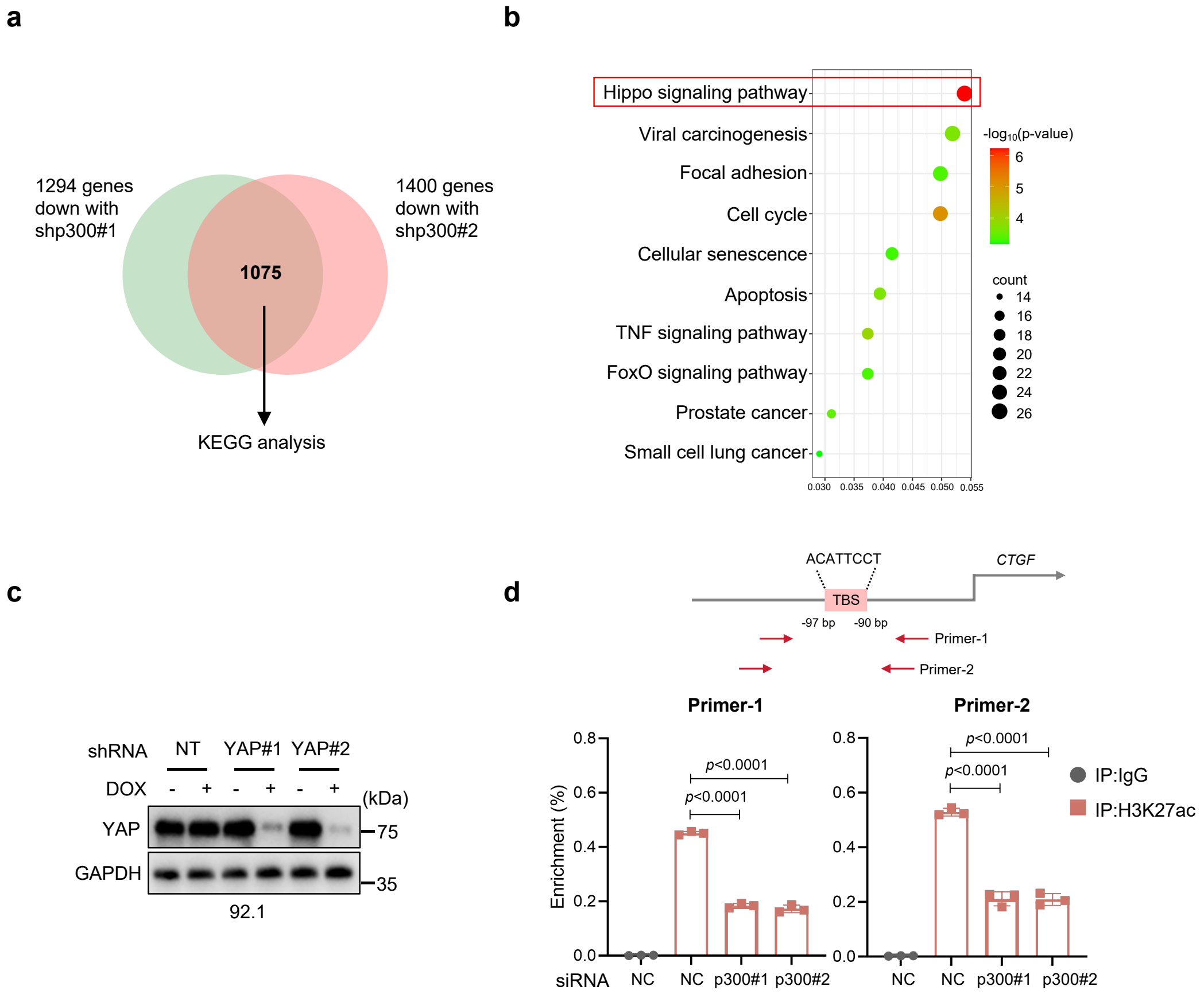

Supplementary Fig. 4: p300 knockdown modulates the Hippo pathway.

a, RNA-seq analysis identified significantly downregulated genes by p300 knockdown in 92.1 cells (adjusted p < 0.05 and fold-change < 0.75). RNA-seq was performed with 3 biological replicates.

b, KEGG pathway enrichment analysis for the significantly downregulated genes identified in **a** (n = 1075 genes).

c, Inducible knockdown of YAP in Gαq-mutant 92.1 cells. Stable 92.1 cells expressing inducible non-targeting (NT) or two YAP-targeting shRNAs (shYAP#1 and shYAP#2) were treated with or without doxycycline (200 ng/ml) for 72 h, and YAP knockdown was assessed by immunoblotting.

d, H3K27ac ChIP-qPCR analysis at the endogenous *CTGF* promoter in OMM2.3 cells transfected with the indicated siRNAs. Upper panel, schematic of the *CTGF* promoter region and primer locations. Cells were harvested 48 h after transfection. Data are presented as mean ± SD (n = 3 biological replicates). Statistical significance was assessed by one-way ANOVA followed by Tukey's multiple comparisons test. Experiments in panels (**c**, **d**) were repeated three times with similar results.

P values below 0.0001 are reported as *P* < 0.0001. Source data are provided as a Source Data file.

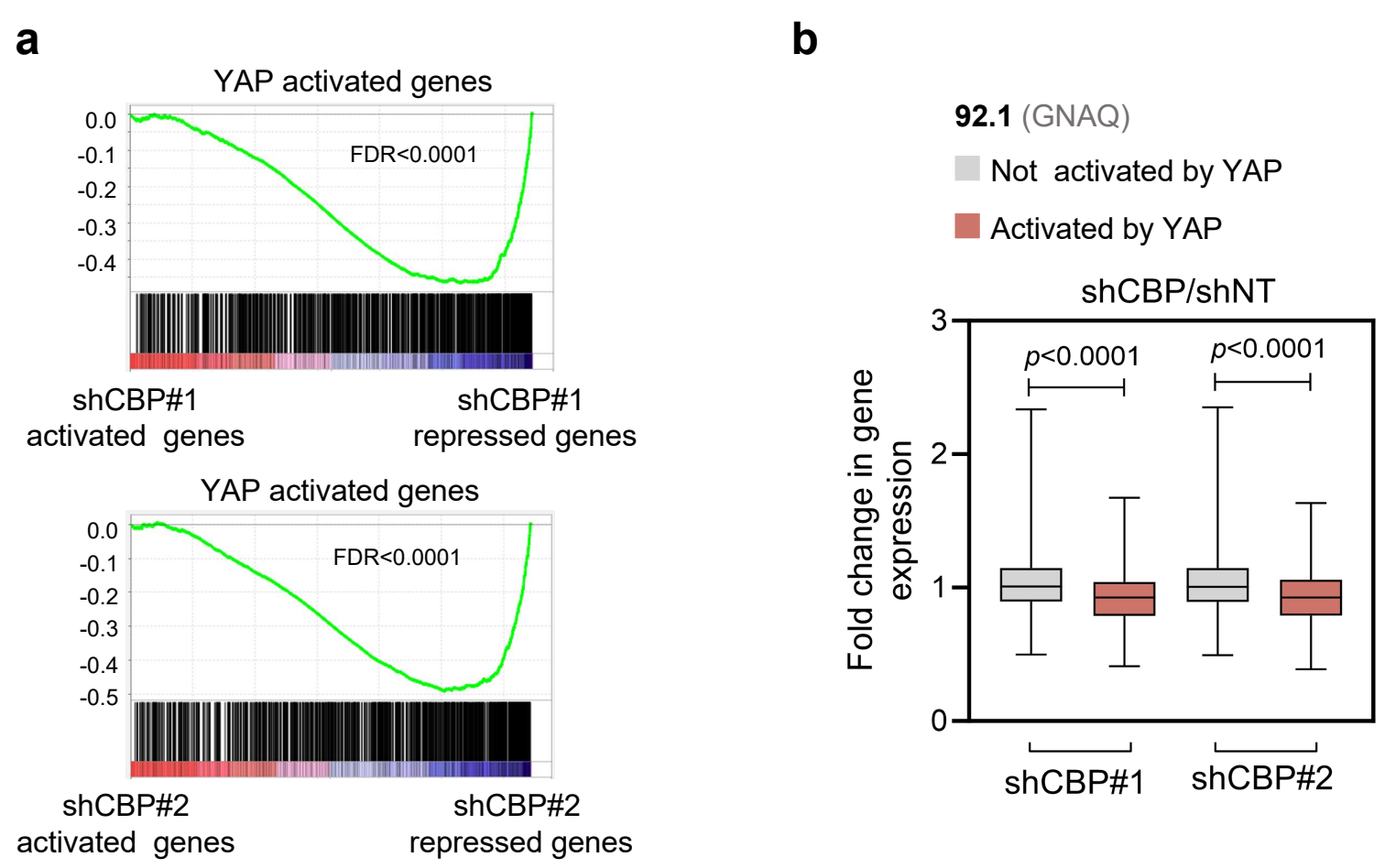

Supplementary Fig. 5: CBP depletion suppresses the YAP-activated transcriptional program.

a, Gene Set Enrichment Analysis (GSEA) showing that most YAP-activated genes were suppressed upon CBP knockdown in 92.1 cells.

b, Fold change in expression of non-YAP-activated genes (n = 9,339 genes) and YAP-activated genes (n = 1,186 genes) upon CBP knockdown in 92.1 cells, relative to cells transfected with NT shRNA. Boxes indicate the 25th to 75th percentiles, center lines indicate the medians, and whiskers indicate the 2.5th to 97.5th percentiles. Statistical significance was assessed using a two-sided Mann-Whitney U test.

P values below 0.0001 are reported as $P < 0.0001$. Source data are provided as a Source Data file.

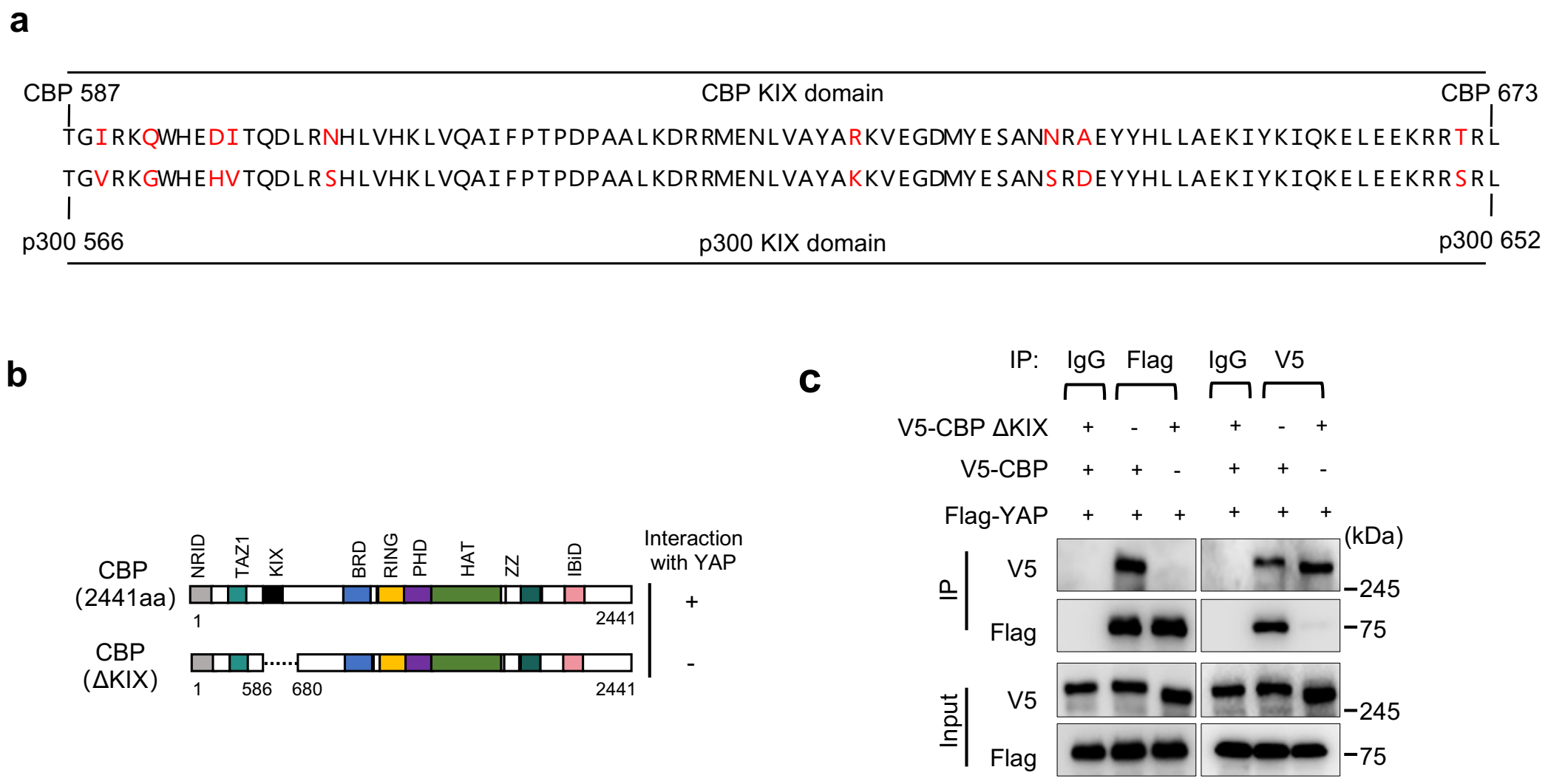

Supplementary Fig. 6: CBP interacts with YAP through its KIX domain.

a, Amino acid sequence alignment of the KIX domains of CBP and p300.

b, Schematic diagram of the V5-tagged CBP construct used in this study.

c, The KIX domain of CBP mediates its interaction with YAP. HEK293T cells were transfected with the indicated constructs. YAP or CBP was immunoprecipitated with anti-Flag or anti-V5 antibodies, respectively, and co-immunoprecipitated proteins were detected by immunoblotting with the indicated antibodies. Experiments were repeated three times with similar results.

Source data are provided as a Source Data file.

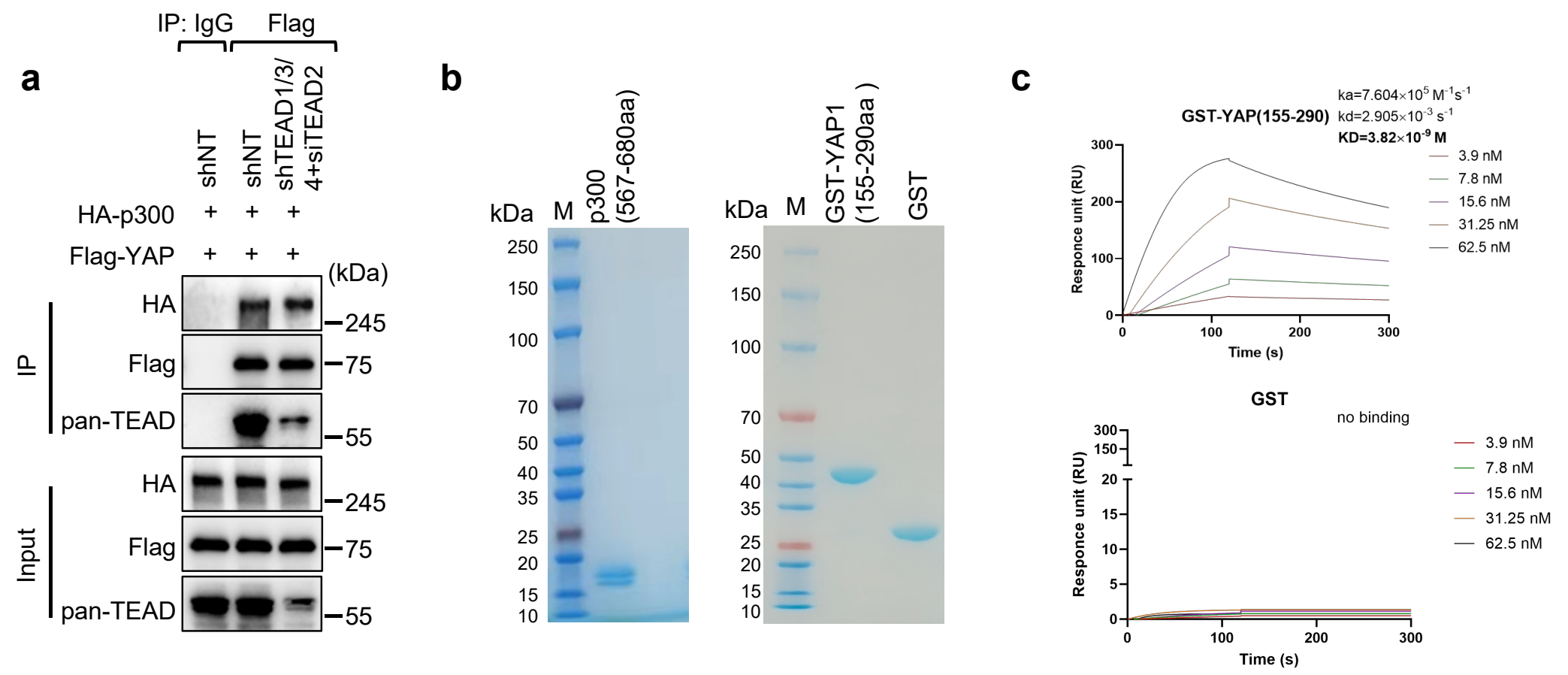

Supplementary Fig. 7: YAP directly binds p300 in a TEAD-independent manner.

a, TEAD knockdown does not impair the interaction between YAP and p300. HEK293T cells stably expressing shRNAs targeting TEAD1, 3, and 4 were transfected with TEAD2 siRNA together with YAP and p300 constructs. Cells were harvested 48 h after transfection and subjected to co-immunoprecipitation followed by immunoblotting with the indicated antibodies. Cells expressing non-targeting shRNA (shNT) served as controls.

b, SDS-PAGE analysis of purified recombinant proteins used for surface plasmon resonance (SPR) assays.

c, SPR analysis of the interaction between recombinant p300 (567-680 aa) and GST-YAP (155-290 aa). p300 bound GST-YAP in a concentration-dependent manner, whereas no binding was detected with GST alone. Experiments in panels (**a-c**) were repeated twice or more.

Source data are provided as a Source Data file.

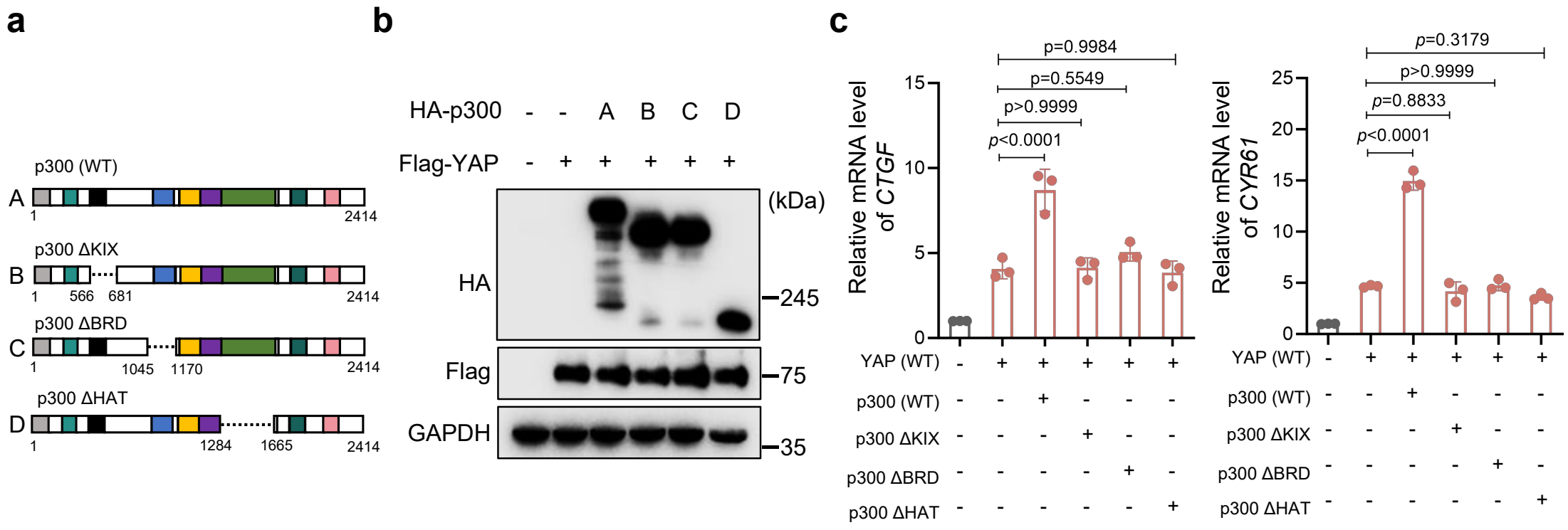

Supplementary Fig. 8: Deletion of the KIX, BRD, or HAT domain impairs p300-mediated induction of YAP target gene expression.

a, Schematic representation of the HA-tagged wild-type and domain-deletion p300 constructs used in this study.

b and c, Deletion of the KIX, BRD, or HAT domain impairs the ability of p300 to promote the expression of YAP target genes. HEK293T cells were transfected with the indicated constructs for 48 h. Expression of the p300 constructs was confirmed by immunoblotting (**b**), and mRNA levels of the YAP target genes *CTGF* and *CYR61* were measured by qPCR (**c**). Data are presented as the mean ± SD (n = 3 biological replicates) from one representative experiment out of three. Statistical significance was determined by one-way ANOVA followed by Tukey's multiple comparisons test. Exact P values are indicated in the graphs; values below 0.0001 are reported as $P < 0.0001$. Source data are provided as a Source Data file.

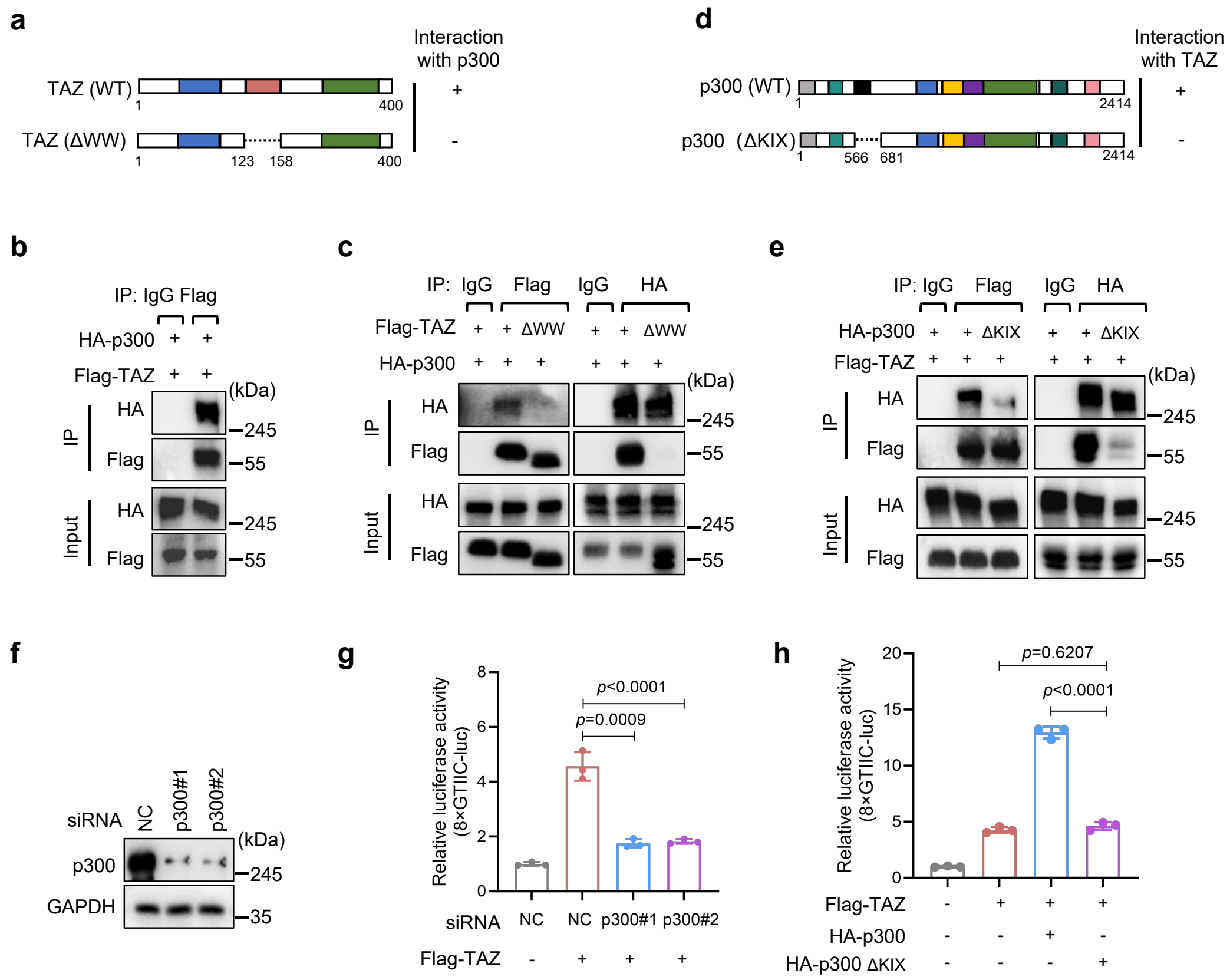

Supplementary Fig. 9: p300 interacts with TAZ and enhances TAZ-dependent transcription.

a, Schematic diagram of Flag-tagged TAZ constructs used in this study.

b, Co-immunoprecipitation (co-IP) assays showing the interaction between p300 and TAZ. HEK293T cells were transfected with the indicated constructs. TAZ was immunoprecipitated with anti-Flag antibody, and co-immunoprecipitated p300 was detected by immunoblotting with the indicated antibodies.

c, The WW domain of TAZ mediates its interaction with p300. HEK293T cells were transfected with HA-tagged p300 together with Flag-tagged full-length or WW-deleted TAZ constructs, followed by co-IP and immunoblotting as in panel **b**.

d, Schematic diagram of HA-tagged p300 constructs used in this study.

e, The KIX domain of p300 mediates its interaction with TAZ. HEK293T cells were transfected with Flag-tagged TAZ together with HA-tagged wild-type or KIX-deleted p300 constructs, followed by co-IP and immunoblotting as in panel **b**.

f and g, p300 knockdown suppresses TAZ-dependent transcription. HEK293T cells were transfected with negative control siRNA (NC) or two independent siRNAs targeting p300 (p300#1

and p300#2). Twenty-four hours later, cells were co-transfected with TAZ and the 8 × GTIIC-Luc reporter. At 72 h after the initial siRNA transfection, p300 knockdown efficiency was assessed by immunoblotting (**f**), and luciferase activities were measured (**g**). Firefly luciferase activity was normalized to Renilla luciferase activity. Data are presented as mean ± SD (n = 3 biological replicates). Statistical significance was assessed by one-way ANOVA followed by Tukey's multiple comparisons test.

h, The KIX domain is required for p300-mediated enhancement of TAZ-dependent transcription. HEK293T cells were transfected with the 8 × GTIIC-Luc reporter together with TAZ alone or in combination with p300 or p300-ΔKIX. Firefly luciferase activity was normalized to Renilla luciferase activity. Data are presented as mean ± SD (n = 3 biological replicates). Statistical significance was assessed by one-way ANOVA followed by Tukey's multiple comparisons test. Experiments in panels (**b-h**) were repeated three times with similar results.

Exact *P* values are indicated in the graphs; values below 0.0001 are reported as $P < 0.0001$. Source data are provided as a Source Data file.

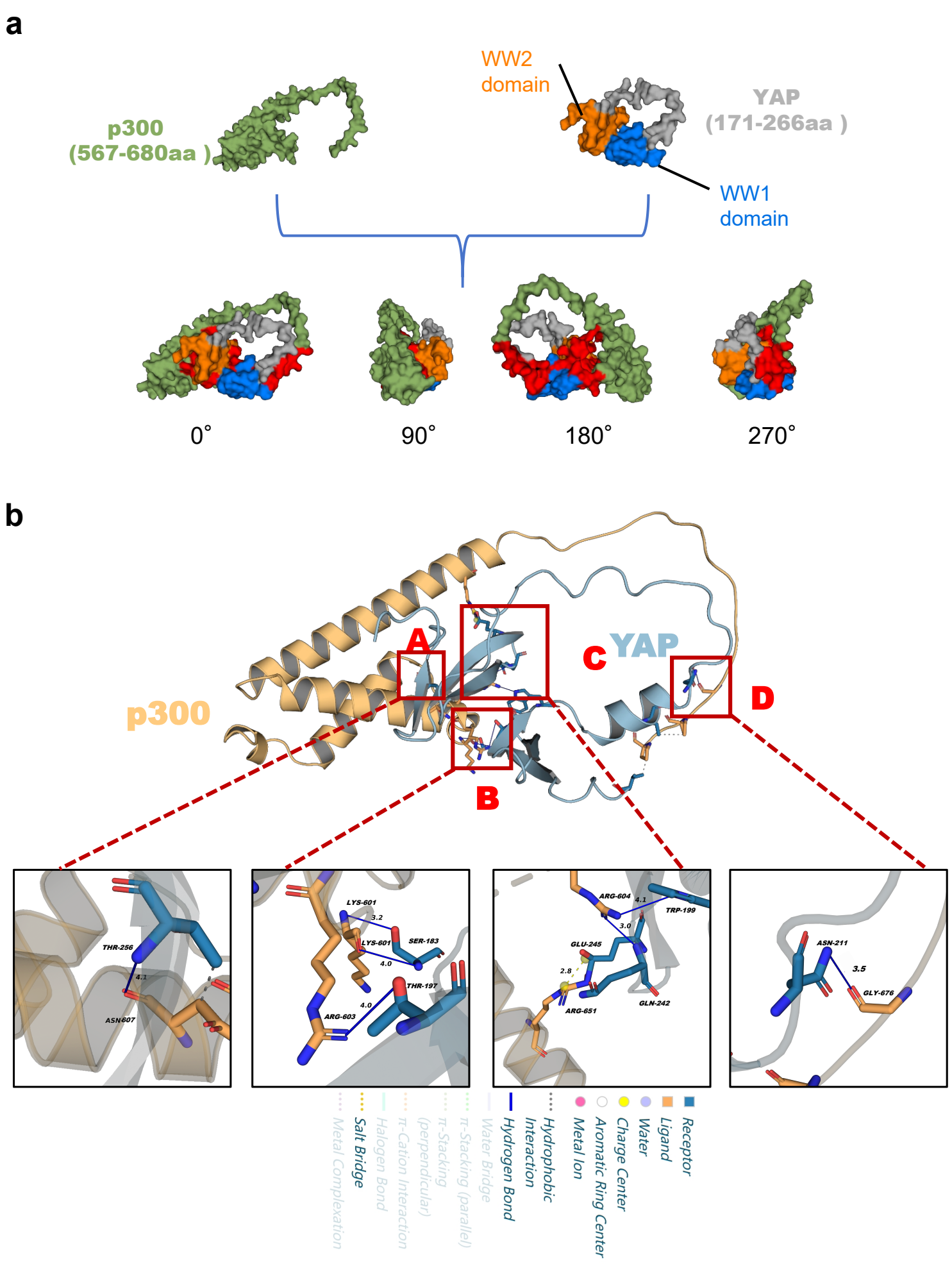

Supplementary Fig. 10: Structural modeling of the p300-YAP complex.

a, Structural model of the p300-YAP complex generated by protein-protein docking using AlphaFold3-predicted structures of p300 (567-680 aa) and YAP (171-266 aa).

b, Structural analysis of the modeled p300-YAP complex. The interaction is predicted to involve seven hydrogen bonds, one salt bridge, and hydrophobic contacts.

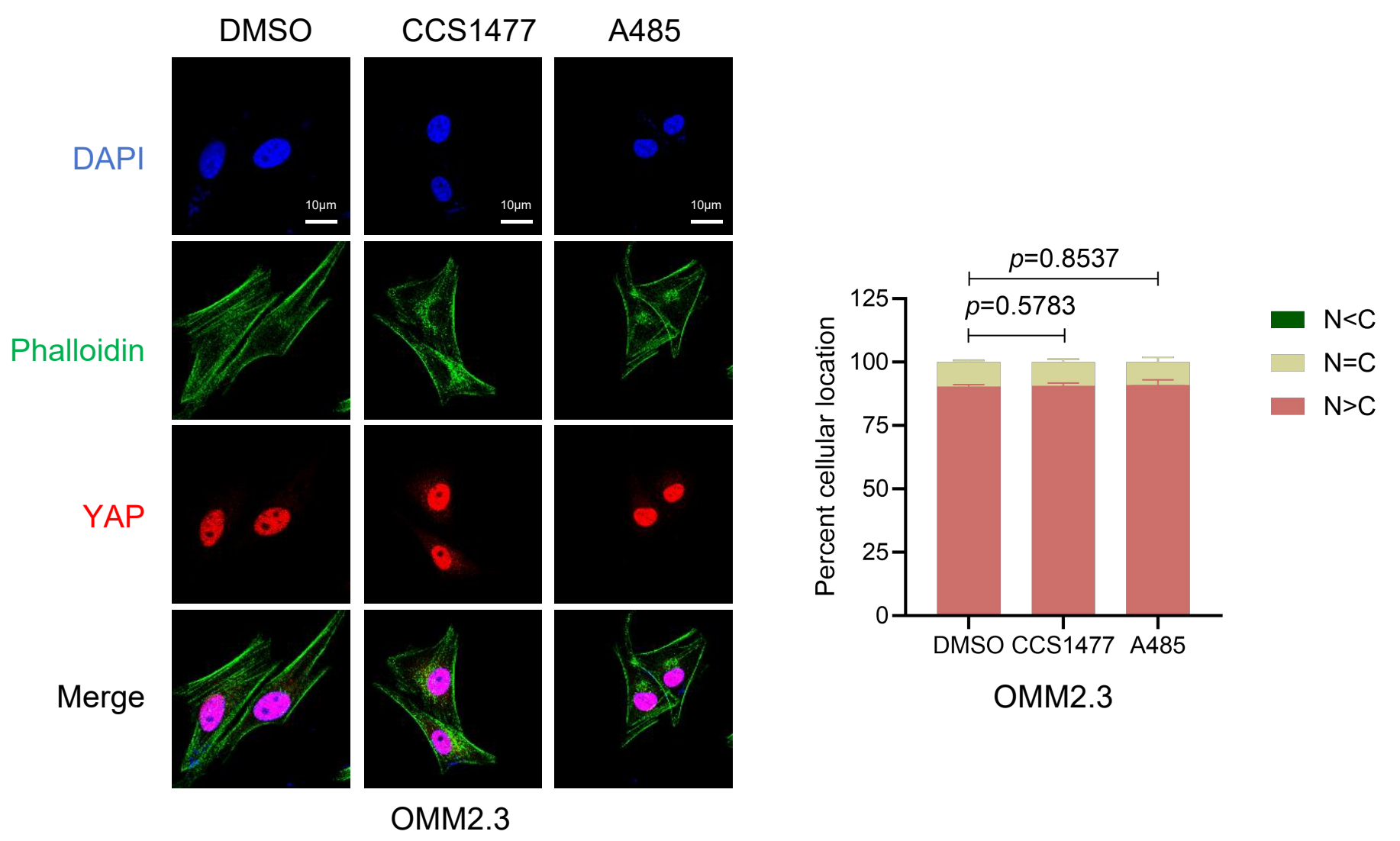

Supplementary Fig. 11: p300 inhibitors do not alter the intracellular localization of YAP in OMM2.3 cells.

OMM2.3 cells were treated with DMSO, CCS1477 (2 μM), or A485 (2 μM) for 48 h, followed by immunofluorescence staining for YAP. Nuclei were counterstained with DAPI, and F-actin was visualized with phalloidin. Scale bar, 10 μm. The percentages of cells showing predominantly nuclear (N > C), comparable nuclear and cytoplasmic (N = C), or predominantly cytoplasmic (N < C) YAP localization were quantified (n = 3 biological replicates). Statistical significance was assessed by one-way ANOVA followed by Tukey's multiple comparisons test.

Exact *P* values are indicated in the graphs. Source data are provided as a Source Data file.

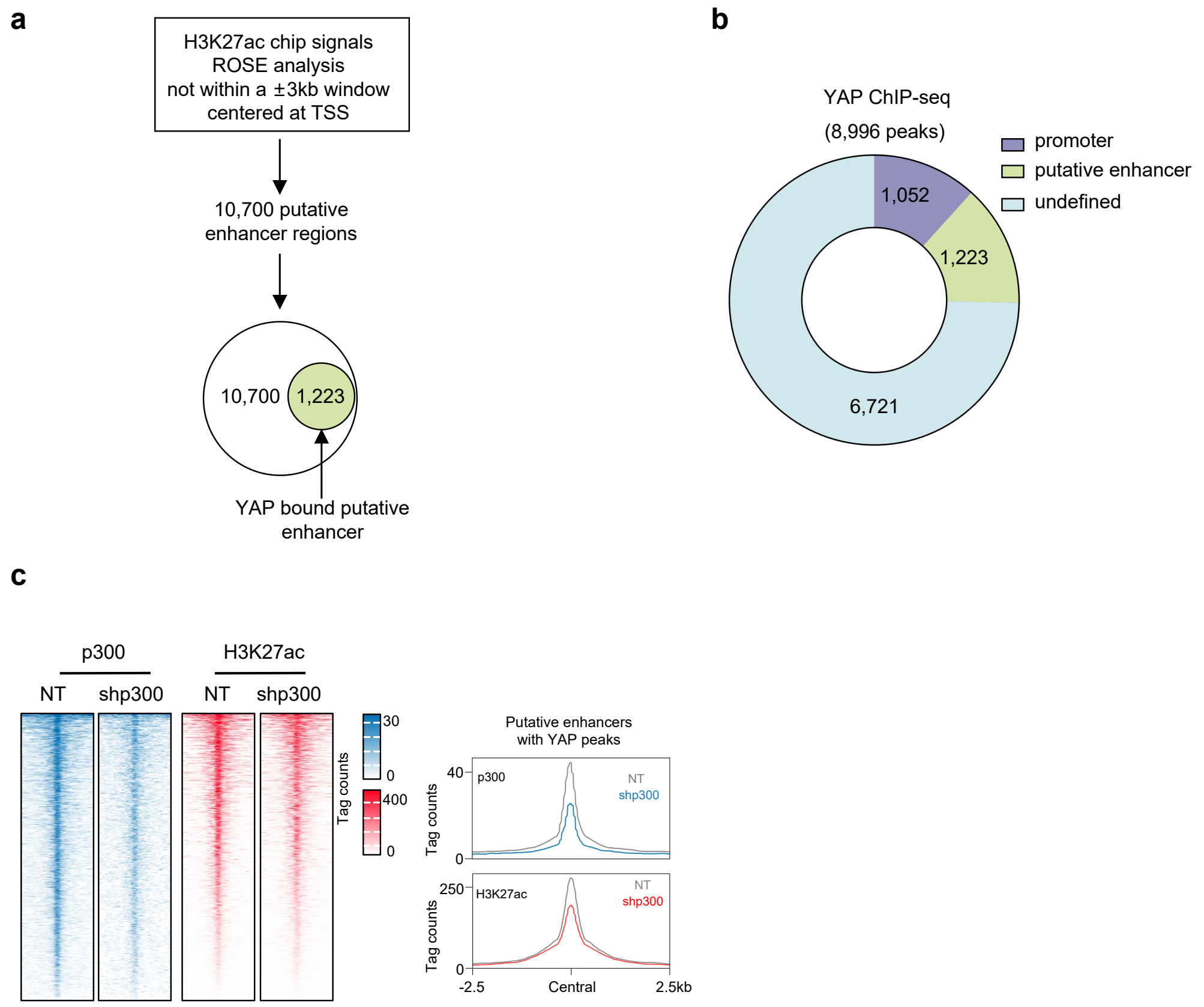

Supplementary Fig. 12: YAP occupies promoters and putative enhancers in 92.1 cells.

a, Identification of 10,700 putative enhancers in 92.1 cells based on H3K27ac ChIP-seq signals.

b, Pie chart showing the distribution of YAP-binding sites at promoter and proximal putative enhancer regions in 92.1 cells.

c, Heatmaps (left) and average line plots (right) showing p300 and H3K27ac ChIP-seq signals at YAP-bound putative enhancers in 92.1 cells, with or without p300 depletion.

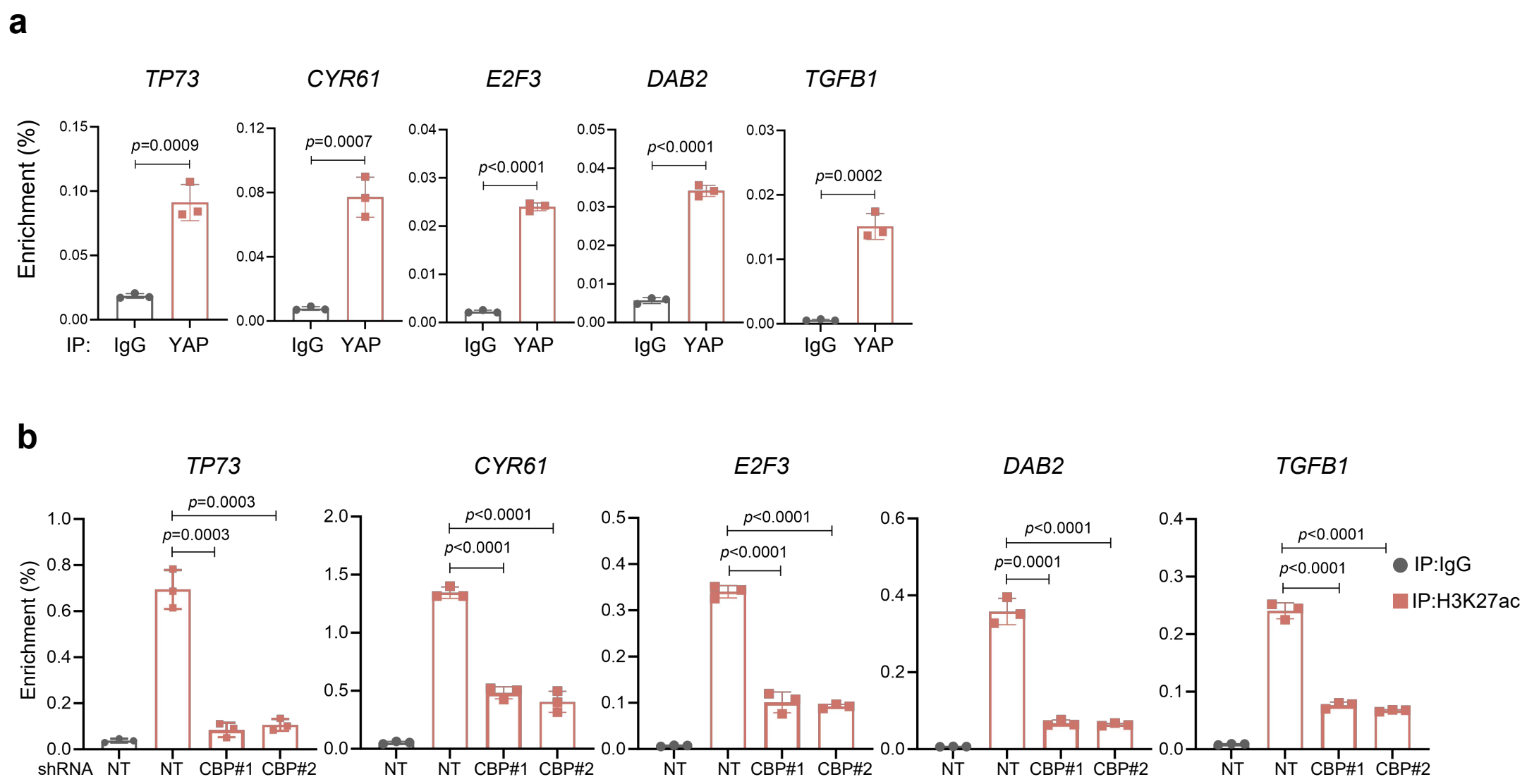

Supplementary Fig. 13: CBP knockdown reduces H3K27ac enrichment at promoters of YAP target genes.

a, ChIP-qPCR analysis showing YAP occupancy at the promoters of selected YAP target genes, including *TP73*, *CYR61*, *E2F3*, *DAB2*, and *TGFB1*. Data are presented as mean ± SD (n = 3 biological replicates) from one representative experiment out of three. Statistical significance was determined by a two-sided Student's *t*-test.

b, ChIP-qPCR analysis showing that CBP knockdown reduces H3K27ac enrichment at the promoters of YAP target genes, including *TP73*, *CYR61*, *E2F3*, *DAB2*, and *TGFB1*. Data are presented as mean ± SD (n = 3 biological replicates) from one representative experiment out of three. Statistical significance was assessed by one-way ANOVA followed by Tukey's multiple comparisons test.

Exact P values are indicated in the graphs; values below 0.0001 are reported as $P < 0.0001$. Source data are provided as a Source Data file.

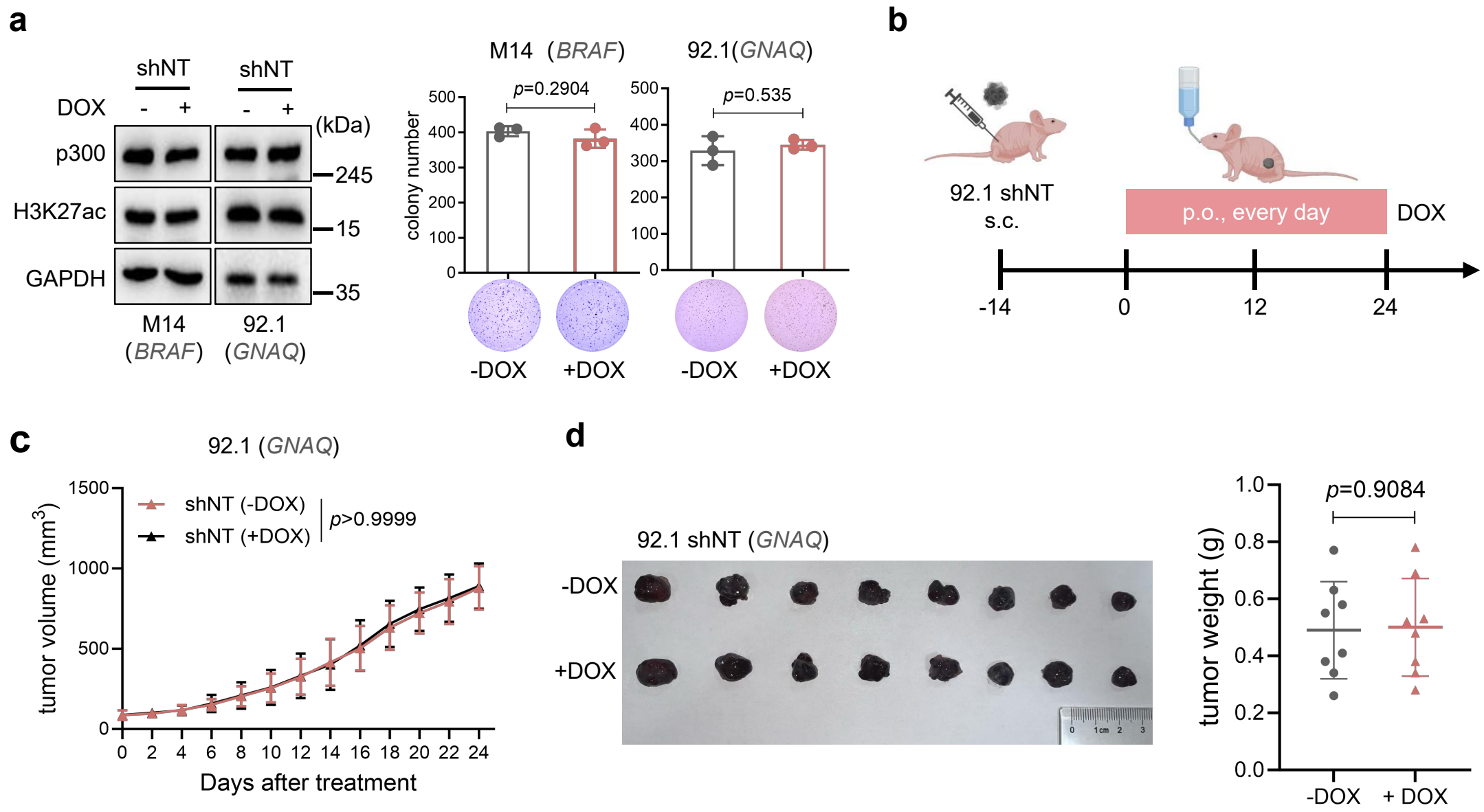

Supplementary Fig. 14: Inducible expression of non-targeting shRNA does not affect cell growth under doxycycline treatment.

a, Inducible expression of non-targeting shRNA (shNT) does not affect anchorage-independent growth of 92.1 or M14 cells. Stable 92.1 and M14 cell lines expressing inducible shNT were generated. Cells were treated with or without doxycycline (200 ng/ml) for 72 h, and p300 protein levels were assessed by immunoblotting (left). Anchorage-independent growth was assessed by soft-agar colony-formation assay after 14 days of doxycycline treatment (right). Data are presented as mean ± SD (n = 3 biological replicates) from one representative experiment out of three. Statistical significance was assessed using a two-sided Student's *t*-test. ns, not significant.

b, Schematic diagram of the xenograft experiments. Created in BioRender. Zhang, G. (2026) https://BioRender.com/81wq207.

c, Growth curves of xenografts derived from stable 92.1 cells expressing inducible shNT after subcutaneous implantation into nude mice treated with or without doxycycline (0.5 mg/ml in drinking water). Data are presented as mean ± SD (n = 8 mice per group). Statistical significance was assessed using two-way repeated-measures ANOVA.

d, Weights of 92.1-derived xenograft tumors harvested on day 24 after treatment. Data are presented as mean ± SD (n = 8 mice per group). Statistical significance was assessed using a two-sided Student's *t*-test.

Exact *P* values are indicated in the graphs. Source data are provided as a Source Data file.

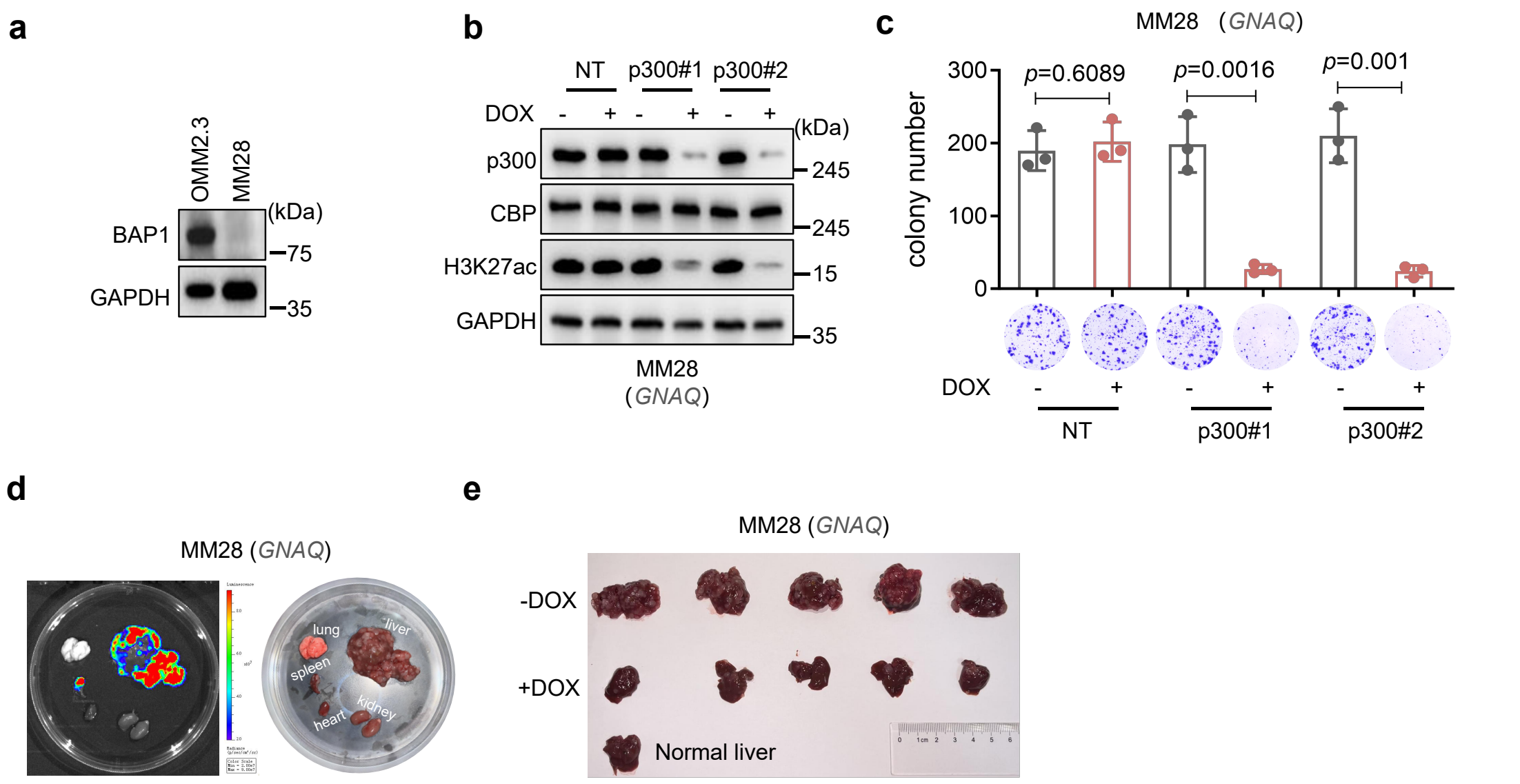

Supplementary Fig. 15: p300 knockdown suppresses *in vitro* growth and liver metastasis of Gαq-mutant, BAP1-deficient UM MM28 cells.

a, Immunoblotting analysis of BAP1 expression in MM28 cells, with OMM2.3 cells used as a control.

b, Inducible knockdown of p300 in MM28 cells. Stable MM28 cells expressing inducible non-targeting shRNA (shNT) or two independent p300-targeting shRNAs (shp300#1 and shp300#2) were generated. Cells were treated with or without doxycycline (200 ng/ml) for 72 h, and p300 knockdown was assessed by immunoblotting.

c, p300 knockdown inhibits clonogenic growth of MM28 cells. Stable MM28 cells expressing inducible shNT or p300-targeting shRNAs were treated with or without doxycycline (200 ng/ml), and clonogenic growth was assessed by colony-formation assay. Data are presented as mean ± SD (n = 3 biological replicates) from one representative experiment out of three. Statistical significance was determined by two-sided Student's *t*-test; ns, not significant.

d, Representative *ex vivo* images of organs collected at the experimental endpoint from mice bearing MM28-Luc metastases.

e, Images of livers collected from the mice bearing MM28-Luc metastases.

Exact *P* values are indicated in the graphs. Source data are provided as a Source Data file.

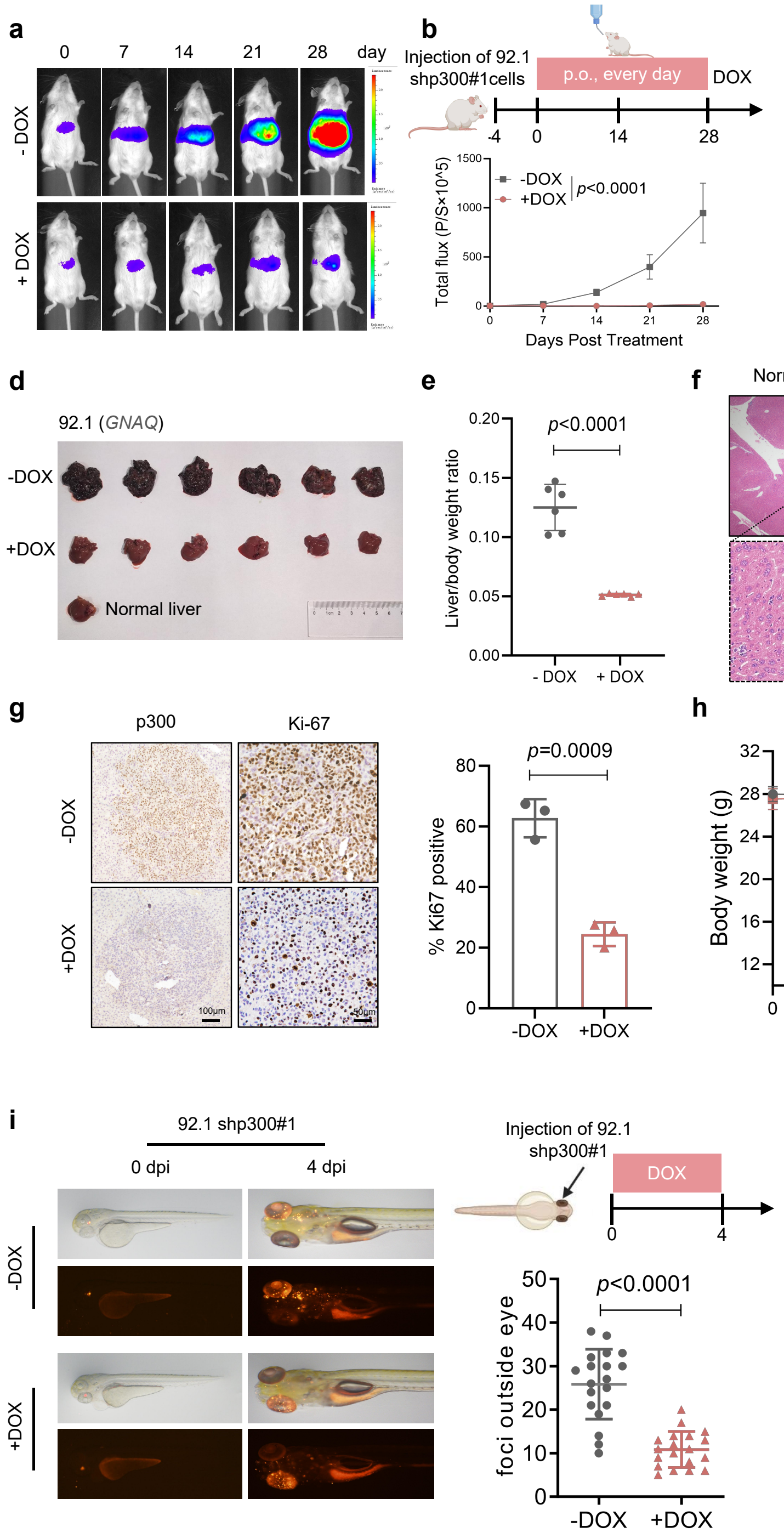

Supplementary Fig. 16: p300 knockdown suppresses metastatic progression of Gαq-mutant UM 92.1 cells.

a and b, Representative IVIS images (**a**) and quantitative analysis of tumor burden (**b**) in NCG mice injected with stable 92.1-Luc cells expressing inducible p300 shRNA#1 and treated with or without doxycycline (0.5 mg/ml in drinking water). Data are presented as mean ± SD (n = 6 mice per group). Statistical significance was assessed by two-way repeated-measures ANOVA. Created in BioRender. Zhang, G. (2026) https://BioRender.com/690qbhs.

c, Representative *ex vivo* images of organs collected at the experimental endpoint from mice bearing 92.1-Luc metastases.

d, Images of livers collected from mice bearing 92.1-Luc metastases.

e, Liver-to-body weight ratios in mice bearing 92.1-Luc metastases. Data are presented as mean ± SD (n = 6 mice per group). Statistical significance was assessed using a two-sided Student's *t*-test.

f, Representative H&E staining of liver sections from mice bearing 92.1-Luc metastases.

g, Representative immunohistochemical staining of liver sections from mice bearing 92.1-Luc metastases. Quantification is shown on the right. Data are presented as mean ± SD (n = 3 biological replicates). Statistical significance was assessed using a two-sided Student's *t*-test.

h, Body weights of mice bearing 92.1-Luc metastases. Data are presented as mean ± SD (n = 6 mice per group). Statistical significance was assessed by repeated-measures two-way ANOVA.

i, Representative images of zebrafish embryos at 0 and 4 days post-injection (dpi). Doxycycline-induced p300 knockdown suppressed dissemination of fluorescently labeled 92.1 cells in the orthotopic zebrafish embryo model. Zebrafish embryos were treated with or without doxycycline (10 µg/ml). Created in BioRender. Zhang, G. (2026) https://BioRender.com/fq3id31. Each dot represents the number of foci outside the engrafted eye in a single embryo. Data are presented as mean ± SD (n = 19 embryos per group). Statistical significance was assessed using a two-sided Student's *t* test.

Exact *P* values are indicated in the graphs; values below 0.0001 are reported as $P < 0.0001$. Source data are provided as a Source Data file.

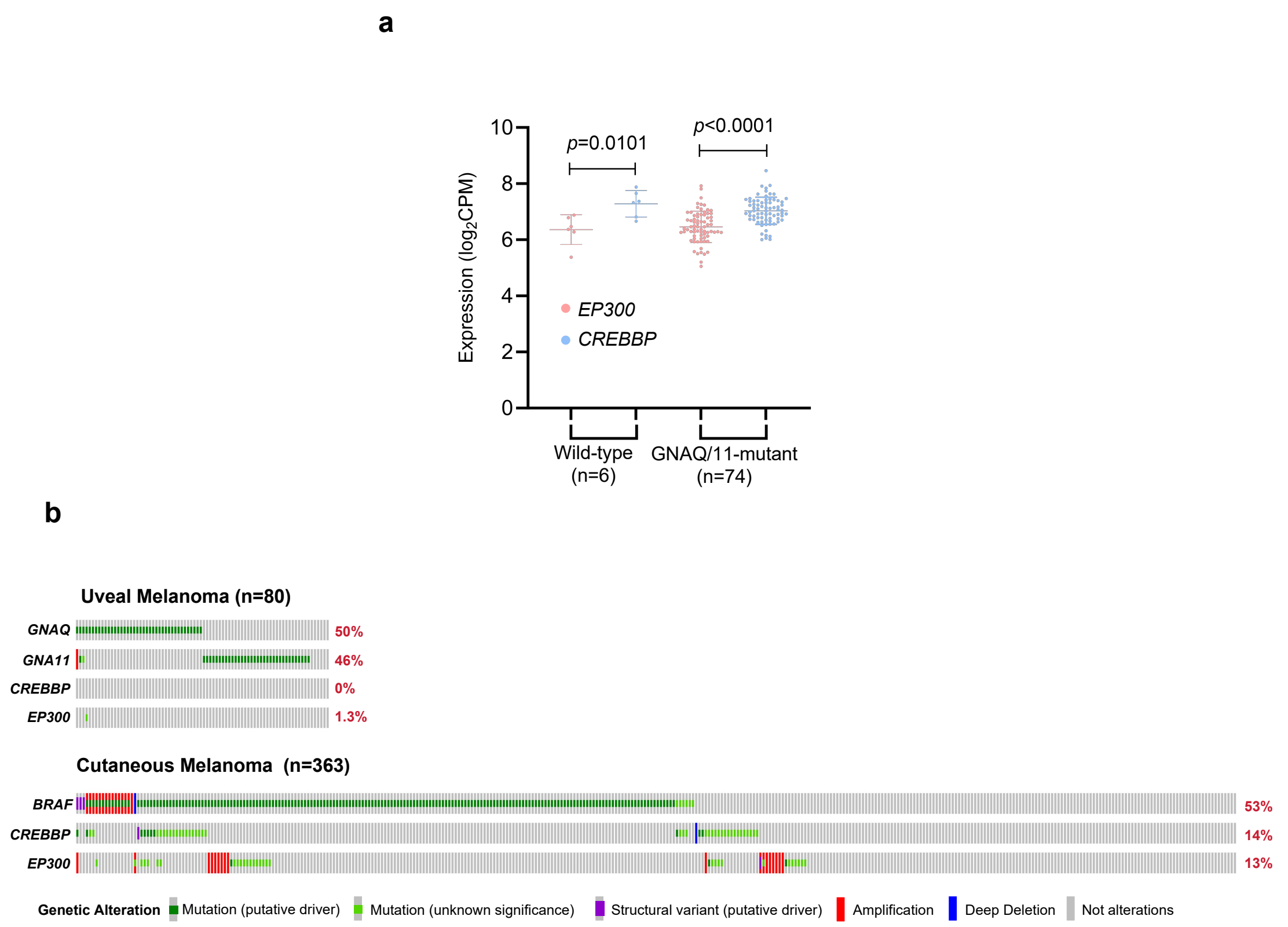

Supplementary Fig. 17: Expression and genomic landscape of *EP300* and *CREBBP* in uveal melanoma and skin cutaneous melanoma.

a, Expression levels of *EP300* and *CREBBP* in TCGA UM cohort (n = 80 patients), based on data from cBioPortal. Normality was assessed using the Shapiro-Wilk test, and normally distributed data were analyzed by two-sided Student's *t*-test.

b, Oncoprint of the genomic landscape of *EP300* and *CREBBP* in TCGA UM cohort (n = 80 patients) and skin cutaneous melanoma cohort (n = 363 patients), based on data from cBioPortal.

Exact *P* values are indicated in the graphs; values below 0.0001 are reported as $P < 0.0001$. Source data are provided as a Source Data file.

a

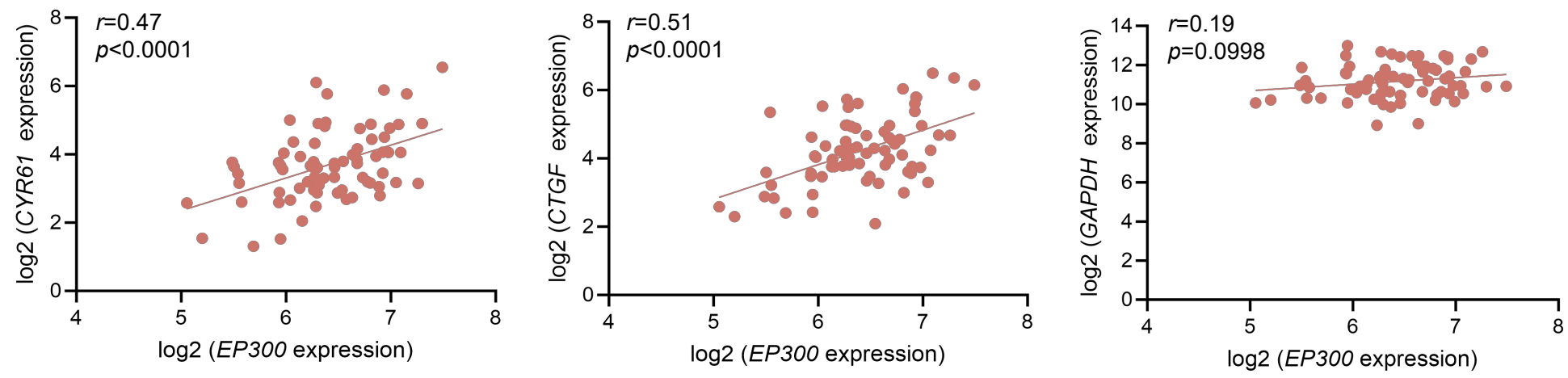

b

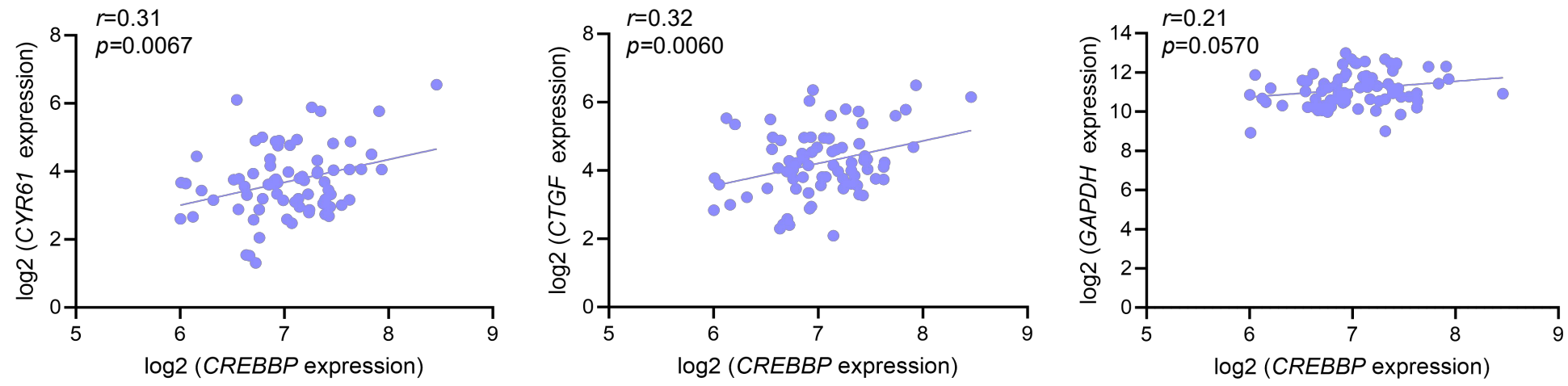

Supplementary Fig. 18: *EP300* and *CREBBP* expression correlate with YAP target gene expression in *GNAQ/11*-mutant UM patients.

a and b, *EP300* and *CREBBP* expression levels positively correlate with the expression of the YAP target genes (*CYR61* and *CTGF*) in *GNAQ/11*-mutant uveal melanoma (TCGA, n = 74 patients). *GAPDH* was included as a negative control. Statistical significance was assessed using a two-sided Pearson correlation test.

Exact P values are indicated in the graphs; values below 0.0001 are reported as $P < 0.0001$. Source data are provided as a Source Data file.

Supplementary Table 1: Targeting sequences of shRNA or siRNA.

Name	Targeted sequences
Non targeting	AAGGCCAGACGCGAATTAT
shp300#1 (*Homo sapiens*)	AGCTACTGAAGATAGATTA
shp300#2 (*Homo sapiens*)	CAATTCCGAGACATCTTGAGA
shCBP#1 (*Homo sapiens*)	GCTATCAGAATAGGTATCATT
shCBP#2 (*Homo sapiens*)	GCGTTTACATAAACAAGGCAT
shYAP#1 (*Homo sapiens*)	CTGGTCAGAGATACTTCTTAA
shYAP#2 (*Homo sapiens*)	AAGCTTTGAGTTCTGACATCC
shGαq#1 (*Homo sapiens*)	CCTCGGTTATTCTGTTCTTAA
shGαq#2 (*Homo sapiens*)	GCACAATTAGTTCGAGAAGTT
shTEAD1/3/4 (*Homo sapiens*)	ATGATCAACTTCATCCACAAG
sip300#1 (*Homo sapiens*)	GGAUUCGUCUGUAUGGCUGGUUUAA
sip300#2 (*Homo sapiens*)	GCAAAGGAAUUGCCUUAUU
siCBP#1 (*Homo sapiens*)	GAGGUCGUUUACAUAAA
siCBP#2 (*Homo sapiens*)	GGAUCUCAGAAUGACUUAA
siYAP (*Homo sapiens*)	GACAUCUUCUGGUCAGAGA
siTAZ (*Homo sapiens*)	AGGUACUUCCUCAAUCACA
siTEAD2 (*Homo sapiens*)	GUGGUGAAUUUCUUGCACA

Supplementary Table 2. Primers used for ChIP-qPCR analysis.

Name	Primer sequences
CTGF-Primer-1 (*Homo sapiens*)	AATCCGGTGTGAGTTGATGA<br>CTGAATGGAGTCCTACACAA
CTGF-Primer-2 (*Homo sapiens*)	AGTTGATGAGGCAGGAAGGT<br>AGGCTTTTATACGCTCCGGG
CYR61 (*Homo sapiens*)	AAAGGTGCAATGGAGCCAGG<br>CCCGGAGCCCGCCTTTTATA
TP73 (*Homo sapiens*)	TTCTGAGGACCAGACAGTG<br>TGTCCGGGGTTGGAGGGTT
E2F3 (*Homo sapiens*)	ACATTTGCACTTTGTGGGTC<br>TCATCATCTGATTGCTGC
DAB2 (*Homo sapiens*)	TTAAGTTTGGAAGACTCTGC<br>GCGAGAGATATGGTTCTAATCA
TGFB1 (*Homo sapiens*)	CAATGGGCTGCCCTGACATG<br>TCAGTATAAAGTCCAACCTC
