# Supplementary material for: Synthetic lethal screens reveal p300/CBP as a vulnerability for GNAQ-driven uveal melanomas
Source: Nat Commun. 2026 Oct 2;17:11303. doi: 10.1038/s41467-026-77999-8 (PMC13724638; doi:10.1038/s41467-026-77999-8)
Supplement: Supplementary file 2 [file 41467_2026_77999_MOESM2_ESM.pdf]

nature portfolio

Corresponding author(s): Le Yu, Yi-Lei Li, Zhijie Li, Chongzhi Bai

Last updated by author(s): Jul 21, 2026

Reporting Summary

Nature Portfolio wishes to improve the reproducibility of the work that we publish. This form provides structure for consistency and transparency in reporting. For further information on Nature Portfolio policies, see our Editorial Policies and the Editorial Policy Checklist.

Statistics

For all statistical analyses, confirm that the following items are present in the figure legend, table legend, main text, or Methods section.

n/a	Confirmed	
☐	☒	The exact sample size (*n*) for each experimental group/condition, given as a discrete number and unit of measurement
☐	☒	A statement on whether measurements were taken from distinct samples or whether the same sample was measured repeatedly
☐	☒	The statistical test(s) used AND whether they are one- or two-sided<br>*Only common tests should be described solely by name; describe more complex techniques in the Methods section.*
☐	☒	A description of all covariates tested
☐	☒	A description of any assumptions or corrections, such as tests of normality and adjustment for multiple comparisons
☐	☒	A full description of the statistical parameters including central tendency (e.g. means) or other basic estimates (e.g. regression coefficient) AND variation (e.g. standard deviation) or associated estimates of uncertainty (e.g. confidence intervals)
☐	☒	For null hypothesis testing, the test statistic (e.g. *F*, *t*, *r*) with confidence intervals, effect sizes, degrees of freedom and *P* value noted<br>*Give P values as exact values whenever suitable.*
☒	☐	For Bayesian analysis, information on the choice of priors and Markov chain Monte Carlo settings
☒	☐	For hierarchical and complex designs, identification of the appropriate level for tests and full reporting of outcomes
☐	☒	Estimates of effect sizes (e.g. Cohen's *d*, Pearson's *r*), indicating how they were calculated

Our web collection on statistics for biologists contains articles on many of the points above.

Software and code

Policy information about availability of computer code

Data collection	Illumina FASTQ Toolkit for sequencing.
Data analysis	GraphPad (v 10.0), Excel (v 16.78), ImageJ (v 2.0.0), MAGeCK RRA statistical (v 0.5.9), Hisat2 (v 2.0.1), FeatureCounts (v 2.0.3), FastQC (v 0.11.9), Trim_galore (v 0.6.10), Bowtie2 (v 0.12.7), samtools (v 1.7), bedtools (v2.31.1), MACS2 (v 3.0.2), deepTools (v 3.1.3), IGV (v2.17.4), R (v 4.3.2), R packages: EdgeR (v4.8.2), DESeq2 (v1.50.2), ggplot2 (v4.0.2), ChIPseeker (v1.46.1).

For manuscripts utilizing custom algorithms or software that are central to the research but not yet described in published literature, software must be made available to editors and reviewers. We strongly encourage code deposition in a community repository (e.g. GitHub). See the Nature Portfolio guidelines for submitting code & software for further information.

Data

Policy information about availability of data

All manuscripts must include a data availability statement. This statement should provide the following information, where applicable:

- Accession codes, unique identifiers, or web links for publicly available datasets
- A description of any restrictions on data availability
- For clinical datasets or third party data, please ensure that the statement adheres to our policy

All sequencing data are available from the NCBI SRA database with accession numbers PRJNA1175074, PRJNA1222236, PRJNA1397940 and PRJNA1397882.All other data are available within the article and its Supplementary Information. Source data are provided with this paper.

Research involving human participants, their data, or biological material

Policy information about studies with human participants or human data. See also policy information about sex, gender (identity/presentation), and sexual orientation and race, ethnicity and racism.

Reporting on sex and gender	*Use the terms sex (biological attribute) and gender (shaped by social and cultural circumstances) carefully in order to avoid confusing both terms. Indicate if findings apply to only one sex or gender; describe whether sex and gender were considered in study design; whether sex and/or gender was determined based on self-reporting or assigned and methods used. Provide in the source data disaggregated sex and gender data, where this information has been collected, and if consent has been obtained for sharing of individual-level data; provide overall numbers in this Reporting Summary. Please state if this information has not been collected. Report sex- and gender-based analyses where performed, justify reasons for lack of sex- and gender-based analysis.*
Reporting on race, ethnicity, or other socially relevant groupings	*Please specify the socially constructed or socially relevant categorization variable(s) used in your manuscript and explain why they were used. Please note that such variables should not be used as proxies for other socially constructed/relevant variables (for example, race or ethnicity should not be used as a proxy for socioeconomic status). Provide clear definitions of the relevant terms used, how they were provided (by the participants/respondents, the researchers, or third parties), and the method(s) used to classify people into the different categories (e.g. self-report, census or administrative data, social media data, etc.) Please provide details about how you controlled for confounding variables in your analyses.*
Population characteristics	*Describe the covariate-relevant population characteristics of the human research participants (e.g. age, genotypic information, past and current diagnosis and treatment categories). If you filled out the behavioural & social sciences study design questions and have nothing to add here, write "See above."*
Recruitment	*Describe how participants were recruited. Outline any potential self-selection bias or other biases that may be present and how these are likely to impact results.*
Ethics oversight	*Identify the organization(s) that approved the study protocol.*

Note that full information on the approval of the study protocol must also be provided in the manuscript.

Field-specific reporting

Please select the one below that is the best fit for your research. If you are not sure, read the appropriate sections before making your selection.

☒ Life sciences ☐ Behavioural & social sciences ☐ Ecological, evolutionary & environmental sciences

For a reference copy of the document with all sections, see nature.com/documents/nr-reporting-summary-flat.pdf

Life sciences study design

All studies must disclose on these points even when the disclosure is negative.

Sample size	No pre-determine sample-size calculations were performed. The sample size was based on the literature that tested the same cell lines or performed the similar assays.
Data exclusions	No data was excluded from the experiments.
Replication	In vitro experiments were performed at least 2 times or more. Similar observation was obtained for each replicate. Representative result was shown in the figures.
Randomization	Mice and zebrafish embryos were randomly allocated into experimental groups.
Blinding	The experiments and analysis were performed in a non-blinded fashion because the same investigator was doing group allocation during data collection and analysis. Our analyses all generate objective outcomes that are not subject to observer bias.

Reporting for specific materials, systems and methods

We require information from authors about some types of materials, experimental systems and methods used in many studies. Here, indicate whether each material, system or method listed is relevant to your study. If you are not sure if a list item applies to your research, read the appropriate section before selecting a response.

Materials & experimental systems

n/a	Involved in the study	
☐	☒	Antibodies
☐	☒	Eukaryotic cell lines
☒	☐	Palaeontology and archaeology
☐	☒	Animals and other organisms
☒	☐	Clinical data
☒	☐	Dual use research of concern
☒	☐	Plants

Methods

n/a	Involved in the study	
☐	☒	ChIP-seq
☒	☐	Flow cytometry
☒	☐	MRI-based neuroimaging

Antibodies

Antibodies used

Antibodies against YAP (#14074), TAZ (#4883), HA-tag (#3724), HA-tag HRP Conjugate (#14031), V5-tag (#13202), CBP (#7389), Acetylated-lysine (#9441), H3K27ac (#8173), H3K4me3 (#9751), Rpb1 CTD (#2629), Ki67 (#9449), pan-TEAD (#13295) were obtained from Cell Signaling Technology. Antibodies against GAPDH (#sc-25778), CDK9 (#sc-13130), p300 (#sc-48343) and Gαq (#sc-393) was purchased from Santa Cruz Biotechnology. Antibody against TEAD4 (#ab58310), YAP (#ab52771) was purchased from Abcam. Antibody against Flag tag (#F1804), p300 (#05-257) was from Sigma-Aldrich. CTGF antibody (#25474-1-AP) was purchased from Proteintech.

Validation

YAP (https://www.cellsignal.cn/products/primary-antibodies/yap-d8h1x-xp-rabbit-mab/14074)
TAZ (https://www.cellsignal.cn/products/primary-antibodies/taz-v386-antibody/4883)
HA-tag (https://www.cellsignal.cn/products/primary-antibodies/ha-tag-c29f4-rabbit-mab/3724)
HA-tag HRP Conjugate (https://www.cellsignal.cn/products/antibody-conjugates/ha-tag-c29f4-rabbit-mab-hrp-conjugate/14031)
V5-tag (https://www.cellsignal.cn/products/primary-antibodies/v5-tag-d3h8q-rabbit-monoclonal-antibody/13202)
CBP (https://www.cellsignal.cn/products/primary-antibodies/cbp-d6c5-rabbit-mab/7389)
Acetylated-lysine (https://www.cellsignal.cn/products/primary-antibodies/acetylated-lysine-antibody/9441)
H3K27ac (https://www.cellsignal.cn/products/primary-antibodies/acetyl-histone-h3-lys27-d5e4-xp-rabbit-mab/8173)
H3K4me3 (https://www.cellsignal.cn/products/primary-antibodies/tri-methyl-histone-h3-lys4-c42d8-rabbit-mab/9751)
Rpb1 CTD (https://www.cellsignal.cn/products/primary-antibodies/rpb1-ctd-4h8-mouse-mab/2629)
Ki-67 (https://www.cellsignal.cn/products/primary-antibodies/ki-67-8d5-mouse-mab/9449)
pan-TEAD (https://www.cellsignal.cn/products/primary-antibodies/pan-tead-d3f7l-rabbit-monoclonal-antibody/13295)
GAPDH (https://www.scbt.com/zh/p/gapdh-antibody-fl-335)
CDK9 (https://www.scbt.com/zh/p/cdk9-antibody-d-7)
p300 (https://www.scbt.com/zh/p/p300-antibody-f-4)
Gαq (https://www.scbt.com/p/galpha-q-antibody-e-17)
TEAD4 (https://www.abcam.cn/products/primary-antibodies/tead4-antibody-5h3-ab58310.html)
YAP (https://www.abcam.cn/products/primary-antibodies/yap1-antibody-ep1674y-ab52771.html)
Flag-tag (https://www.sigmaaldrich.cn/CN/zh/product/sigma/f1804)
p300 (https://www.sigmaaldrich.cn/CN/zh/product/mm/05257)
CTGF (https://www.ptgcn.com/Products/CTGF-Antibody-25474-1-AP.htm)

Eukaryotic cell lines

Policy information about cell lines and Sex and Gender in Research

Cell line source(s)

Human uveal melanoma cell lines (92.1, Mel202, Mel270, OMM2.3, Mel285, and Mel290), provided by Dr. Martine Jager (Leiden University). Human cutaneous melanoma cell lines (A375, M14, and SK-MEL-28) obtained from Cellcook Biotech Co.Ltd. (Guangzhou, China). Human uveal melanoma cell lines (MM28) obtained from MeisenCTCC (Zhejiang China). HEK293A and HEK293T were obtained from ATCC.

Authentication

Cell line (HEK293A and HEK293T) were originally obtained from ATCC, therefore were not authenticated. Cell lines (92.1, Mel202, Mel270, OMM2.3, Mel285, Mel290, A375, M14, MM28 and SK-MEL-28) were authenticated by STR.

Mycoplasma contamination

All cells were tested for mycoplasma and they are negative of mycoplasma contamination.

Commonly misidentified lines
(See ICLAC register)

None

Animals and other research organisms

Policy information about studies involving animals; ARRIVE guidelines recommended for reporting animal research, and Sex and Gender in Research

Laboratory animals

Nude mice (6 weeks old) were purchased from Guangdong Medical Laboratory Animal Center (Guangzhou, China), while NCG mice (6 weeks old) were purchased from Gempharmatech (Jiangsu, China). Wild-type AB zebrafish were obtained from China Zebrafish Resource Center (Wuhan, China).

Wild animals	None
Reporting on sex	None
Field-collected samples	None
Ethics oversight	All animal procedures were conducted in accordance with the protocols approved by the Institutional Animal Care and Use Committee of Southern Medical University (SMUL2021069).

Note that full information on the approval of the study protocol must also be provided in the manuscript.

Plants

Seed stocks	*Report on the source of all seed stocks or other plant material used. If applicable, state the seed stock centre and catalogue number. If plant specimens were collected from the field, describe the collection location, date and sampling procedures.*
Novel plant genotypes	*Describe the methods by which all novel plant genotypes were produced. This includes those generated by transgenic approaches, gene editing, chemical/radiation-based mutagenesis and hybridization. For transgenic lines, describe the transformation method, the number of independent lines analyzed and the generation upon which experiments were performed. For gene-edited lines, describe the editor used, the endogenous sequence targeted for editing, the targeting guide RNA sequence (if applicable) and how the editor was applied.*
Authentication	*Describe any authentication procedures for each seed stock used or novel genotype generated. Describe any experiments used to assess the effect of a mutation and, where applicable, how potential secondary effects (e.g. second site T-DNA insertions, mosiacism, off-target gene editing) were examined.*

ChIP-seq

Data deposition

☒ Confirm that both raw and final processed data have been deposited in a public database such as GEO.

☒ Confirm that you have deposited or provided access to graph files (e.g. BED files) for the called peaks.

Data access links<br>*May remain private before publication.*	The sequencing data are available from the NCBI SRA database with accession numbers PRJNA1175074, PRJNA1222236, PRJNA1397940 and PRJNA1397882.
Files in database submission	Fastq files for ChIP-seq, ATAC-seq and RNA-seq have all been deposited
Genome browser session<br>(e.g. UCSC)	Data are available at PRJNA1222236.

Methodology

Replicates	Each sample were derived from the pool of 3 biological samples.
Sequencing depth	The ChIP-seq were sequenced using NovaSeq X Plus to a depth of approximately 20 million reads, double-ended at 150bp length
Antibodies	anti-YAP Abcam ab52771<br>anti-p300 Sigma-Aldrich 05-257<br>anti-H3K4me3 Cell Signaling Technology 9751<br>anti-H3K27ac Cell Signaling Technology 8173<br>anti-Rpb1 CTD Cell Signaling Technology 2629<br>anti-CDK9 Santa Cru Biotechnology sc-13130
Peak calling parameters	Peak calling was performed with MACS (v3.0.2) with parameter -nomodel
Data quality	FastQC (v0.11.9) was used for sequencing quality control. Trim_galore (v0.6.10) for quality and adapter trimming.
Software	Bowtie2 (v0.12.7) for sequencing read mapping to hg38 genome. samtools (v1.7) for filtering uniquely mapping reads, remove blacklist region. MACS2 (v3.0.2) for peak calling, deepTools (v3.1.3) for heatmap and plot profile generation, data visualization was performed using IGV.
