# Supplementary material for: Synthetic lethal screens reveal p300/CBP as a vulnerability for GNAQ-driven uveal melanomas
Source: Nat Commun. 2026 Oct 2;17:11303. doi: 10.1038/s41467-026-77999-8 (PMC13724638; doi:10.1038/s41467-026-77999-8)
Supplement: Supplementary file 3 [file 41467_2026_77999_MOESM3_ESM.pdf]

nature portfolio

Peer Review File

Synthetic lethal screens reveal p300/CBP as a vulnerability for GNAQ-driven uveal melanomas

Corresponding Author: Professor Le YU

This file contains all reviewer reports in order by version, followed by all author rebuttals in order by version.

Version 0:

Reviewer comments:

Reviewer #1

(Remarks to the Author)
Zhang et al describe an effort to identify genetic vulnerabilities of uveal melanoma (UM) characterized by oncogenic mutations in GNAQ/GNA11. While much of the work is rigorous, well-controlled, and shows material effect sizes, there is considerable confusion about the overall flow and consistency of the paper.

In the first story, CRISPR screens targeting a small set of epigenetic regulators in isogenic (Gaq-mutant) cells identify EP300 as highly essential specifically in Gaq-mutant cells. One potential issue is that the authors use a non-UM cell line, HEK293A, with a Gaq knock in to stand in for their tumor cells of choice. This seems to have been overcome by the considerable amount of validation work applied. Notably, 9 cell lines were subjected in vitro to three inhibitors of p300, encoded by the EP300 gene, showing consistent increased sensitivity in the GNAQ backgrounds. Then we fast forward to Figure 6, which demonstrates GNAQ-dependent vulnerability to EP300 knockdown by dox-inducible shRNA, and Figure 7, which shows the same dox-inducible knockdown also decreases hepatic metastasis after splenic injection. A p300 inhibitor is also shown to be effective in significantly reducing tumor growth rate after intrasplenic injection. This is a nice coherent body of work with strong results.

In between, Figure 2 diverts to a p300/YAP interaction study, with Figure 3 characterizing p300 effect on YAP transcriptional activity, Fig 4 showing p300 inhibitors also suppress targets of YAP transcription, and Fig 5 describing overlapping ChIP-seq of YAP and p300. Although this work is fairly comprehensive, the YAP/p300 transcriptional regulatory complex has been previously described (technically a YAP–TEAD–Smad–p300 complex; Fujii JXMed 2012; Hiemer JBC 2014) and neither the novelty nor the relevance seem to put this sub-story into this larger study of Gaq-dependent genetic vulnerabilities.

In short, while the work appears to all be solid, with well-controlled experiments and robust results, the flow of the manuscript is a bit unwieldy. This reviewer understands the pressure to provide mechanism for discoveries of genetic vulnerability, but the EP300/YAP story doesn't mechanistically explain the vulnerability. This would be a perfectly legitimate and novel story with just Figures 1, 6, and 7.

Reviewer #2

(Remarks to the Author)
This study aims to identify targetable vulnerabilities for uveal melanoma through a functional genomic screen focused on epigenetic regulators. The authors propose p300 as a therapeutic target, supported by mechanistic evidence suggesting that p300 inhibition suppresses YAP/TAZ-dependent genes via direct interaction between YAP's WW domain and p300's KIX domain. While the work highlights p300's potential in treating uveal melanoma, critical evidence gaps weaken support for the central conclusions.

Major Comments
1. The observed epigenetic rewiring in HEK293A cells with Gq(Q209L) knock-in (generation process should be detailed in Methods) is intriguing. However, the relevance of this model to uveal melanoma cell transformation remains unclear. The study does not convincingly establish p300 as a synthetic lethal partner in GNAQ-driven uveal melanoma or address how

this switch reflects actual tumor biology.

2. While focusing on epigenetic regulators is justified by the low mutation burden in uveal melanoma, the authors provide no direct evidence linking epigenetic mechanisms to disease pathogenesis. This omission undermines the rationale for restricting the screen to epigenetic targets.

3. Prior studies implicate TEAD family proteins in epigenetic regulation via enhancers involving p300. The claim of direct YAP-p300 interaction (via WW-KIX domains) lacks robust validation. Current evidence (e.g., AlphaFold prediction) does not rule out indirect mechanisms (e.g., TEAD-mediated interactions), necessitating further biochemical proof.

4. The use of 92.1 cells (primary tumor-derived, BAP1 wild-type) to model metastatic uveal melanoma is problematic. Metastatic disease, often BAP1-mutant and liver-localized, represents the key clinical challenge. The chosen model limits translational relevance to metastasis prevention/treatment.

Minor Comments
1. Clarify the methodology for generating HEK293A Gq(Q209L) knock-in cells (e.g., CRISPR, site-directed mutagenesis).
2. Provide details for the CTGF luciferase reporter plasmid (e.g., promoter region, response elements) and explain how p300-mediated histone modifications influence plasmid reporter activity.
3. Strengthen evidence for direct YAP-p300 interaction (e.g., in vitro binding assays beyond AlphaFold predictions).
4. In Figure 3a, label blots with antibody names (e.g., “HA,” “Flag”) instead of “HA-p300” or “Flag-YAP” for clarity. The same for other related figures.
5. The proximity ligation assay (PLA) signal in Figure 3b appears weak.
6. Explain the omission of CYR61 in Figure 2f, given its prominence in other figures.
7. Extend mechanistic analyses to TAZ, which also contains a WW domain and may share p300-dependent regulation.
8. Validate YAP-K13 as the sole acetylation site (e.g., mass spectrometry) and assess genome-wide transcriptional changes in YAP-K13R or p300-KD models, instead of changes of a few signatures.
9. Use genetic p300 knockdown/knockout (not just inhibitors) to confirm its role in YAP acetylation and exclude off-target drug effects.
10. In Figure 6 experiments, include a “shRNA negative + DOX” control group to account for DOX-induced artifacts.
11. Address grammatical errors and improve clarity (e.g., awkward phrasing in Results/Discussion).

Reviewer #3

(Remarks to the Author)
The manuscript by Zhang et al reports the identification of p300 as a potential therapeutic target for GNAQ-driven uveal melanoma. CRIPSR screens identify p300 as the top hit synthetically lethal with GNAQ mutation. Mechanistically, p300 directly interacts with YAP/TAZ and colocalizes with YAP on chromatin genome-wide. P300 promotes YAP target gene expression primarily through acetylating histones, while YAP protein acetylation is dispensable for YAP transcriptional activity. Finally, mouse xenograft studies demonstrate that p300 inhibition suppress GNAQ-mutant uveal melanoma growth and metastasis in vivo. Overall, this is a solid study uncovering a novel therapeutic target specific for GNAQ-driven uveal melanoma. Methods are sound and conclusions are largely supported by experimental evidence.

A main question I have is the specificity of p300 vs CBP. P300 and CBP are close paralogues that function to compensate each other in most cell lines. In general, p300 becomes an essential gene only when CBP is lost or mutated. It is interesting that in GNAQ-mutant uveal melanoma cells, the authors observed global reduction in H3K27ac upon p300 single knockdown, suggesting that CBP fails to compensate p300 loss in these cells. It would be important to determine, however, the functional difference between p300 and CBP in GNAQ-mutant uveal melanoma. For instance, does CBP also interacts with Yap/TAZ through its KIX domain? What happens to Hippo pathway gene expression and global H3K27ac when CBP is depleted? Since CBP is not essential in GNAQ-mutant uveal melanoma cells, is CBP expressed at much lower levels than p300? And in patients, what is the CBP mutation status in general in GNAQ-mutant uveal melanoma vs non-mutant UM or BRAF-mutant cutaneous melanoma?

Minor points:
• Graphic abstract: "GNAQ/YAP NO" should be "GNAQ/YAP ON".
• Line 391 – "Fig. 2a-b" should be "Supplementary Fig. 2a-b"?
• Fig. 5, it seems YAP ChIP-seq was conducted in shp300 cells, it would be helpful to show it in heatmap as Fig. 5d and 5e.

Reviewer #4

(Remarks to the Author)
I co-reviewed this manuscript with one of the reviewers who provided the listed reports. This is part of the Nature Communications initiative to facilitate training in peer review and to provide appropriate recognition for Early Career Researchers who co-review manuscripts.

Version 1:

Reviewer comments:

Reviewer #1

(Remarks to the Author)
The authors have robustly responded to all my concerns. The authors are to be commended for such an extensive and conscientious rebuttal to all reviewer comments.

Reviewer #2

(Remarks to the Author)
While the authors have made some improvements, several important issues raised in the previous round have not been adequately addressed, and new concerns have emerged. I therefore recommend further revision before the paper can be considered for publication in Nature Communications.

My specific comments are as follows:

1. The authors highlight in the introduction that direct Gαq inhibitors (e.g., FR900359, YM-254890) cannot differentiate between wild-type and mutant Gαq, leading to unacceptable toxicity. This raises the question: how might BET or p300 inhibitors be employed to selectively target Gαq-mutant uveal melanoma cells over wild-type Gαq-expressing cells, thus distinguishing efficacy from toxicity?

2. “However, accumulating evidence indicates that these downstream branches do not contribute equally to UM maintenance.” The meaning of this statement is unclear, and it lacks a supporting reference.

3. Supplementary Fig. 7a should include an immunoblot for anti-panTEAD on the IP samples. Furthermore, although p300 can directly interact with YAP, it remains unclear why this interaction would be important than other YAP independent function of p300. I believe domain-specific functional assays should be involved to address this.

4. A previous review comment asked the authors to "explain how p300-mediated histone modifications influence plasmid reporter activity." I find that the authors neither adequately addressed nor correctly understood this comment. According to their proposed mechanism, p300 promotes YAP transcriptional activity via H3K27ac-dependent epigenetic modifications (Supplementary Fig. 4g). However, a transfected CTGF promoter-luciferase plasmid lacks histone-organized genomic DNA and should be insensitive to epigenetic regulation. Nonetheless, p300 knockdown reduced this reporter activity. The authors need to explain this discrepancy and clarify how their data support the original comment.

Given the above concerns, I believe the manuscript requires substantial additional experimentation and/or rewriting to clarify these points. I look forward to seeing a revised version that addresses these issues.

Reviewer #3

(Remarks to the Author)
The authors have adequately addressed my questions. However, given the added results, the title should be changed to "Synthetic lethal screens reveal p300/CBP as a targetable vulnerability for GNAQ-driven uveal melanoma" to better reflect the findings.

Reviewer #4

(Remarks to the Author)
I co-reviewed this manuscript with one of the reviewers who provided the listed reports. This is part of the Nature Communications initiative to facilitate training in peer review and to provide appropriate recognition for Early Career Researchers who co-review manuscripts.

Version 2:

Reviewer comments:

Reviewer #2

(Remarks to the Author)
The authors have adequately addressed all of my previous comments and concerns, and I have no further issues to raise.

Open Access This Peer Review File is licensed under a Creative Commons Attribution 4.0 International License, which permits use, sharing, adaptation, distribution and reproduction in any medium or format, as long as you give appropriate credit to the original author(s) and the source, provide a link to the Creative Commons license, and indicate if changes were made.
In cases where reviewers are anonymous, credit should be given to 'Anonymous Referee' and the source.
The images or other third party material in this Peer Review File are included in the article's Creative Commons license, unless indicated otherwise in a credit line to the material. If material is not included in the article's Creative Commons license and your intended use is not permitted by statutory regulation or exceeds the permitted use, you will need to obtain permission directly from the copyright holder.
To view a copy of this license, visit https://creativecommons.org/licenses/by/4.0/

Point-by-point response to the reviewers' comments:

REVIEWER COMMENTS

Reviewer #1 (Remarks to the Author):

Zhang et al describe an effort to identify genetic vulnerabilities of uveal melanoma (UM) characterized by oncogenic mutations in GNAQ/GNA11. While much of the work is rigorous, well-controlled, and shows material effect sizes, there is considerable confusion about the overall flow and consistency of the paper.

In the first story, CRISPR screens targeting a small set of epigenetic regulators in isogenic (Gaq-mutant) cells identify EP300 as highly essential specifically in Gaq-mutant cells. One potential issue is that the authors use a non-UM cell line, HEK293A, with a Gaq knock in to stand in for their tumor cells of choice. This seems to have been overcome by the considerable amount of validation work applied. Notably, 9 cell lines were subjected in vitro to three inhibitors of p300, encoded by the EP300 gene, showing consistent increased sensitivity in the GNAQ backgrounds. Then we fast forward to Figure 6, which demonstrates GNAQ-dependent vulnerability to EP300 knockdown by dox-inducible shRNA, and Figure 7, which shows the same dox-inducible knockdown also decreases hepatic metastasis after splenic injection. A p300 inhibitor is also shown to be effective in significantly reducing tumor growth rate after intrasplenic injection. This is a nice coherent body of work with strong results.

In between, Figure 2 diverts to a p300/YAP interaction study, with Figure 3 characterizing p300 effect on YAP transcriptional activity, Fig 4 showing p300 inhibitors also suppress targets of YAP transcription, and Fig 5 describing overlapping ChIP-seq of YAP and p300. Although this work is fairly comprehensive, the YAP/p300 transcriptional regulatory complex has been previously described (technically a YAP–TEAD–Smad–p300 complex; Fujii JXMed 2012; Hiemer JBC 2014) and neither the novelty nor the relevance seem to put this sub-story into this larger study of Gαq-dependent genetic vulnerabilities.

In short, while the work appears to all be solid, with well-controlled experiments and robust

results, the flow of the manuscript is a bit unwieldy. This reviewer understands the pressure to provide mechanism for discoveries of genetic vulnerability, but the EP300/YAP story doesn't mechanistically explain the vulnerability. This would be a perfectly legitimate and novel story with just Figures 1, 6, and 7.

Response:

(1) We thank the reviewer for raising the concern regarding our use of the HEK293A Gαq(Q209P) knock-in system as the discovery model. We agree that this system is not a lineage-faithful model of uveal melanoma. This limitation was addressed by extensive downstream validation in *GNAQ*-mutant UM models. Our intention in using this system was not to recapitulate the full biology of UM, but to establish a tractable isogenic discovery platform in which the principal engineered variable is oncogenic Gαq signaling. Importantly, the relevance of p300 to *GNAQ*-driven UM was not inferred from the HEK293A system alone, but was subsequently validated in multiple bona fide UM models in *vitro* and *in vivo*.

An isogenic system was selected because pooled CRISPR/Cas9 synthetic lethal screening is highly sensitive to genetic background. Comparing multiple *GNAQ*-mutant and *GNAQ*-wild-type UM cell lines would introduce substantial confounding from unrelated genomic differences, and the number of available *GNAQ*-wild-type UM cell lines is limited. In addition, these wild-type UM lines are themselves established melanoma cell lines and may harbor additional genetic alterations that confound the attribution of candidate dependencies specifically to *GNAQ* status. Accordingly, we considered an isogenic system more appropriate for primary discovery.

We also considered more lineage-matched isogenic strategies, but these are not readily compatible with large-scale pooled screening. One possible approach would be to generate an allele-corrected counterpart by editing the mutant *GNAQ* allele back to wild-type in *GNAQ*-mutant UM cells. However, because *GNAQ*-mutant UM cells depend on oncogenic Gαq signaling for sustained growth (**Yu et al., 2014; Yu et al., 2022**), stable establishment of such allele-corrected matched counterparts is impractical for pooled library screening. Another possible approach would be to introduce mutant *GNAQ* into normal uveal melanocytes. However, standard nonmalignant reference systems in the UM field remain limited and heterogeneous, and published studies have therefore used substitute controls such as PIG1 human epidermal melanocytes (**Gu et al., 2023**), Melan-a murine epidermal melanocytes (**Van Raamsdonk et al., 2009; Yu et al., 2014**), or ARPE-19 retinal pigment epithelial cells

(**Jin et al., 2018**), each of which captures only part of the relevant biological context. Primary cultured uveal melanocytes are more physiologically relevant, but their limited expansion capacity and culture stability make them poorly suited for pooled lentiviral CRISPR/Cas9 library screening. Reported immortalized human uveal melanocyte systems also involve immortalization manipulations (**Meng et al., 2022**) and, to our knowledge, have not become widely adopted platforms for large-scale genetic screening. We have revised the Discussion (**Discussion Section, lines 1011-1016**) to better explain these considerations.

The revised Discussion section (lines 1011-1016) now reads as follows:

"We also note that the HEK293A Gαq(Q209P) knock-in system used for the discovery screen is not a lineage-faithful model of UM. Because suitable lineage-faithful isogenic models are currently not available for pooled genetic screening, we used this system as a tractable discovery platform; future studies using uveal melanocyte-derived isogenic Gαq-wild-type and Gαq-mutant models may provide a more physiologically relevant framework."

To address the reviewer's concern regarding biological relevance, we have added several lines of evidence showing that the HEK293A Gαq(Q209P) knock-in model captures key consequences of oncogenic Gαq signaling. In our previous study, this knock-in clone was functionally characterized and shown to display constitutive YAP activation, consistent with canonical downstream signaling of mutant Gαq (**Zhang et al., 2022**). In the revised manuscript, we further show that Gαq(Q209P) knock-in induces extensive transcriptomic rewiring and global changes in chromatin accessibility, with concordant changes in accessibility around transcription start sites of differentially expressed genes (**Supplementary Fig. 1a-c**). Functionally, *GNAQ*-mutant HEK293A cells exhibited enhanced anchorage-independent growth (**Supplementary Fig. 1d**), and inducible knockdown of Gαq selectively suppressed colony formation in *GNAQ*-mutant cells but had minimal effect in wild-type cells (**Supplementary Fig. 1e**). Together, these results indicate that the knock-in mutation induces coordinated signaling, epigenetic, and growth changes, and renders the cells selectively dependent on oncogenic Gαq signaling.

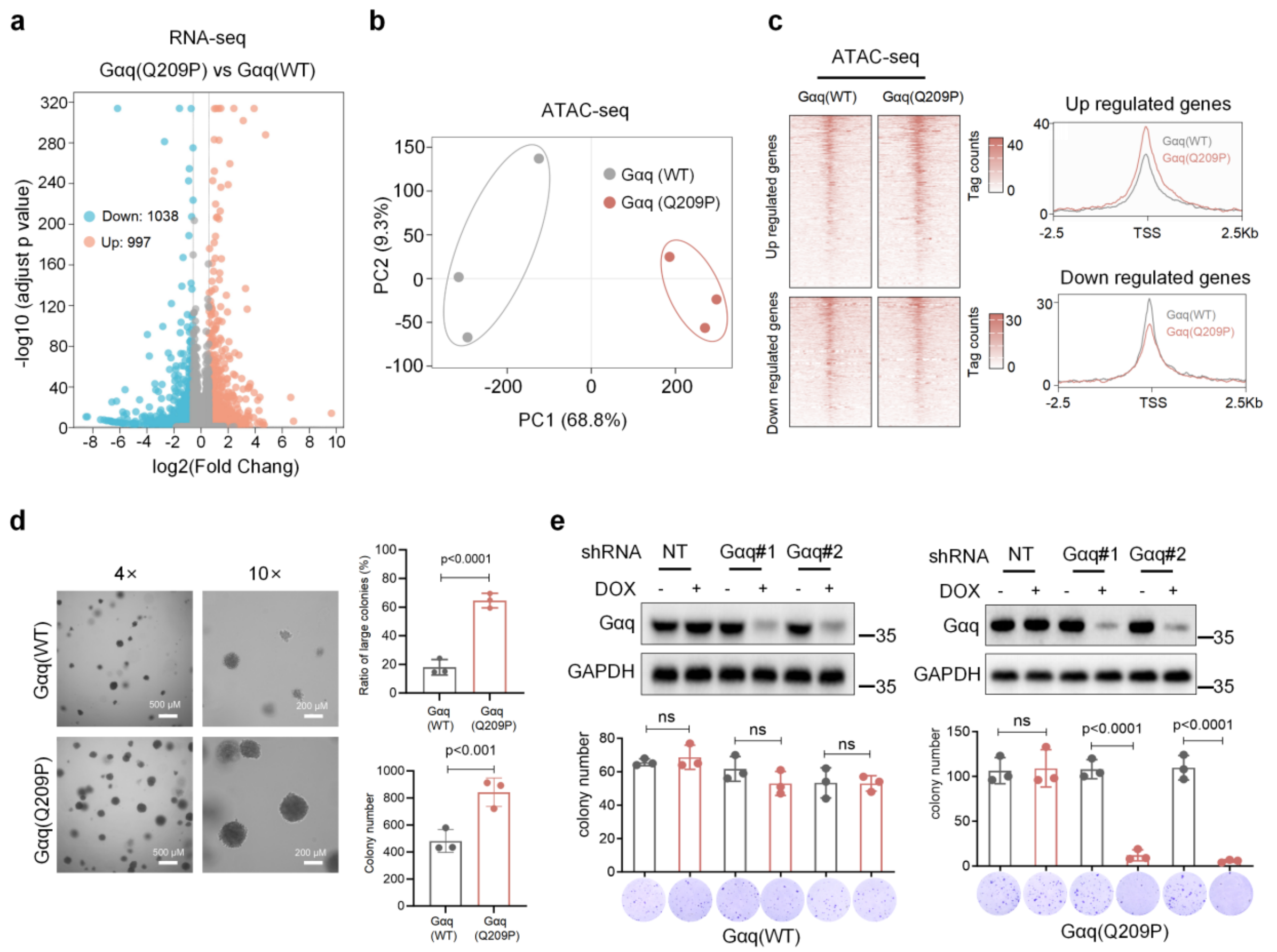

Supplementary Fig. 1: Gαq(Q209P) knock-in induces transcriptomic rewiring and global alterations in chromatin accessibility.

a, Volcano plot showing transcriptional changes in Gαq(Q209P)-mutant HEK293A cells relative to wild-type cells. A total of 997 genes were significantly upregulated and 1,038 genes were significantly downregulated, as defined in the Methods. RNA-seq was performed with 3 biological replicates. **b**, Principal component analysis of ATAC-seq profiles in Gαq(Q209P)-mutant and wild-type HEK293A cells. Biological replicates cluster according to genotype, indicating global differences in chromatin accessibility. ATAC-seq was performed with 3 biological replicates. **c**, Integration of RNA-seq and ATAC-seq datasets. Heatmaps (left) and average accessibility profiles (right) show chromatin accessibility around transcription start sites (TSSs; ± 2.5 kb) of genes significantly upregulated (n = 997) or downregulated (n = 1,038) in Gαq(Q209P)-mutant HEK293A cells. Upregulated genes show increased accessibility around their TSSs, whereas downregulated genes show reduced accessibility. **d**, Soft-agar assays comparing anchorage-independent growth of Gαq(Q209P)-mutant and wild-type HEK293A cells. Colonies with diameters >100 μm were scored as positive, and the fraction of colonies ≥ 200 μm was quantified (>1,000 colonies counted per sample). Data are presented as mean ± SD (n = 3 biological replicates) from one representative experiment out of three. Statistical significance was assessed by two-sided Student's t-test. **e**, Immunoblot analysis of Gαq in Gαq(Q209P)-mutant HEK293A cells expressing doxycycline-inducible non-targeting (NT) or Gαq-targeting shRNAs, treated with or without doxycycline (200 ng/ml). Colony-formation assays show that inducible Gαq knockdown selectively suppresses growth of Gαq(Q209P)-mutant cells. Data are presented

as mean ± SD (n = 3 biological replicates) from one representative experiment out of three. Statistical significance was determined by two-sided Student's t-test; ns, not significant.

To further assess disease relevance, we compared the transcriptional program induced by Gαq(Q209P) knock-in in HEK293A cells with the TCGA UM cohort using a framework adapted from a prior study of Gαq-driven UM, in which Gαq-negative skin cutaneous melanoma (SKCM) samples were used as the reference control because of the limited number of Gαq-negative UM cases (**Feng et al., 2019**). Using this approach, we found a highly significant overlap between genes upregulated in *GNAQ*-mutant HEK293A cells and genes overexpressed in Gαq-positive human UM, defined as tumors harboring mutation or amplification in *GNAQ*, *GNA11*, or *CYSLTR2* (hypergeometric test, $P < 3.598 \times 10$^-39; see Methods). We do not interpret this as evidence that HEK293A cells fully model UM biology; rather, it supports the conclusion that this isogenic system captures a substantial subset of transcriptional features associated with aberrant Gαq signaling in human UM samples.

Importantly, the HEK293A Gαq(Q209P) system was used to identify candidate vulnerabilities associated with oncogenic Gαq signaling, rather than as a stand-alone disease model. The relevance of p300 to *GNAQ*-driven uveal melanoma was established through subsequent validation in multiple UM models *in vitro* and *in vivo*, rather than inferred from the HEK293A system alone. We have revised the manuscript to make this distinction more explicit.

Taken together, although the HEK293A Gαq(Q209P) knock-in model does not capture the full lineage-specific biology of UM, it provides a practical and mechanistically informative isogenic platform for identifying candidate vulnerabilities associated with oncogenic Gαq signaling, with subsequent validation in bona fide UM models.

(2) We thank the reviewer for this thoughtful comment regarding the overall flow of the manuscript. We agree that, in the original version, the rationale linking the identification of *EP300* as a *GNAQ*-associated vulnerability to the subsequent mechanistic analyses was not presented with sufficient clarity. To address this concern, we revised both the Introduction and the Results to make this logic more explicit.

In the revised Introduction (**Introduction Section, lines 71-87**), we now clarify that although oncogenic Gαq can engage multiple downstream signaling branches, prior studies in uveal melanoma have established YAP as a key downstream effector of oncogenic Gαq signaling and an important mediator of tumor maintenance (**Yu et al., 2014; Feng et al., 2014; Feng et al., 2019**). We further note that both direct targeting of mutant Gαq and direct pharmacological inhibition of YAP remain challenging, which provides the rationale for seeking druggable regulators that support the Gαq-YAP signaling axis in Gαq-mutant UM.

The revised Introduction section (lines 71-87) now reads as follows:

"Oncogenic Gαq can engage multiple downstream signaling branches, including the canonical PLCβ-PKC-MAPK cascade and YAP signaling [11]. However, accumulating evidence indicates that these downstream branches do not contribute equally to UM maintenance. Although MAPK pathway activation has been documented in UM, clinical studies of MEK inhibition have shown limited benefit, suggesting that this pathway may not represent the dominant therapeutic dependency in Gαq-driven UM [12]. In contrast, prior studies in UM have established that oncogenic Gαq activates YAP and that YAP is required for Gαq-driven tumorigenesis and tumor maintenance [13, 14, 15]. These findings support a model in which, although oncogenic Gαq activates multiple downstream pathways, YAP functions as a major downstream transcriptional effector in UM. However, YAP lacks enzymatic activity and is generally considered difficult to target directly because it does not contain a suitable small-molecule-binding pocket. Currently, no specific YAP inhibitors are in clinical use [16]. Although YAP-targeting approaches such as antisense RNA are under clinical investigation (NCT04659096), these strategies still face delivery challenges compared with conventional small-molecule therapeutics. Therefore, identifying druggable regulators of this critical downstream signaling axis may provide a more tractable therapeutic strategy for Gαq-mutant UM."

In the revised Results (**Results Section, lines 526-533**), we now also explain more clearly why the study proceeds from *EP300* vulnerability to YAP-focused mechanistic analysis. Specifically, after identifying *EP300* as a top synthetic lethal candidate, we performed RNA-seq following p300 depletion in 92.1 cells and found that the Hippo pathway was the most significantly enriched pathway among downregulated genes. Because YAP is a major transcriptional coactivator downstream of Hippo signaling and has been established in UM as

a key transcriptional effector downstream of oncogenic Gαq, we then investigated whether p300 contributes to YAP-dependent transcription in *GNAQ*-mutant UM cells.

The revised Results section (lines 526-533) now reads as follows:
"To gain mechanistic insight into the synthetic lethality between *EP300* and oncogenic *GNAQ*, we first examined the transcriptional changes induced by p300 depletion in 92.1 cells, a *GNAQ*-mutant UM cell line, by RNA sequencing (RNA-seq). KEGG enrichment analysis of the downregulated genes identified the Hippo pathway as the most significantly enriched pathway (Supplementary Fig. 4a, b). YAP is a major transcriptional coactivator downstream of Hippo signaling, and previous studies in UM have established YAP as a key transcriptional effector downstream of oncogenic Gαq[13, 14, 15]. We therefore investigated whether p300 contributes to YAP-dependent transcription in *GNAQ*-mutant UM cells."

Together, these revisions were made specifically to clarify the conceptual flow of the study and to explain why the mechanistic analyses of p300 and YAP are positioned as a direct mechanistic follow-up to the identification of *EP300* as a *GNAQ*-associated vulnerability in UM.

Related to this flow issue, we further clarify that the p300/YAP mechanistic analyses were not intended as a separate sub-story, but rather as a mechanistic follow-up to the identification of *EP300* as a vulnerability in *GNAQ*-mutant UM. After identifying *EP300* as a top synthetic lethal candidate, we performed RNA-seq following p300 depletion in *GNAQ*-mutant UM cells to investigate the transcriptional consequences of p300 loss. KEGG enrichment analysis of the downregulated genes identified the Hippo pathway as the most significantly enriched pathway. In the context of UM, where prior studies have established YAP as a key transcriptional effector downstream of oncogenic Gαq, this result led us to examine whether p300 contributes to YAP-dependent transcription. Thus, the mechanistic analyses were included to understand how p300 is functionally linked to a central downstream oncogenic axis in *GNAQ*-mutant UM, rather than to introduce an unrelated YAP/p300 story.

(3) We appreciate the reviewer's point that prior studies have implicated YAP/TAZ-associated transcriptional complexes involving TEAD, SMADs and p300/CBP in other

biological contexts. We have therefore revised the Discussion (**Discussion Section, lines 917-926**) to discuss these studies more explicitly and to better delineate what the present work adds in the context of Gαq-mutant UM.

The revised Discussion section (lines 917-926) now reads as follows:

"p300 and CBP are closely related paralogous transcriptional coactivators that regulate gene expression and chromatin state, and both have been implicated in sustaining malignant transcriptional programs [33, 34]. Prior studies, largely based on co-immunoprecipitation, have suggested that YAP forms complexes with p300/CBP in other biological contexts [28, 35, 36]. However, how p300/CBP coordinate the transcriptional activity of constitutively activated YAP downstream of oncogenic Gαq in UM remains unclear. Our data show that p300 and YAP interact in the nucleus and further demonstrate direct binding between the KIX domain of p300 and the WW1/2 domains of YAP. A similar WW-KIX-dependent interaction was also observed for TAZ, suggesting that both YAP and TAZ may be regulated by p300/CBP through a shared domain-dependent interaction."

First, in the revised manuscript, we further strengthened the mechanistic analysis of the p300–YAP interaction itself. Specifically, in response to reviewer comments, we added SPR assays and TEAD-dependence testing, which showed direct, domain-dependent binding between the KIX domain of p300 and the WW1/2 region of YAP and argued against a TEAD-mediated bridging mechanism (**Supplementary Fig. 7**).

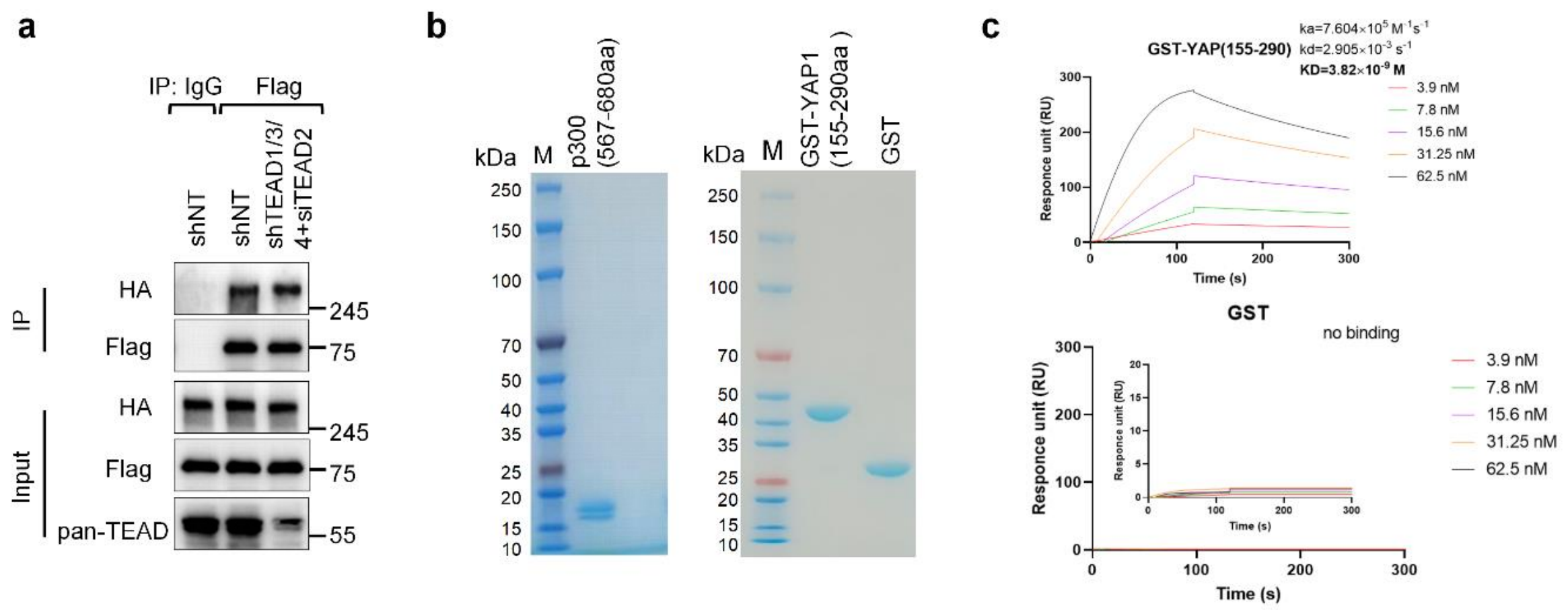

Supplementary Fig. 7: YAP directly binds p300 in a TEAD-independent manner.

a, TEAD knockdown does not impair the interaction between YAP and p300. HEK293T cells stably expressing shRNAs targeting TEAD1, 3, and 4 were transfected with TEAD2 siRNA together with YAP and p300 constructs. Cells were harvested 48 h after transfection and subjected to co-immunoprecipitation followed by immunoblotting with the indicated antibodies. Cells expressing non-targeting shRNA (shNT)

served as controls. **b**, SDS–PAGE analysis of purified recombinant proteins used for surface plasmon resonance (SPR) assays. c, SPR analysis of the interaction between recombinant p300 (567-680 aa) and GST-YAP (155-290 aa). p300 bound GST-YAP in a concentration-dependent manner, whereas no binding was detected with GST alone.

More importantly, in response to Reviewer #3, we added new CBP analyses in the revised manuscript. Because *CREBBP* also ranked among the top negatively selected genes in our screen, these additional experiments allowed us to address how p300 and CBP function in relation to YAP-associated transcription in *GNAQ*-mutant UM. We found that CBP, like p300, interacts with YAP through its KIX domain, and that CBP knockdown produced effects similar to those of p300 knockdown in *GNAQ*-mutant UM cells, including reduced global H3K27ac, decreased H3K27ac at YAP target promoters, suppression of YAP-associated transcriptional output, and impaired clonogenic growth. In addition, co-depletion of CBP and p300 was not more inhibitory than depletion of either paralogue alone, and UM TCGA analysis did not support a CBP-deficient background. Together, based on these findings, we propose a working model in which both CBP and p300 sustain the YAP-associated transcriptional program in *GNAQ*-mutant UM cells.

We believe this revised p300/CBP-YAP framework is an important advance beyond the prior literature, because it not only clarifies the functional relationship between the two paralogues in this disease context but also has therapeutic implications. Specifically, our data suggest that inhibition of either p300 or CBP alone may be sufficient to exert anti-tumor effects and could still offer an expanded therapeutic index in Gαq-mutant UM. Because *EP300* emerged as the top-ranked hit in our screen and p300 was the paralogue validated most extensively *in vivo*, p300-directed strategies provide a practical example for therapeutic development. We have revised the Discussion accordingly (**Discussion Section, lines 979-1005**).

The revised Discussion section (lines 979-1005) now reads as follows:

"Dependency on p300 and CBP appears to be context-dependent across cancers. In some tumor types, p300 dependency arises in the setting of CBP deficiency, consistent with a paralog addiction model [44, 45]. In other contexts, however, tumor cells may remain dependent on both p300 and CBP [46]. Our findings in Gαq-mutant UM instead support the latter possibility. In UM cells, depletion of either p300 or CBP impaired clonogenic growth, and co-depletion was not more inhibitory than depletion of either paralogue alone. *In vivo*, our

study focused on p300, and p300 depletion was sufficient to induce robust anti-growth and anti-metastatic effects. Analysis of the UM TCGA cohort further showed that *CREBBP* expression was not lower than *EP300*, and *CREBBP* mutations were not observed (Supplementary Fig. 16), arguing against a CBP-deficient background in this disease. Consistent with functional involvement of both paralogues, *EP300* and *CREBBP* mRNA levels positively correlated with the YAP target genes *CYR61* and *CTGF* in the UM TCGA cohort (Supplementary Fig. 17). Together, these observations suggest that dependency on p300 or CBP in Gαq-mutant UM is unlikely to be explained by loss of the other paralogue. Evidence from knockout studies in the mouse has shown that CBP and p300 are critical for the development and function of normal cells and tissues [47, 48]. Therefore, inhibition of either p300 or CBP alone may be sufficient to exert anti-tumor effects and could still offer an expanded therapeutic index in Gαq-mutant UM. Because *EP300* emerged as the top-ranked hit in our screen and p300 was the paralogue validated most extensively *in vivo* in this study, p300-directed strategies provide a practical example for therapeutic development. While current HAT and BRD small molecule inhibitors cannot discriminate between CBP and p300, recently developed small-molecule and peptide proteolysis-targeting chimera (PROTAC) drugs demonstrate potent and selective p300 degradation [49, 50]. This highlights the potential of p300-targeting PROTACs as a promising therapeutic approach. Additionally, we propose that new therapeutics may be designed around the YAP-p300 interaction, particularly by targeting the p300 KIX domain. Interestingly, there has been growing interest in investigating KIX domain inhibitors, and several small molecule KIX inhibitors have been reported [51, 52, 53]."

References:

Yu F, et al. Mutant Gq/11 promote uveal melanoma tumorigenesis by activating YAP. Cancer Cell, 2014, 25(6):822-30.

Feng X, et al. Hippo-independent activation of YAP by the GNAQ uveal melanoma oncogene through a trio-regulated rho GTPase signaling circuitry. Cancer Cell, 2014, 25(6):831-45.

Feng X, et al. A platform of synthetic lethal gene interaction networks reveals that the GNAQ uveal melanoma oncogene controls the Hippo pathway through FAK. Cancer Cell, 2019, 35(3): 457-72.

Yu L, et al. Co-occurrence of BAP1 and SF3B1 mutations in uveal melanoma induces cellular senescence. Mol Oncol, 2022, 16(3):607-29.

Gu X, et al. Epigenetic drug library screening reveals targeting DOT1L abrogates NAD+ synthesis by reprogramming H3K79 methylation in uveal melanoma. J Pharm Anal, 2023, 13(1):24-38.
Van Raamsdonk CD, et al. Frequent somatic mutations of GNAQ in uveal melanoma and blue naevi. Nature, 2009, 457(7229):599-602.
Jin Y, et al. Neddylation blockade diminishes hepatic metastasis by dampening cancer stem-like cells and angiogenesis in uveal melanoma. 2018, Clin Cancer Res, 24(15):3741-54.
Meng F, et al. SUMOylation regulates Rb hyperphosphorylation and inactivation in uveal melanoma. Cancer Sci, 2022, 113(2):622-33.
Zhang G, et al. Reciprocal positive regulation between BRD4 and YAP in GNAQ-mutant uveal melanoma cells confers sensitivity to BET inhibitors. Pharmacol Res, 2022, 184:106464.

Reviewer #2 (Remarks to the Author):

This study aims to identify targetable vulnerabilities for uveal melanoma through a functional genomic screen focused on epigenetic regulators. The authors propose p300 as a therapeutic target, supported by mechanistic evidence suggesting that p300 inhibition suppresses YAP/TAZ-dependent genes via direct interaction between YAP's WW domain and p300's KIX domain. While the work highlights p300's potential in treating uveal melanoma, critical evidence gaps weaken support for the central conclusions.

Major Comments

1. The observed epigenetic rewiring in HEK293A cells with Gq(Q209L) knock-in (generation process should be detailed in Methods) is intriguing. However, the relevance of this model to uveal melanoma cell transformation remains unclear. The study does not convincingly establish p300 as a synthetic lethal partner in GNAQ-driven uveal melanoma or address how this switch reflects actual tumor biology.

Response:

We thank the reviewer for this important comment.

(1) We have expanded the Methods section to provide a more detailed description of the generation of the Gαq(Q209P) knock-in HEK293A cells. Specifically, we clarified that both the sgRNA and the donor template used for homology-directed repair were designed using

Benchling, and we described the CRISPR/Cas9-mediated editing strategy, clonal isolation, and validation by PCR and Sanger sequencing. We also noted that the selected knock-in clone used in this study had been functionally characterized previously, including activation of YAP signaling downstream of mutant Gαq (**Zhang et al., 2022**).

(2) We agree that the HEK293A Gαq(Q209P) knock-in system is not a lineage-faithful model of uveal melanoma (UM). We note that this issue was also raised by Reviewer #1. Our intention in using this model was not to recapitulate the full biology of UM, but to establish a tractable, isogenic, and screening-compatible discovery platform in which the principal engineered variable is oncogenic Gαq signaling. We have revised the manuscript to clarify this rationale, to better define the limitations of the model, and to further support its relevance for identifying Gαq-linked vulnerabilities.

An isogenic system was selected because pooled CRISPR/Cas9 synthetic lethal screening is highly sensitive to genetic background. Comparing multiple *GNAQ*-mutant and *GNAQ*-wild-type UM cell lines would introduce substantial confounding from unrelated genomic differences, and the number of available *GNAQ*-wild-type UM cell lines is limited. In addition, these wild-type UM lines are themselves established melanoma cell lines and may harbor additional genetic alterations that confound the attribution of candidate dependencies specifically to *GNAQ* status. Accordingly, we considered an isogenic system more appropriate for primary discovery.

We also considered more lineage-matched isogenic strategies, but these are not readily compatible with large-scale pooled screening. One possible approach would be to generate an allele-corrected counterpart by editing the mutant *GNAQ* allele back to wild-type in *GNAQ*-mutant UM cells. However, because *GNAQ*-mutant UM cells depend on oncogenic Gαq signaling for sustained growth (**Yu et al., 2014; Yu et al., 2022**), stable establishment of such allele-corrected matched counterparts is impractical for pooled library screening. Another possible approach would be to introduce mutant *GNAQ* into normal uveal melanocytes. However, standard nonmalignant reference systems in the UM field remain limited and heterogeneous, and published studies have therefore used substitute controls such as PIG1 human epidermal melanocytes (**Gu et al., 2023**), Melan-a murine epidermal melanocytes (**Van Raamsdonk et al., 2009; Yu et al., 2014**), or ARPE-19 retinal pigment epithelial cells (**Jin et al., 2018**), each of which captures only part of the relevant biological context. Primary

cultured uveal melanocytes are more physiologically relevant, but their limited expansion capacity and culture stability make them poorly suited for pooled lentiviral CRISPR/Cas9 library screening. Reported immortalized human uveal melanocyte systems also involve immortalization manipulations (**Meng et al., 2022**) and, to our knowledge, have not become widely adopted platforms for large-scale genetic screening. We have revised the Discussion (**Discussion Section, lines 1011-1016**) to better explain these considerations.

The revised Discussion section (lines 1011-1016) now reads as follows:

"We also note that the HEK293A Gαq(Q209P) knock-in system used for the discovery screen is not a lineage-faithful model of UM. Because suitable lineage-faithful isogenic models are currently not available for pooled genetic screening, we used this system as a tractable discovery platform; future studies using uveal melanocyte-derived isogenic Gαq-wild-type and Gαq-mutant models may provide a more physiologically relevant framework."

To address the reviewer's concern regarding biological relevance, we have added several lines of evidence showing that the HEK293A Gαq(Q209P) knock-in model captures key consequences of oncogenic Gαq signaling. In our previous study, this knock-in clone was functionally characterized and shown to display constitutive YAP activation, consistent with canonical downstream signaling of mutant Gαq (**Zhang et al., 2022**). In the revised manuscript, we further show that Gαq(Q209P) knock-in induces extensive transcriptomic rewiring and global changes in chromatin accessibility, with concordant changes in accessibility around transcription start sites of differentially expressed genes (**Supplementary Fig. 1a-c**). Functionally, GNAQ-mutant HEK293A cells exhibited enhanced anchorage-independent growth (**Supplementary Fig. 1d**), and inducible knockdown of Gαq selectively suppressed colony formation in GNAQ-mutant cells but had minimal effect in wild-type cells (**Supplementary Fig. 1e**). Together, these results indicate that the knock-in mutation induces coordinated signaling, epigenetic, and growth changes, and renders the cells selectively dependent on oncogenic Gαq signaling.

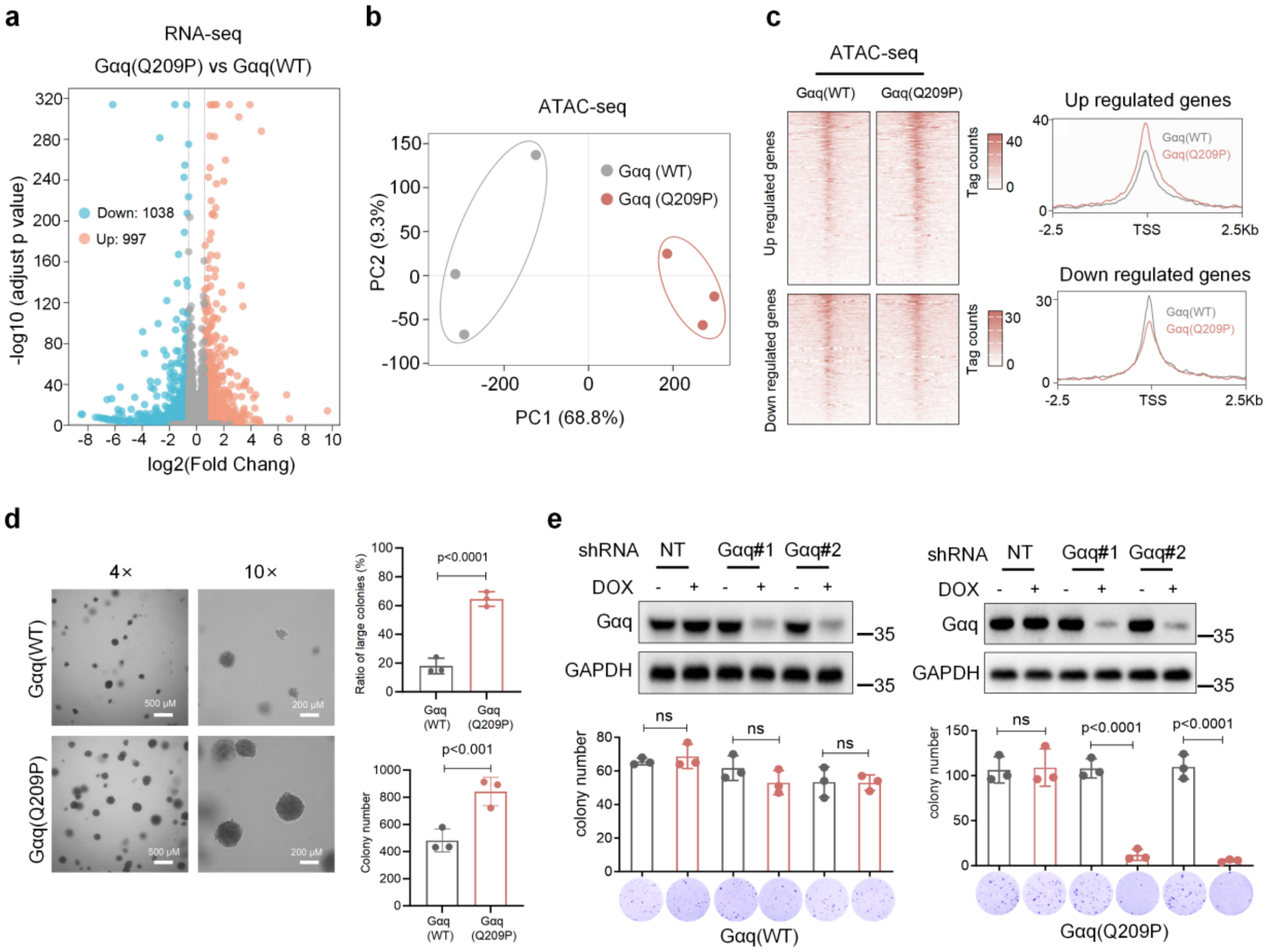

Supplementary Fig. 1: Gαq(Q209P) knock-in induces transcriptomic rewiring and global alterations in chromatin accessibility.

a, Volcano plot showing transcriptional changes in Gαq(Q209P)-mutant HEK293A cells relative to wild-type cells. A total of 997 genes were significantly upregulated and 1,038 genes were significantly downregulated, as defined in the Methods. RNA-seq was performed with 3 biological replicates. **b**, Principal component analysis of ATAC-seq profiles in Gαq(Q209P)-mutant and wild-type HEK293A cells. Biological replicates cluster according to genotype, indicating global differences in chromatin accessibility. ATAC-seq was performed with 3 biological replicates. **c**, Integration of RNA-seq and ATAC-seq datasets. Heatmaps (left) and average accessibility profiles (right) show chromatin accessibility around transcription start sites (TSSs; ± 2.5 kb) of genes significantly upregulated (n = 997) or downregulated (n = 1,038) in Gαq(Q209P)-mutant HEK293A cells. Upregulated genes show increased accessibility around their TSSs, whereas downregulated genes show reduced accessibility. **d**, Soft-agar assays comparing anchorage-independent growth of Gαq(Q209P)-mutant and wild-type HEK293A cells. Colonies with diameters >100 μm were scored as positive, and the fraction of colonies ≥ 200 μm was quantified (>1,000 colonies counted per sample). Data are presented as mean ± SD (n = 3 biological replicates) from one representative experiment out of three. Statistical significance was assessed by two-sided Student's t-test. **e**, Immunoblot analysis of Gαq in Gαq(Q209P)-mutant HEK293A cells expressing doxycycline-inducible non-targeting (NT) or Gαq-targeting shRNAs, treated with or without doxycycline (200 ng/ml). Colony-formation assays show that inducible Gαq knockdown selectively suppresses growth of Gαq(Q209P)-mutant cells. Data are presented

as mean ± SD (n = 3 biological replicates) from one representative experiment out of three. Statistical significance was determined by two-sided Student's t-test; ns, not significant.

To further assess disease relevance, we compared the transcriptional program induced by Gαq(Q209P) knock-in in HEK293A cells with the TCGA UM cohort using a framework adapted from a prior study of Gαq-driven UM, in which Gαq-negative skin cutaneous melanoma (SKCM) samples were used as the reference control because of the limited number of Gαq-negative UM cases (**Feng et al., 2019**). Using this approach, we found a highly significant overlap between genes upregulated in GNAQ-mutant HEK293A cells and genes overexpressed in Gαq-positive human UM, defined as tumors harboring mutation or amplification in *GNAQ*, *GNA11*, or *CYSLTR2* (hypergeometric test, $P < 3.598 \times 10^{-39}$; see Methods). We do not interpret this as evidence that HEK293A cells fully model UM biology; rather, it supports the conclusion that this isogenic system captures a substantial subset of transcriptional features associated with aberrant Gαq signaling in human UM samples.

Importantly, the HEK293A Gαq(Q209P) system was used to identify candidate vulnerabilities associated with oncogenic Gαq signaling, rather than as a stand-alone disease model. The relevance of p300 to *GNAQ*-driven uveal melanoma was established through subsequent validation in multiple UM models *in vitro* and *in vivo*, rather than inferred from the HEK293A system alone. We have revised the manuscript to make this distinction more explicit.

Taken together, although the HEK293A Gαq(Q209P) knock-in model does not capture the full lineage-specific biology of UM, it provides a practical and mechanistically informative isogenic platform for identifying candidate vulnerabilities associated with oncogenic Gαq signaling, with subsequent validation in bona fide UM models.

References:

Yu F, et al. Mutant Gq/11 promote uveal melanoma tumorigenesis by activating YAP. Cancer Cell, 2014, 25(6):822-30.

Yu L, et al. Co-occurrence of BAP1 and SF3B1 mutations in uveal melanoma induces cellular senescence. Mol Oncol, 2022, 16(3):607-29.

Gu X, et al. Epigenetic drug library screening reveals targeting DOT1L abrogates NAD+ synthesis by reprogramming H3K79 methylation in uveal melanoma. J Pharm Anal, 2023, 13(1):24-38.

Van Raamsdonk CD, et al. Frequent somatic mutations of GNAQ in uveal melanoma and blue naevi. Nature, 2009, 457(7229):599-602.
Jin Y, et al. Neddylation blockade diminishes hepatic metastasis by dampening cancer stem-like cells and angiogenesis in uveal melanoma. 2018, Clin Cancer Res, 24(15):3741-54.
Meng F, et al. SUMOylation regulates Rb hyperphosphorylation and inactivation in uveal melanoma. Cancer Sci, 2022, 113(2):622-33.
Zhang G, et al. Reciprocal positive regulation between BRD4 and YAP in GNAQ-mutant uveal melanoma cells confers sensitivity to BET inhibitors. Pharmacol Res, 2022, 184:106464.
Feng X, et al. A platform of synthetic lethal gene interaction networks reveals that GNAQ uveal melanoma oncogene controls the Hippo pathway through FAK. Cancer Cell, 2019, 35(3):457-72.

2. While focusing on epigenetic regulators is justified by the low mutation burden in uveal melanoma, the authors provide no direct evidence linking epigenetic mechanisms to disease pathogenesis. This omission undermines the rationale for restricting the screen to epigenetic targets.

Response:

We thank the reviewer for this important comment. We agree that low mutation burden alone does not provide direct evidence linking epigenetic mechanisms to UM pathogenesis and is not, by itself, sufficient to justify restricting the screen to epigenetic regulators. We have therefore revised the manuscript to clarify that the rationale for our focused library was not based solely on the relatively low mutation burden and nucleotide-level stability of UM.

Rather, our rationale was based on convergent evidence that oncogenic Gαq is associated with transcriptional dysregulation and chromatin remodeling. Patient-level analyses, including the TCGA-based framework reported by Feng et al., identified distinct transcriptional features associated with Gαq-driven UM. In our previous study, oncogenic Gαq mutations induced broad transcriptional changes and conferred enhanced sensitivity to transcription-associated inhibitors (**Zhang et al., 2022**). In the revised manuscript, we now provide additional evidence from our isogenic HEK293A Gαq(Q209P) system showing that oncogenic Gαq induces coordinated transcriptomic and chromatin remodeling, accompanied by oncogenic growth phenotypes and selective dependency on Gαq signaling. Consistent with this, transcriptional changes induced by Gαq(Q209P) in this isogenic system

significantly overlap with genes overexpressed in Gαq-positive human UM (**Supplementary Fig. 1; see also our response to Major Concern #1**).

Taken together, these observations suggest that epigenetic regulators, as modulators of transcriptional output and chromatin state, could provide a biologically informed source of vulnerabilities in Gαq-driven UM. Our use of a focused sgRNA library targeting epigenetic regulators should therefore not be interpreted to imply that epigenetic mechanisms are the sole drivers of UM pathogenesis. Rather, it reflects a hypothesis-guided prioritization of a mechanistically relevant and therapeutically tractable subset of targets for CRISPR/Cas9 screening.

To address this concern, we revised the relevant text in both the Introduction and the Results. In the revised Introduction, we now frame the focused screen in the context of the difficulty of directly targeting Gαq and YAP, together with prior evidence for Gαq-associated transcriptional dysregulation and sensitivity to perturbation of transcription-associated regulators (**Introduction section, lines 65-97**). In the revised Results, we now first show that Gαq(Q209P) knock-in induces transcriptional and chromatin rewiring in an isogenic system and then demonstrate that these changes significantly overlap with transcriptional features observed in human Gαq-driven UM, thereby supporting the rationale for focused screening of epigenetic regulators (**Results section, lines 418-461**).

The revised Introduction section (lines 65-97) now reads as follows:

"Mutations in *GNAQ/11* impair GTPase activity, leading to constitutive activation of G proteins in their GTP-bound state. Because Gαq proteins play essential roles in numerous physiological processes, direct targeting of Gαq remains unfeasible. Compounds such as FR900359 and YM-254890, which inhibit Gαq directly, do not distinguish between wild-type and mutant Gαq, resulting in toxicity and rendering them unsuitable for clinical use [9, 10]. Consequently, targeting the downstream signaling pathways of Gαq has emerged as a more viable therapeutic strategy. Oncogenic Gαq can engage multiple downstream signaling branches, including the canonical PLCβ-PKC-MAPK cascade and YAP signaling [11]. However, accumulating evidence indicates that these downstream branches do not contribute equally to UM maintenance. Although MAPK pathway activation has been documented in UM, clinical studies of MEK inhibition have shown limited benefit, suggesting that this

pathway may not represent the dominant therapeutic dependency in Gαq-driven UM [12]. In contrast, prior studies in UM have established that oncogenic Gαq activates YAP and that YAP is required for Gαq-driven tumorigenesis and tumor maintenance [13, 14, 15]. These findings support a model in which, although oncogenic Gαq activates multiple downstream pathways, YAP functions as a major downstream transcriptional effector in UM. However, YAP lacks enzymatic activity and is generally considered difficult to target directly because it does not contain a suitable small-molecule-binding pocket. Currently, no specific YAP inhibitors are in clinical use [16]. Although YAP-targeting approaches such as antisense RNA are under clinical investigation (NCT04659096), these strategies still face delivery challenges compared with conventional small-molecule therapeutics. Therefore, identifying druggable regulators of this critical downstream signaling axis may provide a more tractable therapeutic strategy for Gαq-mutant UM.

Guided by this rationale, we sought to identify therapeutically actionable vulnerabilities in Gαq-mutant UM. Prior patient-level and experimental studies suggest that Gαq-driven UM is associated with transcriptional dysregulation and increased sensitivity to perturbation of transcription-associated regulators [14, 17]. As key modulators of transcriptional output and chromatin state, epigenetic regulators were therefore prioritized for focused vulnerability screening. To identify such vulnerabilities, we performed parallel CRISPR/Cas9 screens in an isogenic pair of cells differing only in *GNAQ* status. This strategy identified the acetyltransferase p300 as a key vulnerability, which we subsequently validated using genetic and pharmacological approaches *in vitro* and *in vivo*, thereby revealing p300 as a potential therapeutic target for Gαq-mutant UM."

The revised Results section (lines 418-461) now reads as follows:

"Because oncogenic Gαq signaling is associated with distinct transcriptional features in UM[14], we asked whether Gαq(Q209P) knock-in could establish a coordinated transcriptional and chromatin state linked to exploitable vulnerabilities. To address this question, we performed RNA-seq and ATAC-seq analyses in an isogenic pair of wild-type and Gαq(Q209P) knock-in HEK293A cells established in our previous study [17]. Differential expression analysis identified 997 significantly upregulated genes and 1,038 downregulated genes in Gαq(Q209P) knock-in cells relative to wild-type cells, indicating extensive transcriptomic rewiring (Supplementary Fig. 1a). Because transcriptional output is closely linked to chromatin accessibility, we next profiled genome-wide accessibility using ATAC-seq. Principal component analysis showed clear separation between Gαq(Q209P) knock-in

and wild-type cells, indicating widespread alterations in chromatin accessibility associated with the knock-in mutation (Supplementary Fig. 1b). Integration of the RNA-seq and ATAC-seq datasets further revealed that genes upregulated in Gαq(Q209P) knock-in cells displayed increased accessibility around their transcription start sites (TSSs), whereas downregulated genes showed reduced accessibility at the corresponding regions (Supplementary Fig. 1c). Consistent with these changes, Gαq(Q209P) knock-in cells exhibited enhanced anchorage-independent growth, as evidenced by increased colony number and size in soft-agar assays (Supplementary Fig. 1d). Moreover, inducible knockdown of Gαq selectively suppressed colony formation in Gαq(Q209P) knock-in cells but had minimal effect in wild-type cells, indicating that the knock-in mutation confers a selective dependency on oncogenic Gαq signaling for sustained growth (Supplementary Fig. 1e).

To further assess the disease relevance of this Gαq(Q209P)-associated transcriptional and chromatin state, we compared the gene expression changes induced by Gαq(Q209P) knock-in in HEK293A cells with the TCGA UM cohort using a framework adapted from a prior study of Gαq-driven UM, in which Gαq-negative skin cutaneous melanoma (SKCM) samples were used as the reference control because of the limited number of Gαq-negative UM cases [14]. Using this approach, we found a highly significant overlap between genes upregulated in Gαq(Q209P) knock-in HEK293A cells and genes overexpressed in Gαq-positive human UM, defined as tumors harboring mutation or amplification in *GNAQ*, *GNA11*, or *CYSLTR2* (hypergeometric test, $p < 3.598 \times 10^{-39}$; see Methods). These findings support the use of the isogenic HEK293A Gαq(Q209P) system as a tractable model for identifying vulnerabilities associated with oncogenic Gαq signaling.

UM typically has a low mutation burden and remains relatively stable at the nucleotide level during metastasis [25], suggesting that non-genetic regulatory mechanisms may contribute to disease progression. Patient-level transcriptomic analyses have also revealed distinct gene expression programs associated with Gαq-driven UM [14]. In addition, in our previous study, oncogenic Gαq mutations induced broad transcriptional changes and conferred enhanced sensitivity to transcription-associated inhibitors [17]. Together with the transcriptional and chromatin rewiring observed here in Gαq(Q209P) knock-in HEK293A cells, these findings suggested that epigenetic regulators, as key modulators of transcriptional output and chromatin state, might represent a tractable source of vulnerabilities in Gαq-driven UM. We therefore performed a CRISPR/Cas9 knockout screen using a previously characterized focused sgRNA library targeting 450 epigenetic regulators, with seven sgRNAs per gene [18], together with 100 non-targeting control sgRNAs[19]."

References:

Zhang G, et al. Reciprocal positive regulation between BRD4 and YAP in GNAQ-mutant uveal melanoma cells confers sensitivity to BET inhibitors. Pharmacol Res, 2022, 184:106464.

Feng X, et al. A platform of synthetic lethal gene interaction networks reveals that GNAQ uveal melanoma oncogene controls the Hippo pathway through FAK. Cancer Cell, 2019, 35(3):457-72.

3. Prior studies implicate TEAD family proteins in epigenetic regulation via enhancers involving p300. The claim of direct YAP-p300 interaction (via WW-KIX domains) lacks robust validation. Current evidence (e.g., AlphaFold prediction) does not rule out indirect mechanisms (e.g., TEAD-mediated interactions), necessitating further biochemical proof.

Response:

We thank the reviewer for raising this important point. We agree that our original data did not sufficiently exclude indirect mechanisms, including a possible TEAD-mediated interaction, and that additional biochemical validation was necessary.

To directly address this concern, we performed two new sets of experiments.

(1) TEAD-dependence test in cells

Because TEAD family proteins have been implicated in enhancer-associated regulation involving p300 (**Stein et al., 2015**), we first asked whether TEAD proteins are required for the YAP-p300 association. Co-immunoprecipitation assays showed that simultaneous knockdown of TEAD1/2/3/4 did not impair the interaction between full-length YAP and full-length p300 (**Supplementary Fig. 7a**). This result argues against a TEAD-mediated bridging mechanism.

(2) Direct binding assay using purified proteins

We then purified recombinant p300 (aa 567-680) and GST-YAP (aa 155-290) proteins and examined their interaction by SPR. p300 bound GST-YAP in a concentration-dependent manner, whereas no binding was observed with GST alone (**Supplementary Fig.7b, c**). Thus, YAP and p300 are capable of direct physical interaction in the absence of other cellular factors.

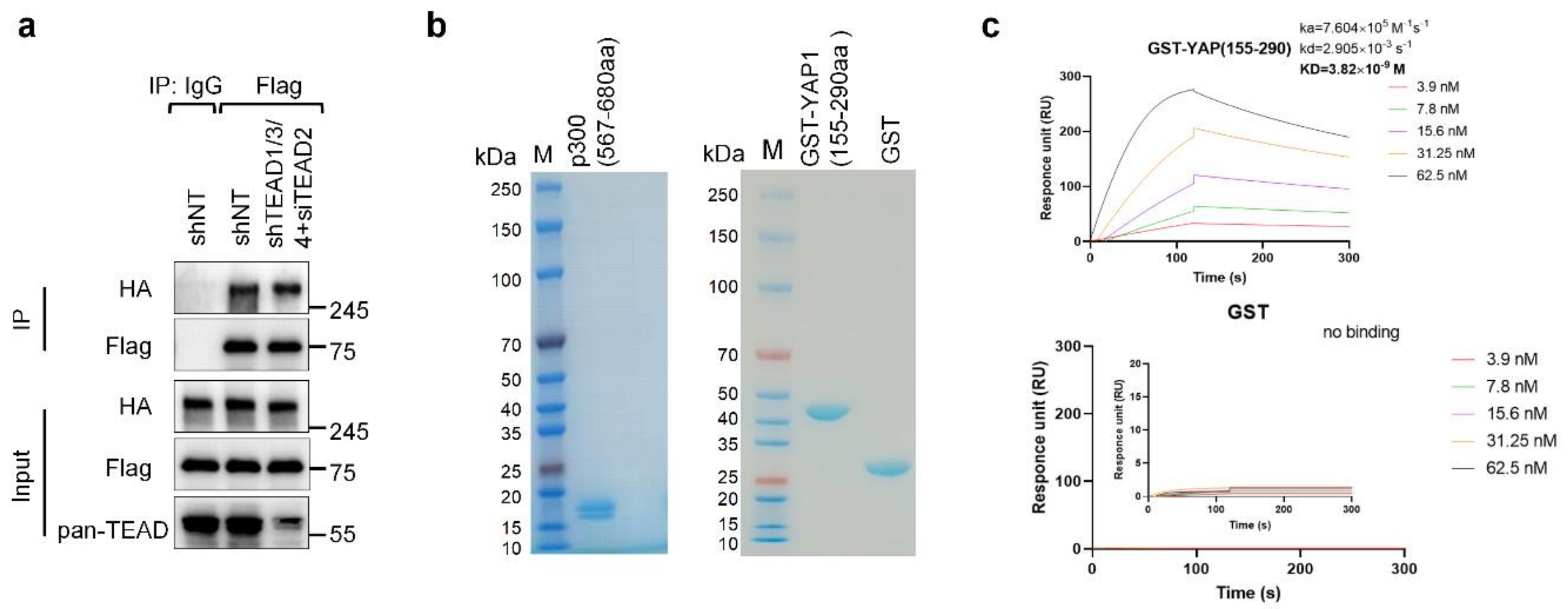

Supplementary Fig. 7: YAP directly binds p300 in a TEAD-independent manner.

a, TEAD knockdown does not impair the interaction between YAP and p300. HEK293T cells stably expressing shRNAs targeting TEAD1, 3, and 4 were transfected with TEAD2 siRNA together with YAP and p300 constructs. Cells were harvested 48 h after transfection and subjected to co-immunoprecipitation followed by immunoblotting with the indicated antibodies. Cells expressing non-targeting shRNA (shNT) served as controls. **b**, SDS–PAGE analysis of purified recombinant proteins used for surface plasmon resonance (SPR) assays. c, SPR analysis of the interaction between recombinant p300 (567-680 aa) and GST-YAP (155-290 aa). p300 bound GST-YAP in a concentration-dependent manner, whereas no binding was detected with GST alone.

In light of these new data, we have revised the manuscript accordingly (**Results section, lines 622-632**). Specifically, we now conclude that YAP directly interacts with p300 in a TEAD-independent manner, and that this interaction involves the WW domains of YAP and the KIX domain of p300.

We believe these new experiments directly address the reviewer's concern and substantially strengthen the conclusion.

The revised Results section (lines 622-632) now reads as follows:

"Given that TEAD family proteins have been implicated in enhancer-associated transcriptional regulation involving p300 [29], we next asked whether the YAP-p300 association depends on TEAD proteins. Simultaneous knockdown of TEAD1/2/3/4 did not impair the interaction between YAP and p300 in co-IP assays (Supplementary Fig. 7a), arguing against a TEAD-mediated bridging mechanism. To determine whether YAP and p300 bind directly, we purified recombinant p300 (567-680 aa) and GST-YAP (155-290 aa) proteins and examined their interaction using surface plasmon resonance (SPR). p300 bound GST-YAP in a concentration-dependent manner, whereas binding to GST alone was not detected (Supplementary Fig. 7b, c). Together, these results demonstrate that YAP directly

binds to p300 in a TEAD-independent manner, and that this interaction is mediated by the WW domains of YAP and the KIX domain of p300."

References:

Stein C, et al. YAP1 exerts its transcriptional control via TEAD-mediated activation of enhancers. PLoS Genet, 2015, 11(8): e1005465.

4. The use of 92.1 cells (primary tumor-derived, BAP1 wild-type) to model metastatic uveal melanoma is problematic. Metastatic disease, often BAP1-mutant and liver-localized, represents the key clinical challenge. The chosen model limits translational relevance to metastasis prevention/treatment.

Response:

Thank you for this insightful comment. We agree that metastatic uveal melanoma, particularly liver metastatic disease with BAP1 deficiency, represents the most clinically relevant setting for evaluating translational potential. In response to this concern, we have incorporated additional *in vivo* experiments using MM28 cells, which were derived from a BAP1-deficient liver metastasis of uveal melanoma (**Amirouchene-Angelozzi N, 2014**), and these new data are now presented in revised **Fig. 7 and Supplementary Fig. 14**. The corresponding result has been added to the revised Results section (**lines 843–856**).

The revised Results section (lines 843–856) now reads as follows:

"We next asked whether p300 depletion could suppress metastatic progression of Gαq-mutant UM *in vivo*. To address this question, we used MM28 cells, a UM cell line derived from a BAP1-deficient liver metastasis[32]. MM28 cells lack BAP1 expression (Supplementary Fig. 14a), and doxycycline-induced p300 knockdown efficiently reduced both p300 and H3K27ac levels (Supplementary Fig. 14b) and markedly impaired clonogenic growth (Supplementary Fig. 14c). We evaluated the effect of p300 depletion in an intrasplenic metastasis model using luciferase-labeled MM28 cells (Fig. 7a). Doxycycline-induced p300 knockdown significantly reduced tumor bioluminescence (Fig. 7b, c). At the experimental endpoint, this model showed strong hepatotropism (Supplementary Fig. 14d), consistent with the liver-dominant metastatic pattern of UM in patients. p300 knockdown also decreased hepatic tumor burden, as indicated by gross liver appearance (Supplementary Fig. 14e), liver-to-body weight ratio (Fig. 7d), and whole-liver H&E sections showing reduced metastatic involvement (Fig. 7e). Moreover, p300 knockdown reduced the percentage of Ki67-positive cells in liver metastatic lesions (Fig. 7f) and alleviated body weight loss (Fig. 7g)."

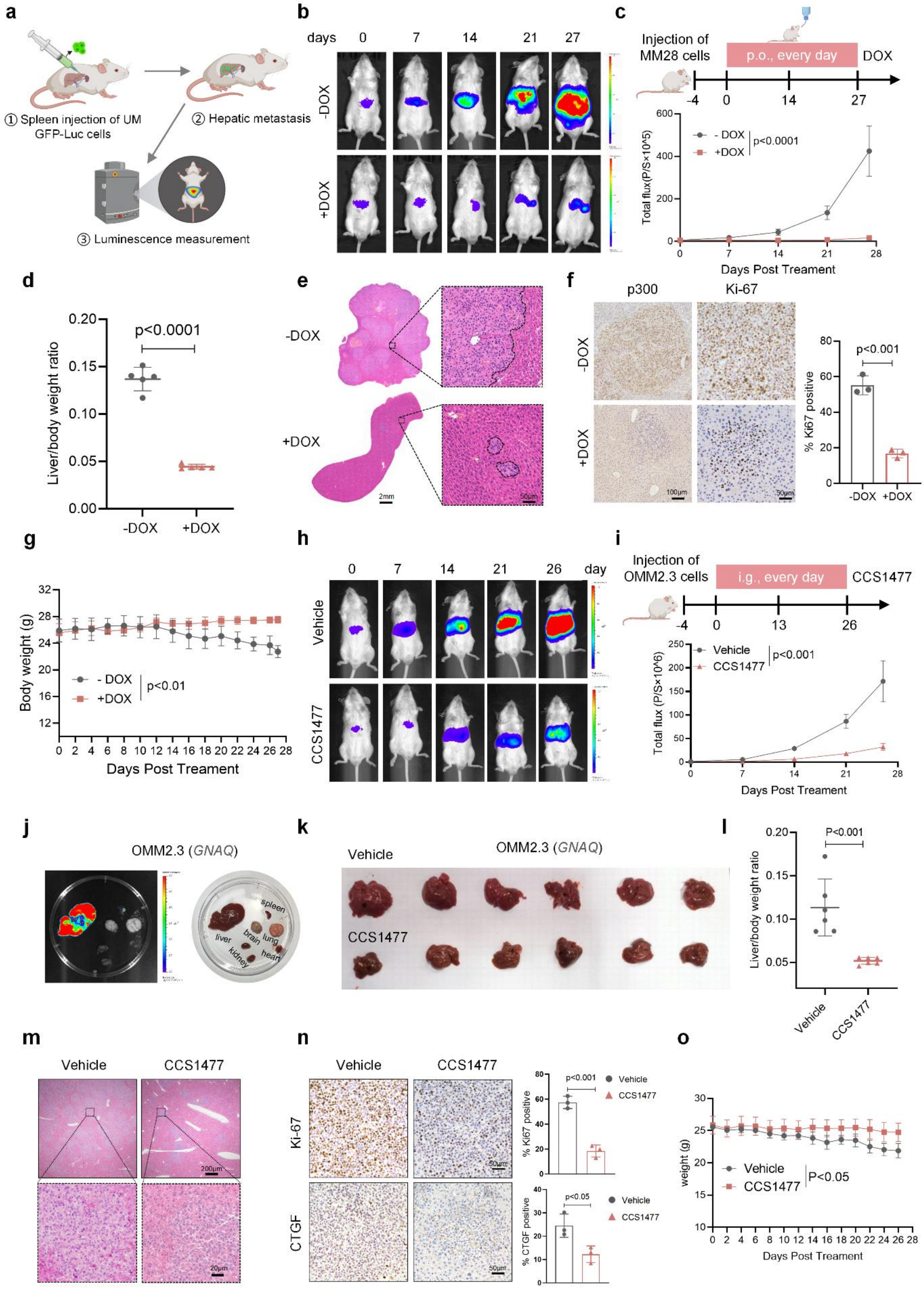

Fig. 7 p300 inhibition suppresses UM cell metastasis *in vivo*.

a, Schematic diagram of the intrasplenic UM-Luc metastatic model. Stable UM-Luc cells were injected into the spleens of NCG mice, and hepatic metastasis was monitored by *in vivo* imaging systems (IVIS).

b and **c**, Representative IVIS images (**b**) and quantitative analysis of tumor burden (**c**) in NCG mice injected with stable MM28-Luc cells expressing inducible p300 shRNA#1 and treated with or without doxycycline (0.5 mg/ml in drinking water). Data are presented as mean ± SD (n = 5 biological replicates). Statistical significance was assessed by two-way ANOVA. **d,** Liver-to-body weight ratio in mice bearing MM28-Luc metastases. Data are presented as mean ± SD (n = 5 biological replicates). Statistical significance was assessed by a two-sided Student's t-test. **e,** Representative H&E staining of liver sections from mice bearing MM28-Luc metastases. **f**, Representative immunohistochemical staining of liver sections from mice bearing MM28-Luc metastases. Quantification is shown on the right. Data are presented as mean ± SD (n = 3 biological replicates). Statistical significance was assessed using a two-sided Student's t-test. **g**, Body weight of mice bearing MM28-Luc metastases. Data are presented as mean ± SD (n = 5 biological replicates). Statistical significance was assessed by two-way ANOVA. **h** and **i**, Representative IVIS images (**h**) and quantitative analysis of tumor burden (**i**) in NCG mice injected with OMM2.3-Luc cells treated with vehicle or CCS1477 (30 mg/kg). Data are presented as mean ± SD (n = 6 biological replicates). Statistical significance was assessed by two-way ANOVA. **j**, Representative *ex vivo* images of organs collected at the experimental endpoint from mice bearing OMM2.3-Luc metastases. **k**, Images of livers collected from mice bearing OMM2.3-Luc metastases. **l**, Liver-to-body weight ratio in mice bearing OMM2.3-Luc metastases. Data are presented as mean ± SD (n = 6 biological replicates). Statistical significance was assessed using a two-sided Student's t-test. **m**, Representative H&E staining of liver sections from mice bearing OMM2.3-Luc metastases. **n**, Representative immunohistochemical staining of liver sections from mice bearing OMM2.3-Luc metastases. Quantification is shown on the right. Data are presented as mean ± SD (n = 3 biological replicates). Statistical significance was assessed using a two-sided Student's t-test. **o**, Body weight of mice bearing OMM2.3-Luc metastases. Data are presented as mean ± SD (n = 6 biological replicates). Statistical significance was assessed by two-way ANOVA. Source data are provided as a Source Data file.

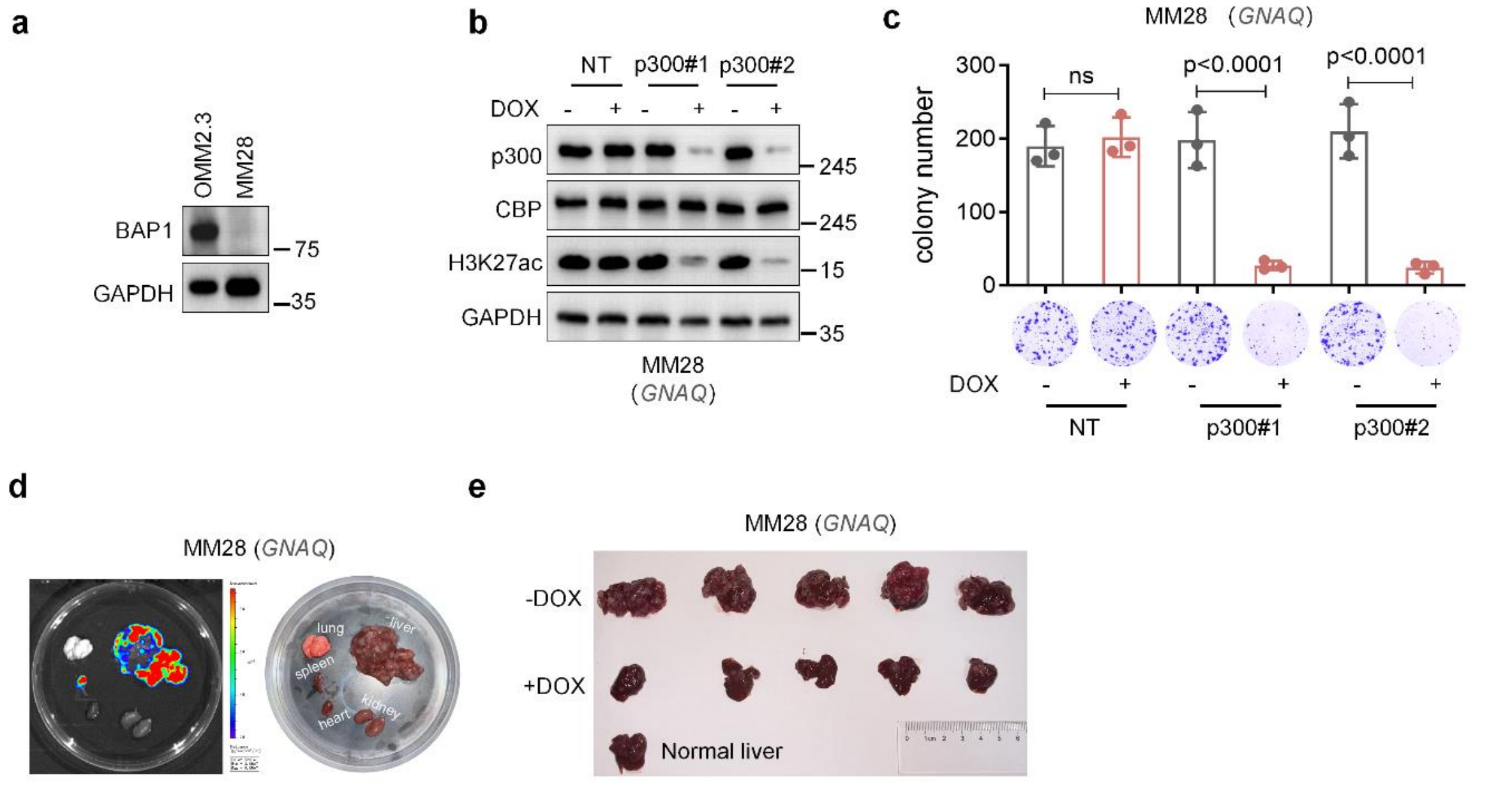

Supplementary Fig. 14: p300 knockdown suppresses *in vitro* growth and liver metastasis of Gαq-mutant, BAP1-deficient UM MM28 cells.

a, Immunoblotting analysis of BAP1 expression in MM28 cells, with OMM2.3 cells used as a control. **b,** Inducible knockdown of p300 in MM28 cells. Stable MM28 cells expressing inducible non-targeting shRNA (shNT) or two independent p300-targeting shRNAs (shp300#1 and shp300#2) were generated. Cells were treated with or without doxycycline (200 ng/ml) for 72 h, and p300 knockdown was assessed by immunoblotting. **c,** p300 knockdown inhibits clonogenic growth of MM28 cells. Stable MM28 cells expressing inducible shNT or p300-targeting shRNAs were treated with or without doxycycline (200 ng/ml), and clonogenic growth was assessed by colony-formation assay. Data are presented as mean ± SD (n = 3) from one representative experiment out of three. Statistical significance was determined by two-sided Student's t-test; ns, not significant. **d,** Representative *ex vivo* images of organs collected at the experimental endpoint from mice bearing MM28-Luc metastases. **e,** Images of livers collected from the mice bearing MM28-Luc metastases.

At the same time, we have retained the original 92.1 data, but moved them to the Supplementary Information, because we agree that this model should not serve as the principal basis for translational claims regarding metastatic UM. Rather, in the revised manuscript, the translational relevance of our *in vivo* findings relies primarily on the MM28 model and on the OMM2.3 pharmacologic model, while the 92.1 model is presented as an additional, independent UM model showing concordant effects of p300 inhibition.

Although 92.1 cells are derived from a primary tumor and are BAP1 wild-type, we believe their inclusion as a supportive model remains informative. The 92.1 cell line was established from a primary uveal melanoma, and prior characterization indicates that the donor patient subsequently developed widespread metastatic disease (**Jager MJ, 2016**). In addition, reviews of UM model systems indicate that 92.1 has been widely used in preclinical mouse and zebrafish studies to evaluate candidate therapeutic strategies (**Paradis JS, 2021; Arang N, 2023; van den Bosch QCC, 2024**). We therefore retained these results as secondary supportive evidence, rather than as the main translational anchor of the study.

Importantly, after revision, our main *in vivo* conclusion is now supported by multiple UM models serving distinct roles: MM28 as a clinically relevant BAP1-deficient liver metastasis-derived model, OMM2.3 as an additional metastatic UM model for pharmacologic validation, and 92.1 as an independent supportive model. We believe that these revisions substantially strengthen the translational relevance of the study and better align our conclusions with the clinical challenge of metastatic UM.

References:

Amirouchene-Angelozzi N, et al. Establishment of novel cell lines recapitulating the genetic landscape of uveal melanoma and preclinical validation of mTOR as a therapeutic target. Mol Oncol, 2014, 8(8):1508-20.
Jager MJ, et al. Uveal melanoma cell lines: where do they come from? Trans Am Ophthalmol Soc 2016, 114: T5
Paradis JS, et al. Synthetic lethal screens reveal cotargeting FAK and MEK as a multimodal precision therapy for GNAQ-driven uveal melanoma. Clin Cancer Res 2021, 27 (11): 3190-3200.
Arang N, et al. High-throughput chemogenetic drug screening reveals PKC-RhoA/PKN as a targetable signaling vulnerability in *GNAQ*-driven uveal melanoma. Cell Rep Med, 2023, 4(11): 101244
van den Bosch QCC, et al. Uveal melanoma modeling in mice and zebrafish. Biochim Biophys Acta Rev Cancer. 2024, 1879(1):189055.

Minor Comments

1. Clarify the methodology for generating HEK293A Gq(Q209L) knock-in cells (e.g., CRISPR, site-directed mutagenesis).

Response: Thank you for this helpful comment. In our study, the engineered HEK293A cells carry the Gαq (Q209P) mutation and were generated by CRISPR/Cas9-mediated homology-directed repair (HDR) at the endogenous *GNAQ* locus, rather than by plasmid-based site-directed mutagenesis. We have now clarified this methodology in the Materials and Methods section (**lines 110-131**), as follows:

"Generation of Gαq (Q209P) knock-in HEK293A cells

Gαq (Q209P) knock-in HEK293A cells were generated using a CRISPR/Cas9-mediated homology-directed repair (HDR) strategy. A gene-specific single guide RNA (sgRNA) and a donor template encoding the Q209P mutation were designed using Benchling. The sgRNA was cloned into the px459 v2 plasmid (Addgene #62988), which co-expressed Cas9 and the sgRNA. A single-stranded oligodeoxynucleotide (ssODN) donor template containing the Q209P mutation and flanking homology arms was used to mediate HDR. HEK293A cells were co-transfected with the sgRNA/Cas9 plasmid and the ssODN donor template, followed by puromycin selection (1 μg/ml) for 3 days. Surviving cells were subsequently isolated as single cells into 96-well plates by flow cytometric sorting. Individual clones were expanded, and genomic DNA was extracted using the PureLink Genomic DNA Mini Kit (Thermo

Fisher). Clones were screened by PCR and Sanger sequencing to identify knock-in events, and a validated heterozygous clone was used for subsequent experiments. Functional characterization of this clone, including activation of YAP signaling downstream of mutant Gαq, has been reported previously [17]. The sgRNA sequence, ssODN donor template, and PCR primers used for screening are listed below:

sgRNA: TGGTCGATGTAGGGGGCCAA;

ssODN:

CCACGAGAACTTGATCATATTCACTAAGCGCTACTAGAAACATGATAGAGGTGACATTTTCAAAGCAGTGGATCCATTTTCTTCTCTCTGATCTTGGGCCCCCTACATCGACCATTCTGCAAGGTTAACA;

Primers: TTTGCCAGTAGGGCTTAACACA, GGATGACTCACTCCAACACTCC."

Reference:

Zhang GM, et al. Reciprocal positive regulation between BRD4 and YAP in GNAQ-mutant uveal melanoma cells confers sensitivity to BET inhibitors. Pharmacol Res 2022, 184: 106464.

2. Provide details for the CTGF luciferase reporter plasmid (e.g., promoter region, response elements) and explain how p300-mediated histone modifications influence plasmid reporter activity.

Response:

We thank the reviewer for this important comment. Information regarding the CTGF luciferase reporter, including the promoter region (-250 to 0 bp) and the TEAD-binding site, has been added to **Fig. 2g**.

In the luciferase assay, p300 knockdown significantly reduced CTGF promoter activity. In addition, we performed H3K27ac ChIP-qPCR at the endogenous CTGF promoter using primers spanning the TEAD-binding site and observed a marked reduction in H3K27ac upon p300 depletion. Together, these results support a role for p300 in promoting CTGF transcription in association with increased H3K27 acetylation at its promoter (**Supplementary Fig. 4d**).

Fig. 2g

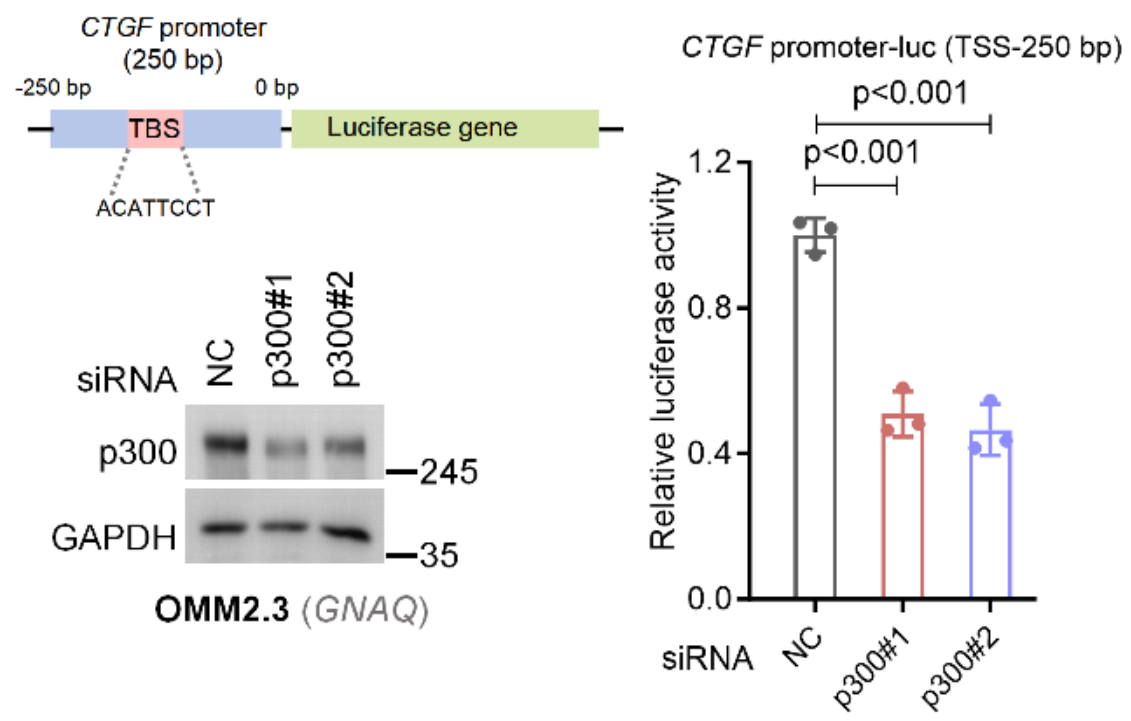

Supplementary Fig. 4d

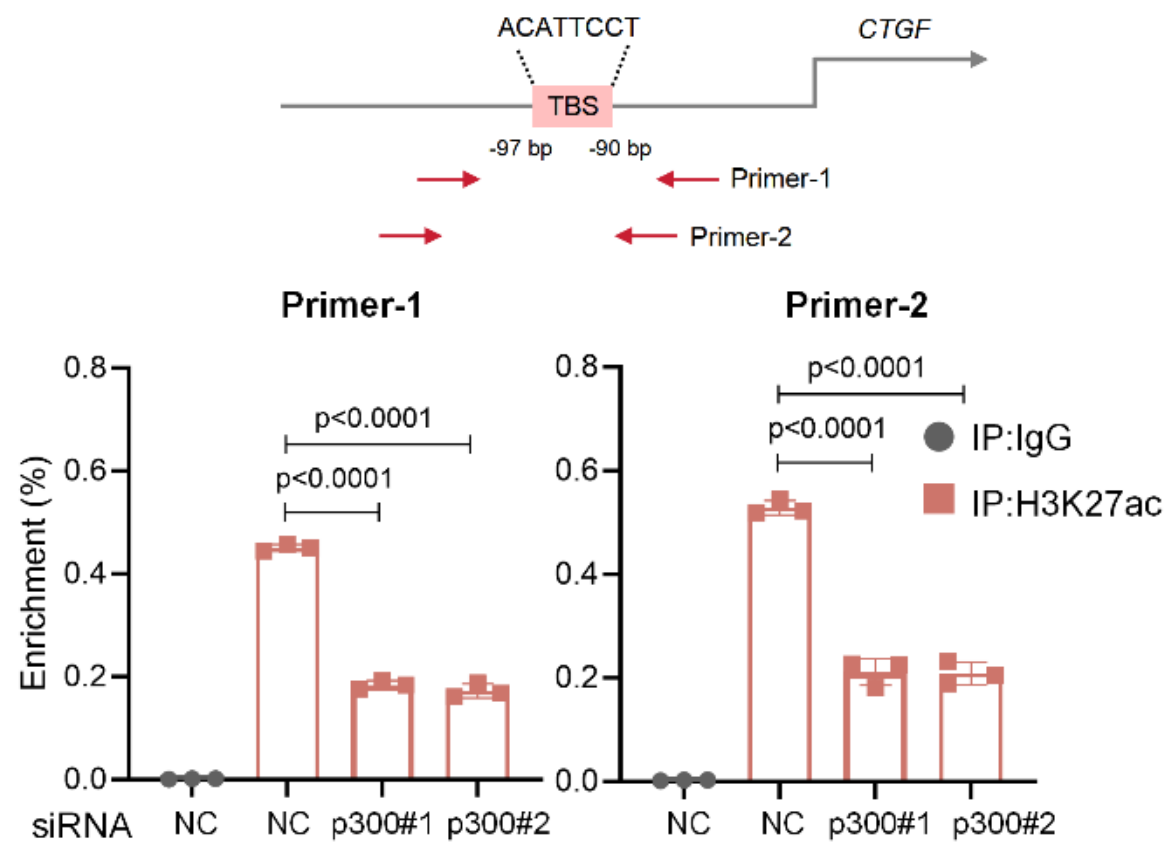

Fig. 2g *CTGF* luciferase reporter assay in OMM2.3 cells transfected with the indicated siRNAs. The reporter contains the CTGF promoter region (−250 to 0 bp) with the TEAD-binding site. Knockdown efficiency was confirmed by immunoblotting. Luciferase activity was measured 48 h after transfection. TBS, TEAD binding site. Data are presented as mean ± SD (n = 3 biological replicates). Statistical significance was assessed by one-way ANOVA with Tukey's multiple comparisons test.

Supplementary Fig. 4d H3K27ac ChIP–qPCR analysis at the endogenous CTGF promoter in OMM2.3 cells transfected with the indicated siRNAs. Upper panel, schematic of the CTGF promoter region and primer locations. Cells were harvested 48 h after transfection. Data are presented as mean ± SD (n = 3 biological replicates). Statistical significance was assessed by one-way ANOVA followed by Tukey's multiple comparisons test.

3. Strengthen evidence for direct YAP-p300 interaction (e.g., in vitro binding assays beyond AlphaFold predictions).

Response:

Thank you for this comment. To strengthen the evidence for direct YAP-p300 interaction beyond AlphaFold prediction, we performed SPR assays using purified recombinant proteins. p300 (aa 567-680) bound GST-YAP (aa 155-290) in a concentration-dependent manner, while no binding was observed with GST alone (**Supplementary Fig. 7, also see our response to Major Concern #3 for details**). These data provide direct biochemical evidence for a physical interaction between YAP and p300.

4. In Figure 3a, label blots with antibody names (e.g., "HA," "Flag") instead of "HA-p300" or "Flag-YAP" for clarity. The same for other related figures.

Response:

Thank you for this comment. We have revised the blot labels in Figure 3a and the related figures by indicating the antibody names (for example, HA and Flag) instead of construct names such as HA-p300 or Flag-YAP, to improve clarity and consistency.

5. The proximity ligation assay (PLA) signal in Figure 3b appears weak.

Response:

Thank you for this comment. We replaced Figure 3b with images reprocessed from the original PLA data by uniformly adjusting brightness and contrast across the entire image. These adjustments were made only for display purposes and did not alter the underlying image information. The revised figure presents the PLA signal more clearly.

Figure 3b

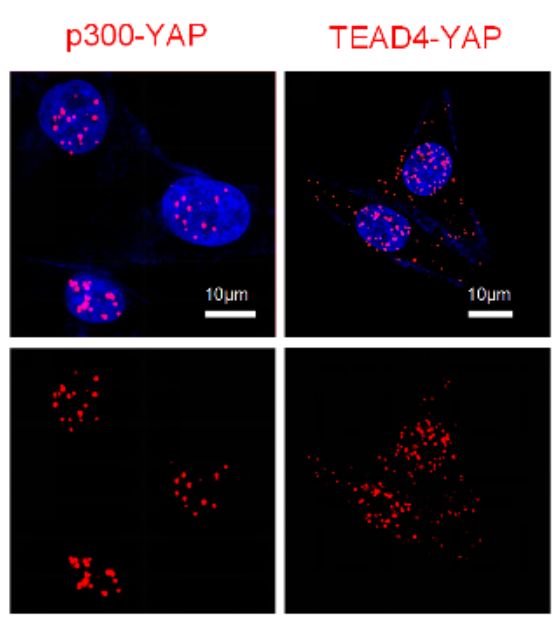

Figure 3b, Endogenous interaction between p300 and YAP in the nucleus of OMM2.3 cells, as assessed by proximity ligation assay (PLA). Red dots indicate PLA signals. Nuclei were counterstained with DAPI (blue). Endogenous TEAD4-YAP interaction was used as a positive control.

6. Explain the omission of CYR61 in Figure 2f, given its prominence in other figures.

Response:

Thank you for this comment. We have now included the *CYR61* data in **Figure 2f** to improve consistency with the other panels in this figure set. The revised figure shows that *CYR61*, similar to the other YAP target genes analyzed, is also reduced upon CCS1477 treatment in OMM2.3 cells.

Figure 2e

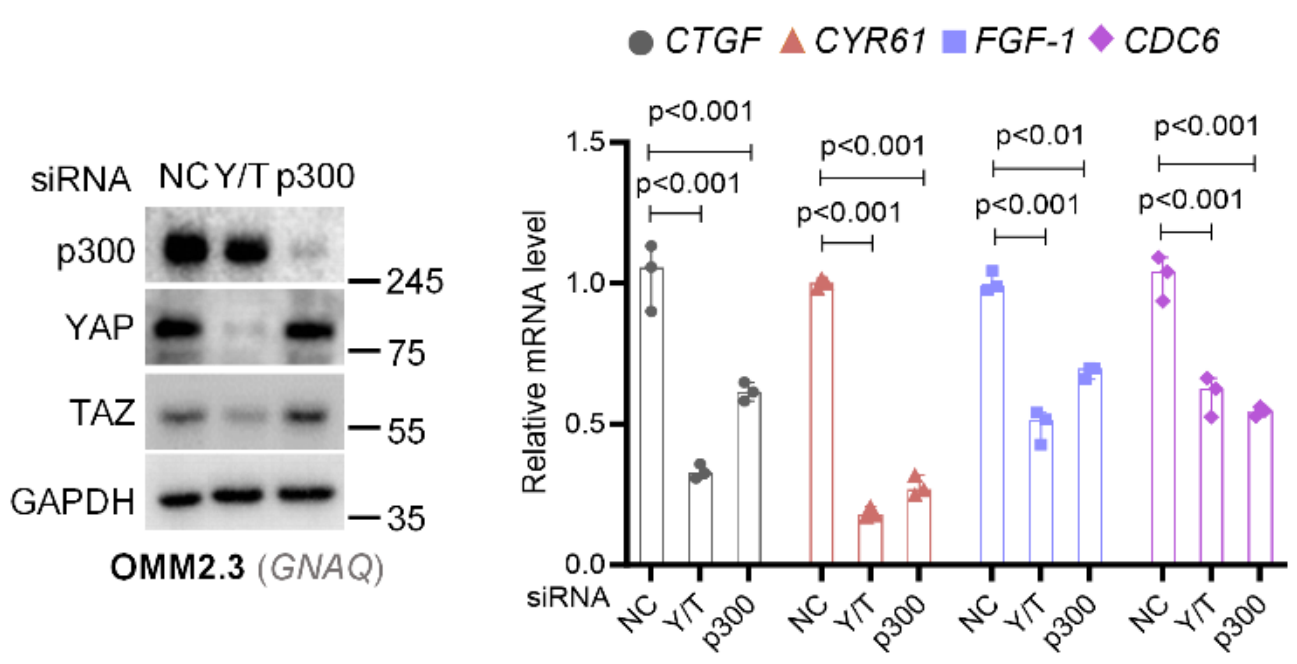

Figure 2f

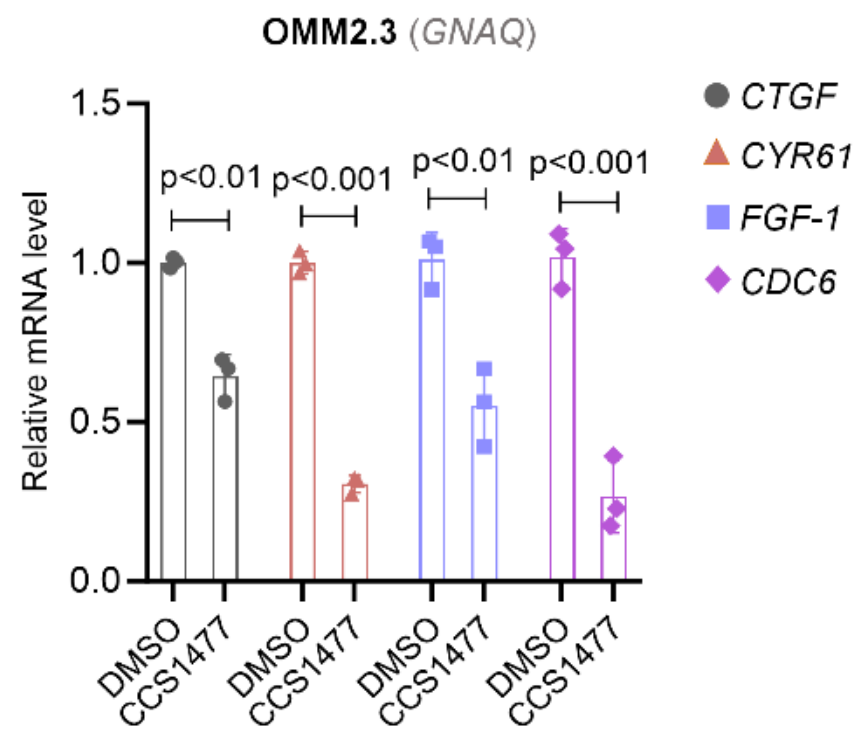

Figure 2e, RT-qPCR analysis of representative YAP target genes in OMM2.3 cells after transfection with the indicated siRNAs. Knockdown efficiency was confirmed by immunoblotting. Data are presented as mean ± SD (n = 3 biological replicates). Statistical significance was assessed by one-way ANOVA followed by Tukey's multiple comparisons test.

Figure 2f, Inhibition of p300 by CCS1477 suppresses mRNA expression of representative YAP target genes in OMM2.3 cells. Data are presented as the mean ± SD (n = 3 biological replicates). Statistical significance was assessed using a two-sided Student's t-test.

7. Extend mechanistic analyses to TAZ, which also contains a WW domain and may share p300-dependent regulation.

Response:

We thank the reviewer for this valuable suggestion. To address this point, we extended the mechanistic analysis to TAZ, which also contains a WW domain. Co-immunoprecipitation assays showed that TAZ interacts with p300, whereas deletion of the TAZ WW domain abolished this interaction. Conversely, deletion of the p300 KIX domain disrupted the interaction with TAZ, indicating that the TAZ WW domain and the p300 KIX region are required for their association. Functionally, p300 knockdown markedly suppressed TAZ-dependent reporter activity. Consistently, wild-type p300 enhanced TAZ-dependent reporter activity, whereas p300-ΔKIX failed to do so. Together, these findings extend our mechanistic model from YAP to TAZ and support a shared, domain-dependent regulatory role for p300 in YAP/TAZ transcriptional activity. These results are now included in **Supplementary Fig. 8**.

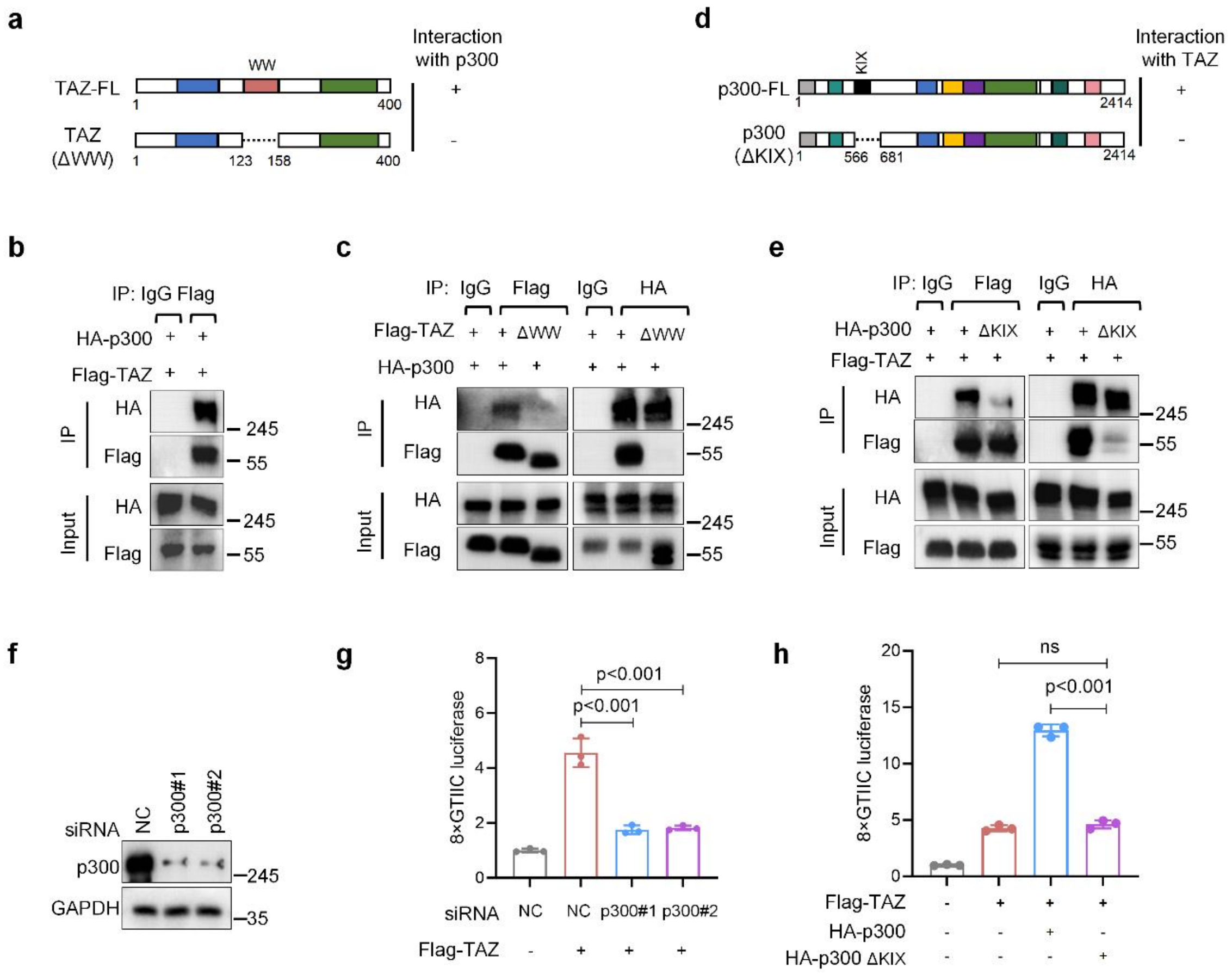

Supplementary Fig. 8 p300 interacts with TAZ and enhances TAZ-dependent transcription.

a, Co-immunoprecipitation (co-IP) assays showing the interaction between p300 and TAZ. Indicated constructs were co-transfected into HEK293T cells. HEK293T cells were transfected with the indicated constructs. TAZ was immunoprecipitated with anti-Flag antibody, and co-immunoprecipitated p300 was detected by immunoblotting with the indicated antibodies. **b**, Schematic diagram of Flag-tagged TAZ constructs used in this study. **c**, The WW domain of TAZ mediates its interaction with p300. HEK293T cells were transfected with HA-tagged p300 together with Flag-tagged full-length or WW-deleted TAZ constructs, followed by co-IP and immunoblotting as in panel **a**. **d,** Schematic diagram of HA-tagged p300 constructs used in this study. **e**, The KIX domain of p300 mediates its interaction with TAZ. HEK293T cells were transfected with Flag-tagged TAZ together with HA-tagged wild-type or KIX-deleted p300 constructs, followed by co-IP and immunoblotting as in panel **a**. **f and g,** p300 knockdown suppresses TAZ-dependent transcription. HEK293T cells were transfected with negative control siRNA (NC) or two independent siRNAs targeting p300 (p300#1 and p300#2). Twenty-four hours later, cells were co-transfected with TAZ and the 8 × GTIIC-Luc reporter. At 72 h after the initial siRNA transfection, p300 knockdown efficiency was assessed by immunoblotting (**f**), and luciferase activities were measured (**g**). Firefly luciferase activity was normalized to Renilla luciferase activity. Data are presented as mean ± SD (n = 3 biological replicates). Statistical significance was assessed by one-way ANOVA followed by Tukey’s multiple comparisons test. **h,** The KIX domain is required for p300-mediated enhancement of TAZ-dependent transcription. HEK293T cells were transfected with the 8 × GTIIC-Luc reporter together

with TAZ alone or in combination with p300 or p300-ΔKIX. Firefly luciferase activity was normalized to Renilla luciferase activity. Data are presented as mean ± SD (n = 3 biological replicates). Statistical significance was assessed by one-way ANOVA followed by Tukey's multiple comparisons test.

8. Validate YAP-K13 as the sole acetylation site (e.g., mass spectrometry) and assess genome-wide transcriptional changes in YAP-K13R or p300-KD models, instead of changes of a few signatures.

Response:

We thank the reviewer for this helpful suggestion.

First, we would like to clarify a potential misunderstanding regarding the acetylation-deficient YAP mutant used in this study. The construct employed here is YAP (13KR), in which all 13 lysine residues in YAP were substituted with arginine, rather than a single K-to-R mutation. This mutant was designed to abolish p300-mediated acetylation of YAP globally. Consistent with this, co-expression of p300 robustly increased acetylation of wild-type YAP but not YAP (13KR) (revised **Fig. 4c**). To make this point clearer, we added a schematic diagram of the YAP (13KR) mutant in new Fig. 4c and revised the corresponding text in the Results section (**lines 692–697**) and Fig. 4 legend.

The revised Results section (lines 692–697) now reads as follows:

"To test whether YAP acetylation is required for YAP-dependent transcription, we generated an acetylation-deficient YAP mutant, YAP (13KR), in which all 13 lysine residues were substituted with arginine (Fig. 4c). As expected, co-expression of p300 markedly increased acetylation of wild-type YAP but not YAP (13KR) (Fig. 4c), confirming that the 13KR mutant abolishes p300-mediated acetylation of YAP."

Second, in response to the reviewer's suggestion to assess transcriptional changes beyond a limited number of YAP target genes, we performed RNA-seq in three biological replicates in HEK293T cells co-expressing p300 with either wild-type YAP or YAP (13KR). Because the focus of this section is whether YAP acetylation affects YAP-dependent transcriptional output, we analyzed a predefined YAP-activated gene set (n = 1,186 genes, as defined in the manuscript). Sample-to-sample distance analysis showed strong similarity between the YAP + p300 and YAP (13KR) + p300 groups (new **Fig. 4d**). Consistently, co-expression of p300 with either wild-type YAP or YAP (13KR) increased expression of the same predefined

YAP-activated gene set to a comparable extent (new **Fig. 4e**). These findings were further supported by qPCR analysis of the canonical YAP target genes *CTGF* and *CYR61* (revised **Fig. 4f**).

Together, these additional analyses strengthen our conclusion that p300 regulates YAP transcriptional activity primarily through modulation of histone acetylation rather than through YAP acetylation.

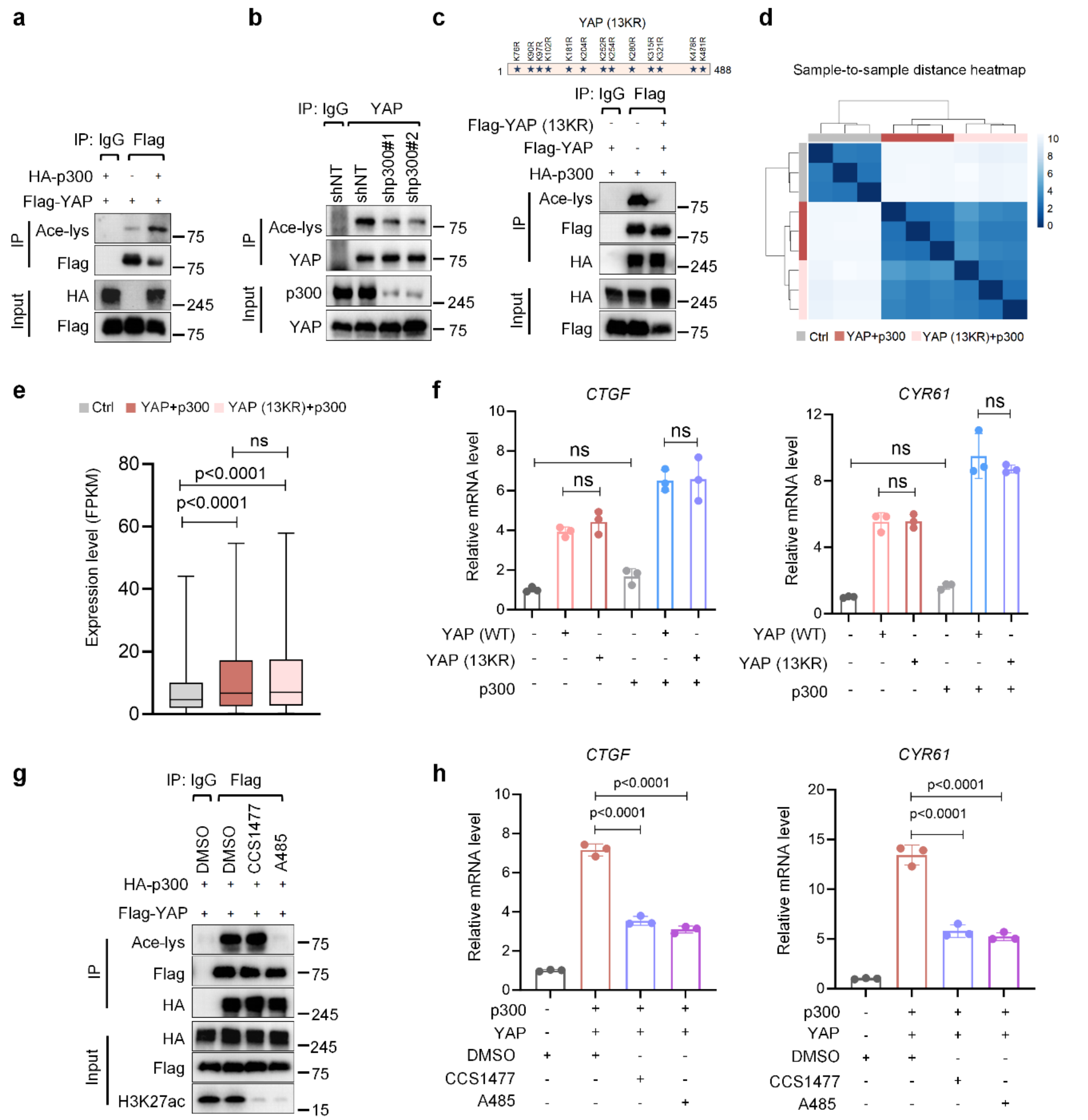

Fig. 4 YAP acetylation is dispensable for p300-mediated YAP-dependent transcription.

a, HEK293T cells were co-transfected with Flag-tagged YAP and HA-tagged p300 constructs. YAP acetylation was assessed using an anti-acetyl-lysine antibody following immunoprecipitation with an anti-Flag antibody. **b**, HEK293T cells stably expressing non-targeting (shNT) or two independent p300-

targeting shRNAs were subjected to immunoprecipitation with an anti-YAP antibody. Endogenous YAP acetylation was assessed using an anti-acetyl-lysine antibody. **c,** Schematic diagram of the acetylation-deficient YAP mutant, YAP (13KR), in which all 13 lysine residues were substituted with arginine. HEK293T cells were co-transfected with HA-tagged p300 and either Flag-tagged YAP or Flag-tagged YAP (13KR). YAP acetylation was assessed as in panel **a**. p300 increased acetylation of wild-type YAP but not YAP (13KR). **d,** Sample-to-sample distance heatmap showing high similarity between the YAP + p300 and YAP (13KR) + p300 groups based on expression of a predefined YAP-activated gene set (n = 1,186 genes) in HEK293T cells. RNA-seq was performed with 3 biological replicates. **e,** Co-expression of p300 with either wild-type YAP or YAP (13KR) similarly increased expression of the predefined YAP-activated gene set (n = 1,186 genes). Gene expression levels, quantified as FPKM from RNA-seq data, are shown as box-and-whisker plots. Boxes indicate the 25th to 75th percentiles, center lines indicate the medians, and whiskers indicate the 2.5th to 97.5th percentiles. Statistical significance was assessed using the Friedman test. **f**, Ectopic expression of wild-type YAP or YAP (13KR) similarly increased mRNA levels of YAP target genes *CTGF* and *CYR61*, with or without co-transfection of p300 in HEK293T cells. Data are presented as mean ± SD (n = 3 biological replicates). Statistical significance was assessed by one-way ANOVA followed by Tukey's multiple comparisons test. **g**, HEK293T cells co-transfected with HA-tagged p300 and Flag-tagged YAP constructs were treated with the p300 bromodomain inhibitor CCS1477 (2 μM) or the p300 HAT domain inhibitor A485 (2 μM) for 48 h. YAP acetylation and global H3K27ac levels were assessed by immunoprecipitation and immunoblotting, respectively. **h**, CCS1477 and A485 similarly reduced mRNA levels of *CTGF* and *CYR61*. HEK293T cells from panel **g** were harvested for qPCR analysis. Data are presented as mean ± SD (n = 3 biological replicates). Statistical significance was assessed by one-way ANOVA followed by Tukey's multiple comparisons test.

9. Use genetic p300 knockdown/knockout (not just inhibitors) to confirm its role in YAP acetylation and exclude off-target drug effects.

Response:

We thank the reviewer for this suggestion. To assess the role of p300 in YAP acetylation using a genetic approach, we generated HEK293T cells stably expressing non-targeting (NT) control or two independent p300-targeting shRNAs. As shown in the newly added **Fig. 4b**, p300 knockdown reduced endogenous YAP acetylation compared with the NT control. These data support a role for p300 in YAP acetylation and indicate that the effect of p300 inhibitors on YAP acetylation is not due to off-target drug activity. The corresponding result has been added to the revised Results section.

10. In Figure 6 experiments, include a "shRNA negative + DOX" control group to account for DOX-induced artifacts.

Response:

We thank the reviewer for this helpful suggestion. To control for potential doxycycline-related effects, we generated 92.1 and M14 cells expressing inducible non-targeting shRNA (shNT) and performed soft-agar and xenograft experiments under doxycycline treatment. As shown in the newly added **Supplementary Fig. 13**, doxycycline treatment did not affect anchorage-independent growth of shNT-expressing 92.1 or M14 cells, nor did it affect the growth of xenografts derived from shNT-expressing 92.1 cells. These results indicate that the inhibitory effects observed in Fig. 6 are attributable to p300 knockdown rather than doxycycline treatment itself. The corresponding data have been added to the revised manuscript and Supplementary Information.

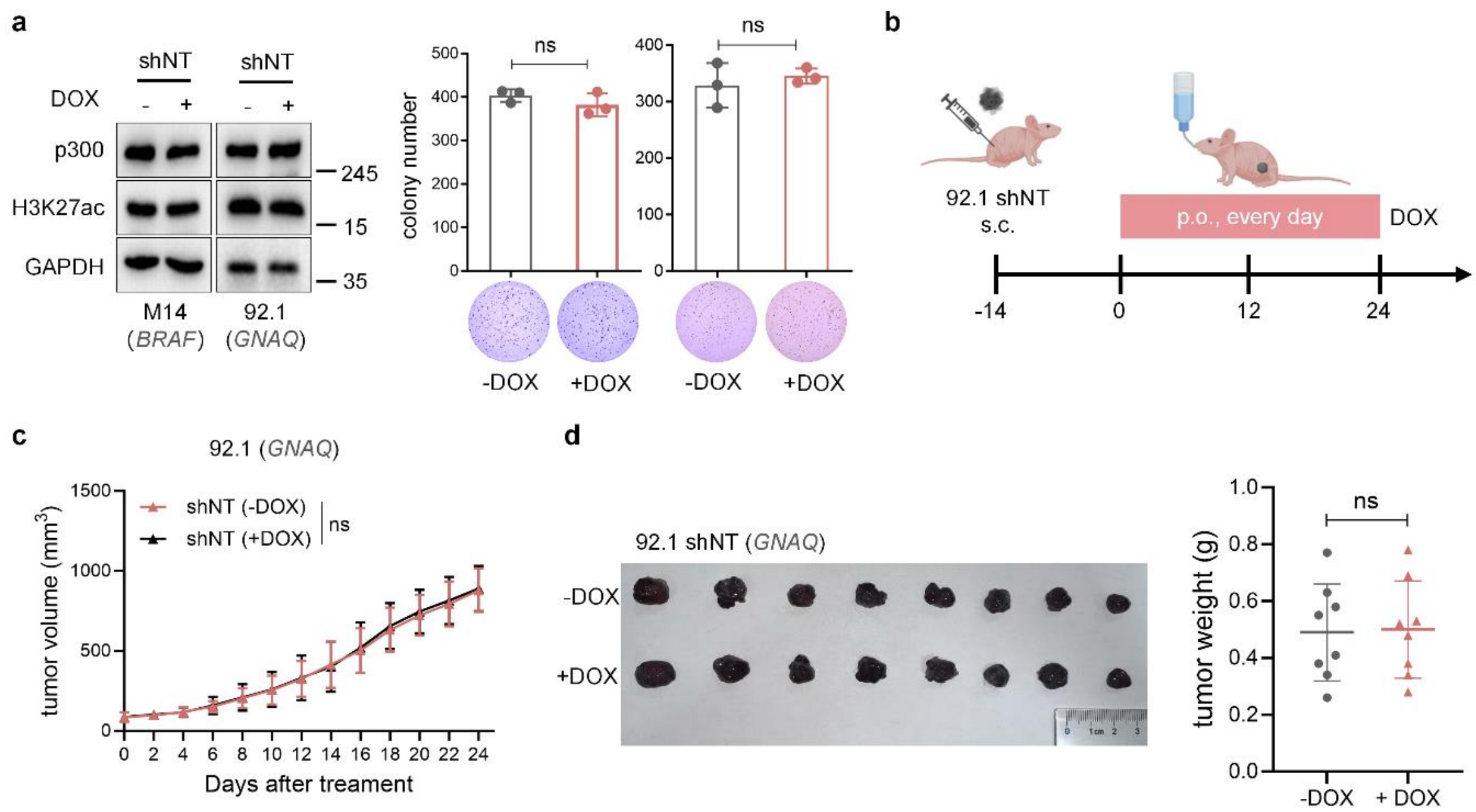

Supplementary Fig. 13: Inducible expression of non-targeting shRNA does not affect cell growth under doxycycline treatment.

a, Inducible expression of non-targeting shRNA (shNT) does not affect anchorage-independent growth of 92.1 or M14 cells. Stable 92.1 and M14 cell lines expressing inducible shNT were generated. Cells were treated with or without doxycycline (200 ng/ml) for 72 h, and p300 protein levels were assessed by immunoblotting (left). Anchorage-independent growth was assessed by soft-agar colony-formation assay after 14 days of doxycycline treatment (right). Data are presented as mean ± SD (n = 3 biological replicates) from one representative experiment out of three. Statistical significance was assessed using two-sided Student's t-test. ns, not significant. **b,** Schematic diagram of the xenograft experiments. **c,** Growth curves of xenografts derived from stable 92.1 cells expressing inducible shNT after subcutaneous implantation into nude mice treated with or without doxycycline (0.5 mg/ml in drinking water). Data are presented as mean ± SD (n = 8 biological replicates). Statistical significance was assessed by two-way ANOVA. **d,** Weights of 92.1-derived xenograft tumors harvested on day 24 after treatment. Data are

presented as mean ± SD (n = 8 biological replicates). Statistical significance was assessed using two-sided Student's t-test.

11. Address grammatical errors and improve clarity (e.g., awkward phrasing in Results/Discussion).

Response: We thank the reviewer for this helpful comment. We have carefully edited the revised manuscript throughout to improve grammar, clarity, and readability. In particular, awkward phrasing in the Results and Discussion sections has been corrected. These language edits were made throughout the manuscript without changing the interpretation of the data or the conclusions of the study.

Reviewer #3 (Remarks to the Author):

The manuscript by Zhang et al reports the identification of p300 as a potential therapeutic target for GNAQ-driven uveal melanoma. CRIPSR screens identify p300 as the top hit synthetically lethal with GNAQ mutation. Mechanistically, p300 directly interacts with YAP/TAZ and colocalizes with YAP on chromatin genome-wide. P300 promotes YAP target gene expression primarily through acetylating histones, while YAP protein acetylation is dispensable for YAP transcriptional activity. Finally, mouse xenograft studies demonstrate that p300 inhibition suppress GNAQ-mutant uveal melanoma growth and metastasis in vivo. Overall, this is a solid study uncovering a novel therapeutic target specific for GNAQ-driven uveal melanoma. Methods are sound and conclusions are largely supported by experimental evidence.

A main question I have is the specificity of p300 vs CBP. P300 and CBP are close paralogues that function to compensate each other in most cell lines. In general, p300 becomes an essential gene only when CBP is lost or mutated. It is interesting that in GNAQ-mutant uveal melanoma cells, the authors observed global reduction in H3K27ac upon p300 single knockdown, suggesting that CBP fails to compensate p300 loss in these cells. It would be important to determine, however, the functional difference between p300 and CBP in GNAQ-mutant uveal melanoma. For instance, does CBP also interacts with Yap/TAZ through its KIX domain? What happens to Hippo pathway gene expression and global H3K27ac when CBP is depleted? Since CBP is not essential in GNAQ-mutant uveal melanoma cells, is CBP expressed at much lower levels than p300? And in patients, what is

the CBP mutation status in general in GNAQ-mutant uveal melanoma vs non-mutant UM or BRAF-mutant cutaneous melanoma?

Response:

We thank the reviewer for this important and insightful comment regarding the respective contributions of p300 and CBP in *GNAQ*-mutant uveal melanoma. We agree that this issue deserves clarification, especially because p300 and CBP are closely related paralogous coactivators that often display overlapping functions. In our focused CRISPR/Cas9 synthetic lethality screen, *EP300* ranked first and *CREBBP* ranked eighth among negatively selected genes in *GNAQ*-mutant cells. This result led us to initially focus our mechanistic and *in vivo* studies on p300, while the fact that *CREBBP* also ranked among the top candidates suggested that CBP might also contribute in this setting, prompting the additional experiments and analyses described below.

Regarding whether CBP also interacts with YAP through its KIX domain, we performed co-immunoprecipitation assays and found that CBP, like p300, interacts with YAP through its KIX domain. These data indicate that YAP binding through the KIX domain is not unique to p300 (**Supplementary Fig. 6**). The corresponding result has been added to the revised Results section.

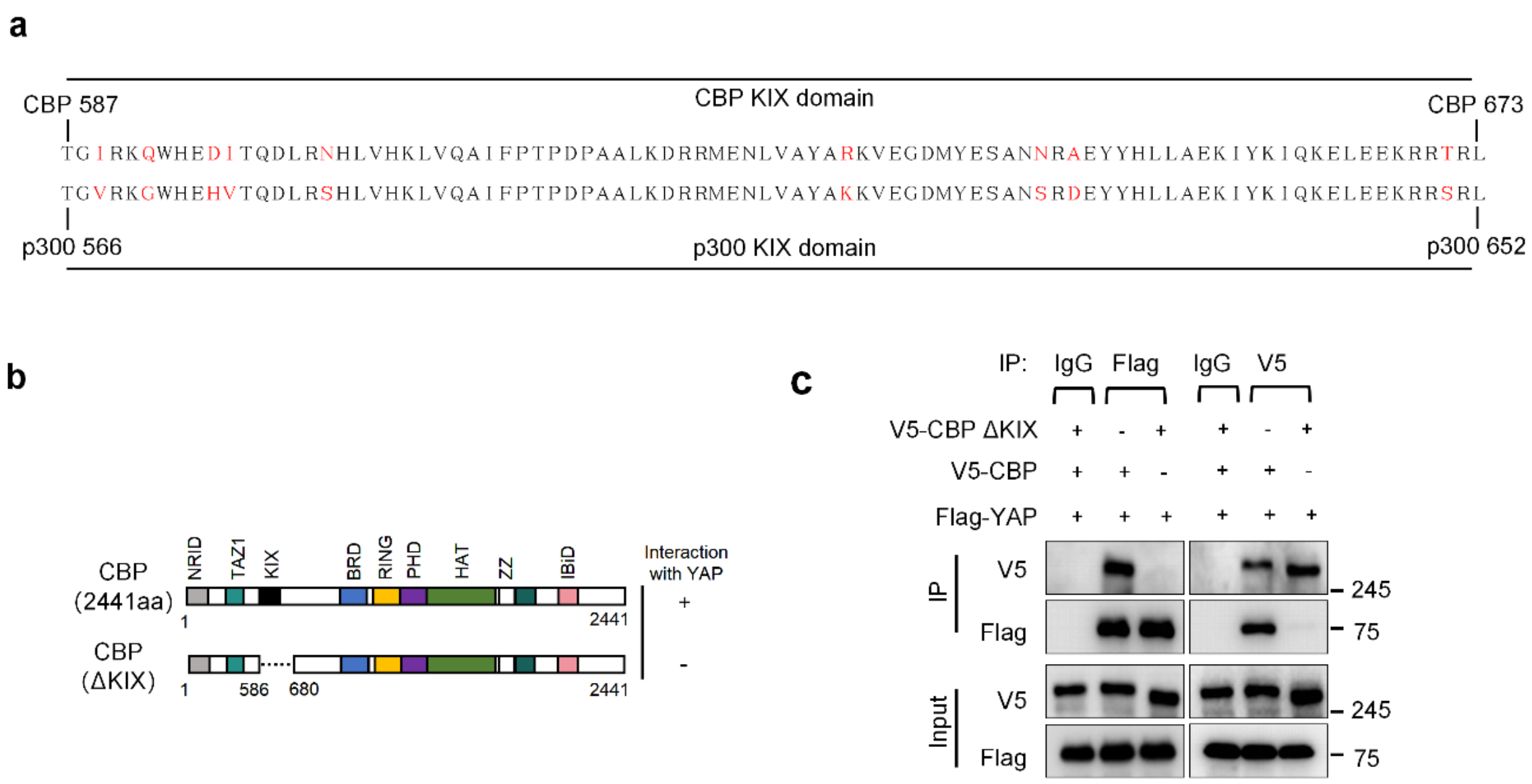

Supplementary Fig. 6: CBP interacts with YAP through its KIX domain.

a, Amino acid sequence alignment of the KIX domains of CBP and p300. **b,** Schematic diagram of the V5-tagged CBP construct used in this study. **c,** The KIX domain of CBP mediates its interaction with YAP. HEK293T cells were transfected with the indicated constructs. YAP or CBP was immunoprecipitated with anti-Flag or anti-V5 antibodies, respectively, and co-immunoprecipitated proteins were detected by

immunoblotting with the indicated antibodies.

Regarding the effect of CBP depletion on YAP-associated gene expression and H3K27ac, we found that CBP knockdown produced broadly similar effects to p300 knockdown in *GNAQ*-mutant UM cells. Specifically, CBP depletion caused a marked reduction in global H3K27ac, as assessed by immunoblotting, and strongly impaired clonogenic growth (**Supplementary Fig. 3a, b**). Furthermore, guided by our YAP ChIP-seq analysis, we selected representative YAP target genes whose promoter regions were occupied by YAP, including *TP73*, *CYR61*, *E2F3*, *DAB2*, and *TGFB1*. ChIP-qPCR validated YAP occupancy at these promoters and showed that CBP knockdown markedly reduced H3K27ac enrichment at these regions (**Supplementary Fig. 12**). In addition, co-depletion of CBP and p300 did not produce a stronger inhibitory effect on clonogenic survival than either single depletion alone, likely because depletion of either protein had already reduced colony formation to a near-minimal level in this assay (**Supplementary Fig. 3c, d**). In 92.1 UM cells, GSEA further showed that CBP knockdown suppressed the YAP-activated gene set used in our original study, indicating that CBP depletion largely recapitulates the effect of p300 depletion at the level of YAP-associated transcriptional output (**Supplementary Fig. 5**). Consistent with this, analysis of the UM TCGA cohort showed that both *EP300* and *CREBBP* mRNA levels positively correlated with the YAP target genes *CYR61* and *CTGF* (**Supplementary Fig. 17**). Together, these results indicate that CBP is also functionally engaged in a YAP-associated transcriptional program in *GNAQ*-mutant UM cells. The corresponding result has been added to the revised Results section.

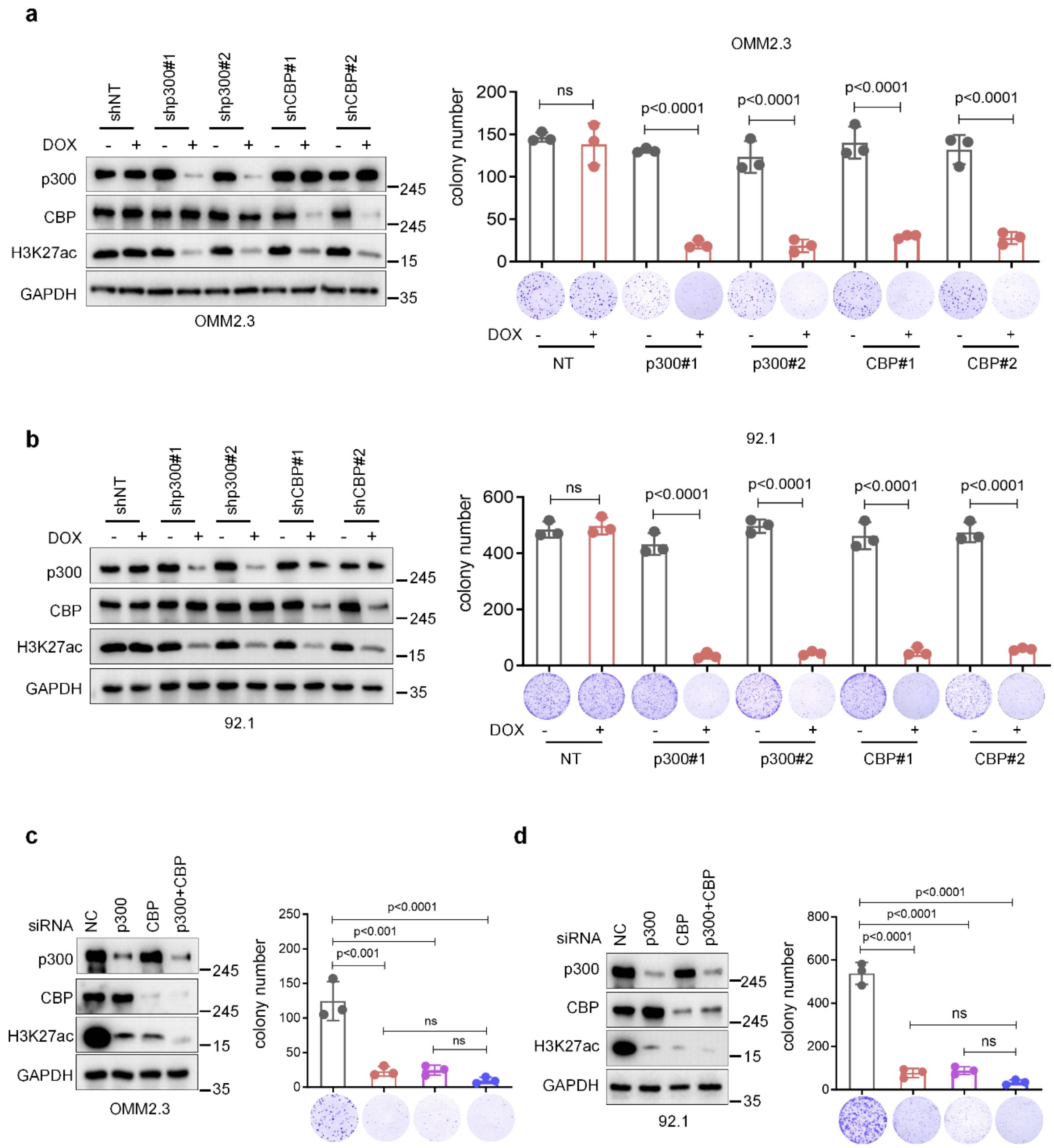

Supplementary Fig. 3: Individual depletion of p300 or CBP suppresses clonogenic growth of Gαq-**mutant UM cells.**

a, b, Immunoblot analysis and colony-formation assays in OMM2.3 (a) and 92.1 (b) cells expressing doxycycline-inducible non-targeting (NT), p300-targeting, or CBP-targeting shRNAs. Cells were treated with doxycycline (200 ng/ml) for 72 h for immunoblot analysis, or for 14 days for colony-formation assays. Data are presented as mean ± SD (n = 3 biological replicates) from one representative experiment out of three. Statistical significance was determined by two-sided Student's t-test. **c, d,** Immunoblot analysis and colony-formation assays in OMM2.3 (c) and 92.1 (d) cells transfected with negative control (NC), p300-targeting, CBP-targeting, or combined p300- and CBP-targeting siRNAs. Knockdown efficiency was assessed by immunoblotting at 48 h after transfection, and cell growth was evaluated by colony-formation assay. Data are presented as mean ± SD (n = 3 biological replicates) from one representative experiment out

of three. Statistical significance was determined by one-way ANOVA with Tukey's multiple comparisons test.

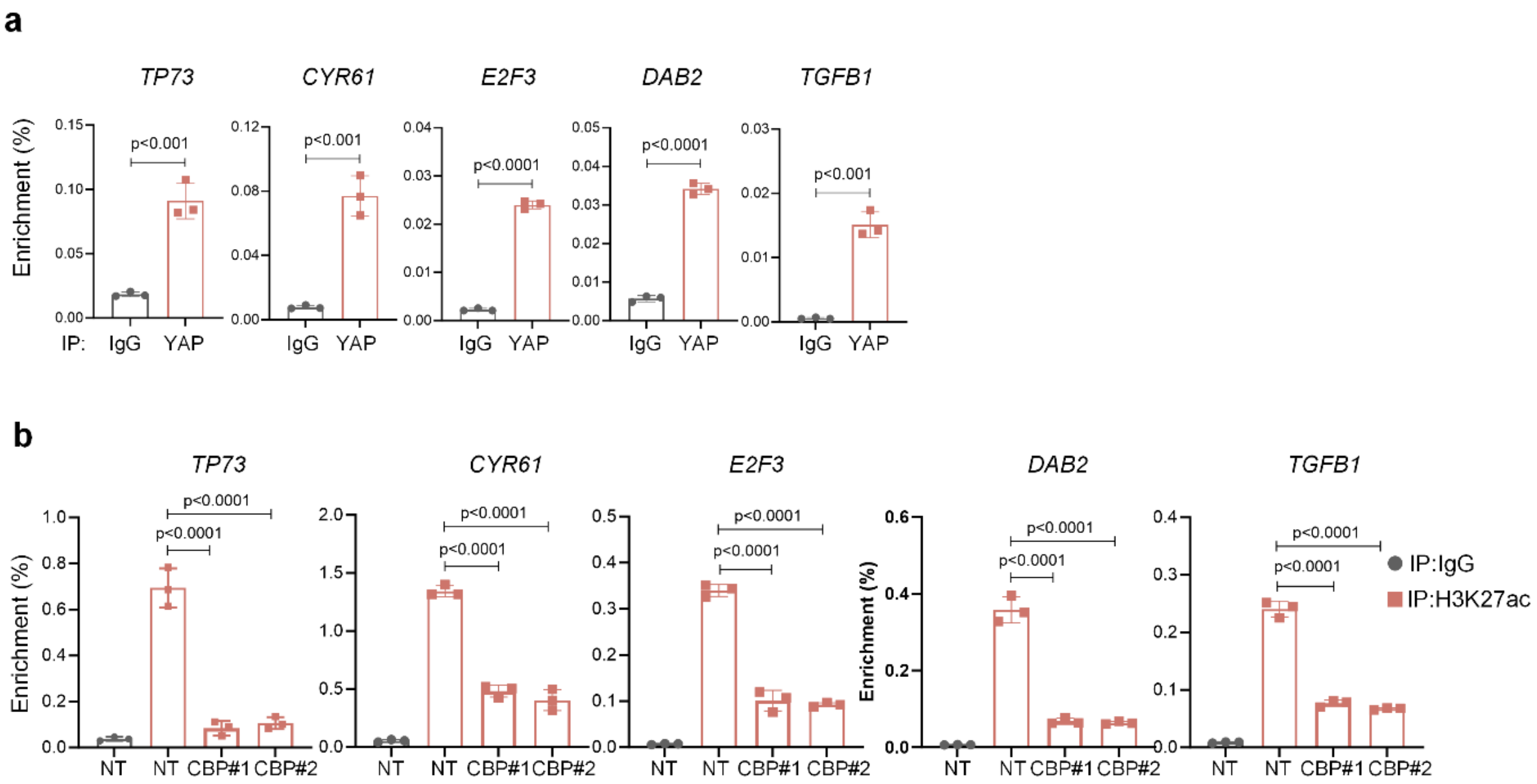

Supplementary Fig. 12: CBP knockdown reduces H3K27ac enrichment at promoters of YAP target genes.

a, ChIP-qPCR analysis showing YAP occupancy at the promoters of selected YAP target genes, including *TP73*, *CYR61*, *E2F3*, *DAB2*, and *TGFB1*. Data are presented as mean ± SD (n = 3 biological replicates) from one representative experiment out of three. Statistical significance was determined by two-sided Student's t-test. **b**, ChIP-qPCR analysis showing that CBP knockdown reduces H3K27ac enrichment at the promoters of YAP target genes, including *TP73*, *CYR61*, *E2F3*, *DAB2*, and *TGFB1*. Data are presented as mean ± SD (n = 3 biological replicates) from one representative experiment out of three. Statistical significance was assessed by one-way ANOVA followed by Tukey's multiple comparisons test.

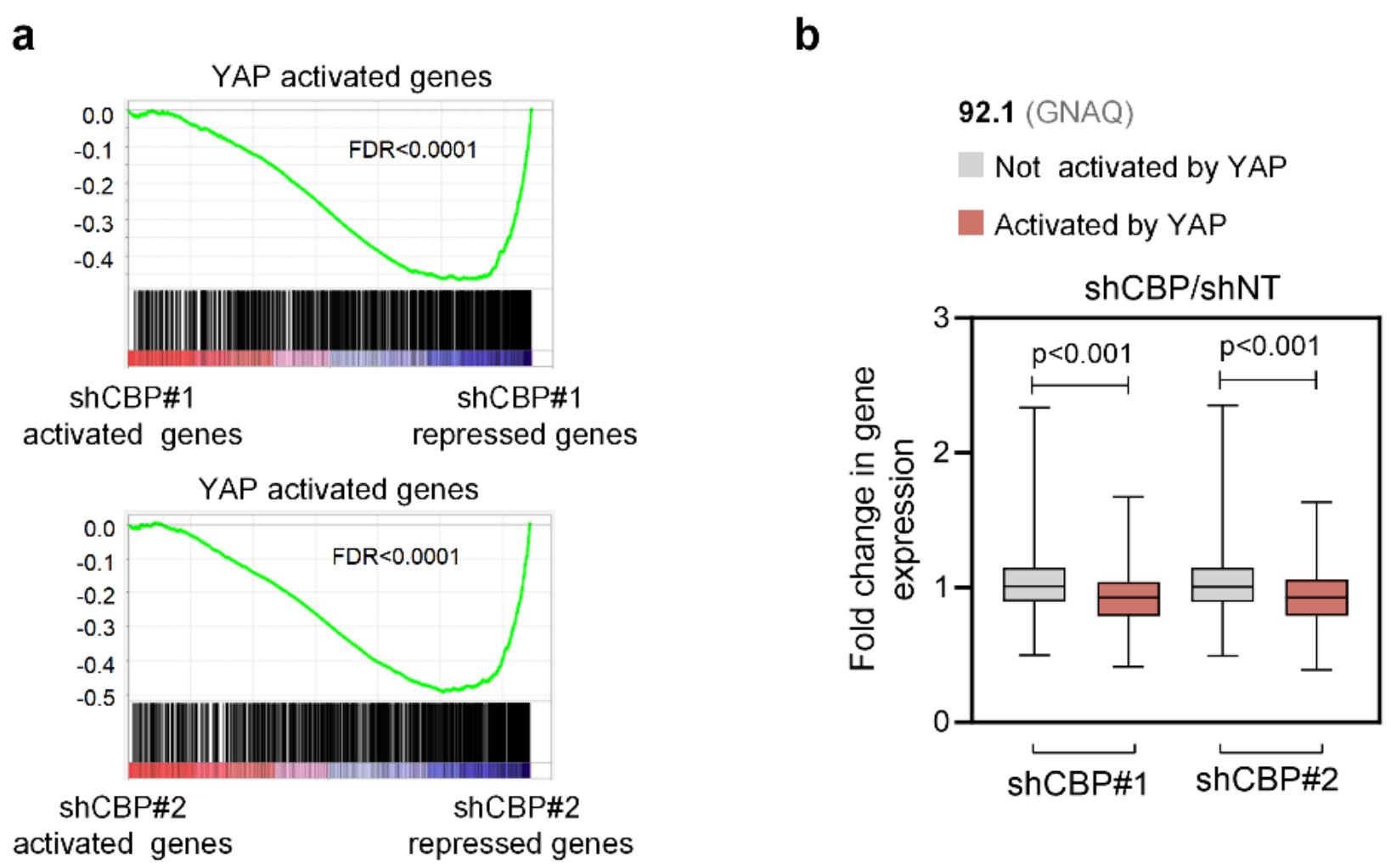

Supplementary Fig. 5 CBP depletion suppresses the YAP-activated transcriptional program.

a, Gene Set Enrichment Analysis (GSEA) showing that most YAP-activated genes were suppressed upon CBP knockdown in 92.1 cells. **b,** Fold change in expression of non-YAP-activated genes (n = 9,339) and YAP-activated genes (n = 1,186) upon CBP knockdown in 92.1 cells, relative to cells transfected with NT shRNA. Boxes indicate the 25th to 75th percentiles, center lines indicate the medians, and whiskers indicate the 2.5th to 97.5th percentiles. Statistical significance was assessed using the Mann-Whitney U-test.

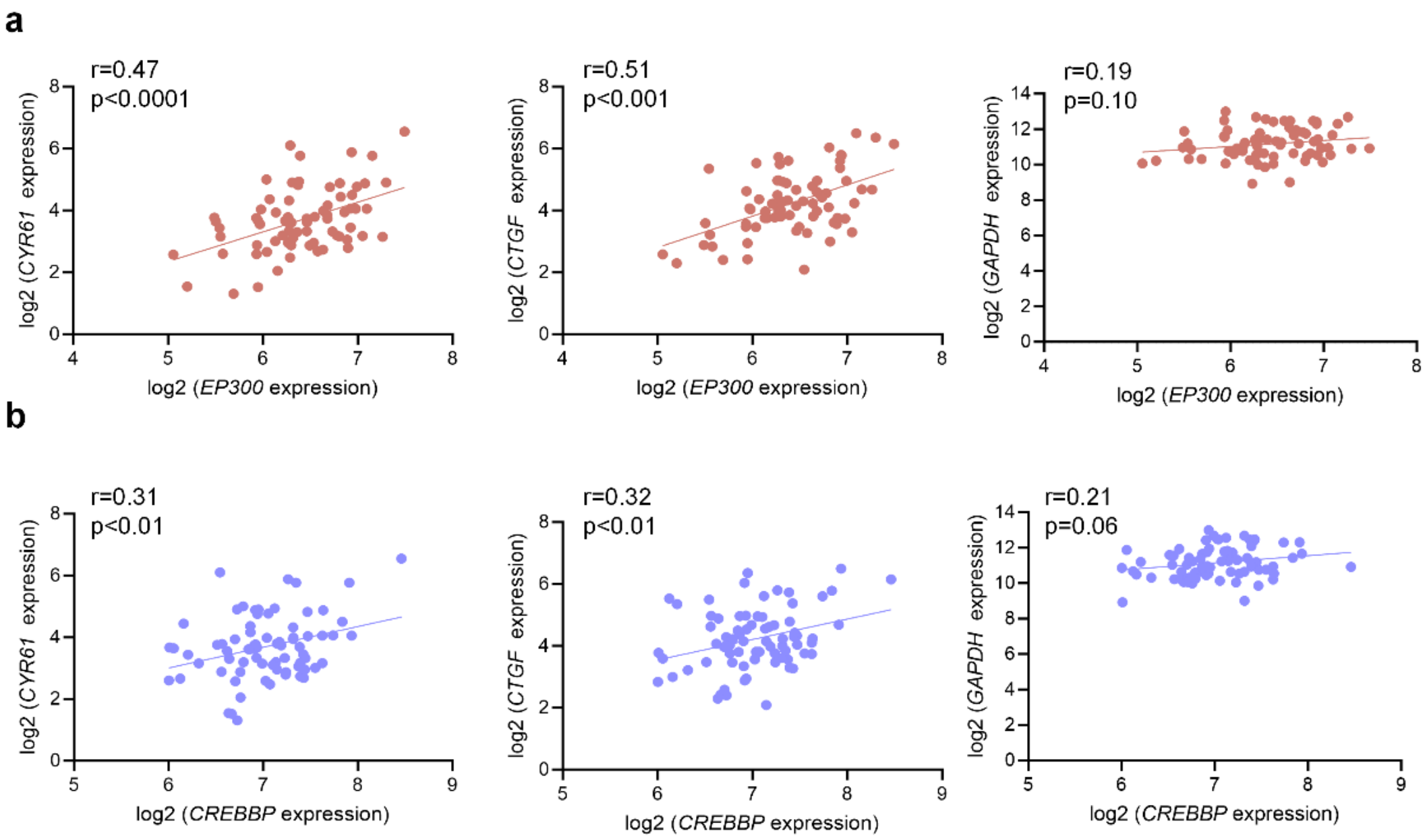

Supplementary Fig. 17: *EP300* and *CREBBP* expression levels correlate with YAP target gene expression in *GNAQ/11*-mutant UM patients.

a and b, *EP300* and *CREBBP* expression levels positively correlate with the expression of the YAP target genes (*CYR61* and *CTGF*) in *GNAQ/11*-mutant uveal melanoma (TCGA, n = 74 patients). *GAPDH* was included as a negative control. Statistical significance was assessed using the Pearson correlation test.

Regarding the possibility that the apparent importance of p300 in *GNAQ*-mutant UM might be due to low CBP expression, analysis of the UM TCGA cohort showed that CREBBP mRNA levels were not lower than *EP300*. Rather, *CREBBP* expression was significantly higher than *EP300* expression in both wild-type and *GNAQ/11*-mutant UM samples (**Supplementary Fig. 16a**). These findings argue against the possibility that the prominent role of p300 in this setting is simply a consequence of reduced *CREBBP* expression.

Regarding *CREBBP* status in patients, analysis of the TCGA UM cohort showed that *CREBBP* mutations were not observed in either *GNAQ/11*-mutant or wild-type UM cases. In contrast, *CREBBP* genetic alterations were present in the TCGA skin cutaneous melanoma

(SKCM) cohort (**Supplementary Fig. 16b**). These findings suggest that the genetic background underlying the previously reported model of p300 dependency in the setting of CBP deficiency (Ogiwara H, 2016) may not be present in *GNAQ/11*-mutant UM.

Taken together, our additional results indicate that, in *GNAQ*-mutant UM, both CBP and p300 are required for maintaining H3K27 acetylation, YAP target gene expression, and clonogenic growth, supporting a model in which both proteins sustain this YAP-associated transcriptional program. We have revised the Discussion accordingly (**Discussion Section, lines 979-1005**).

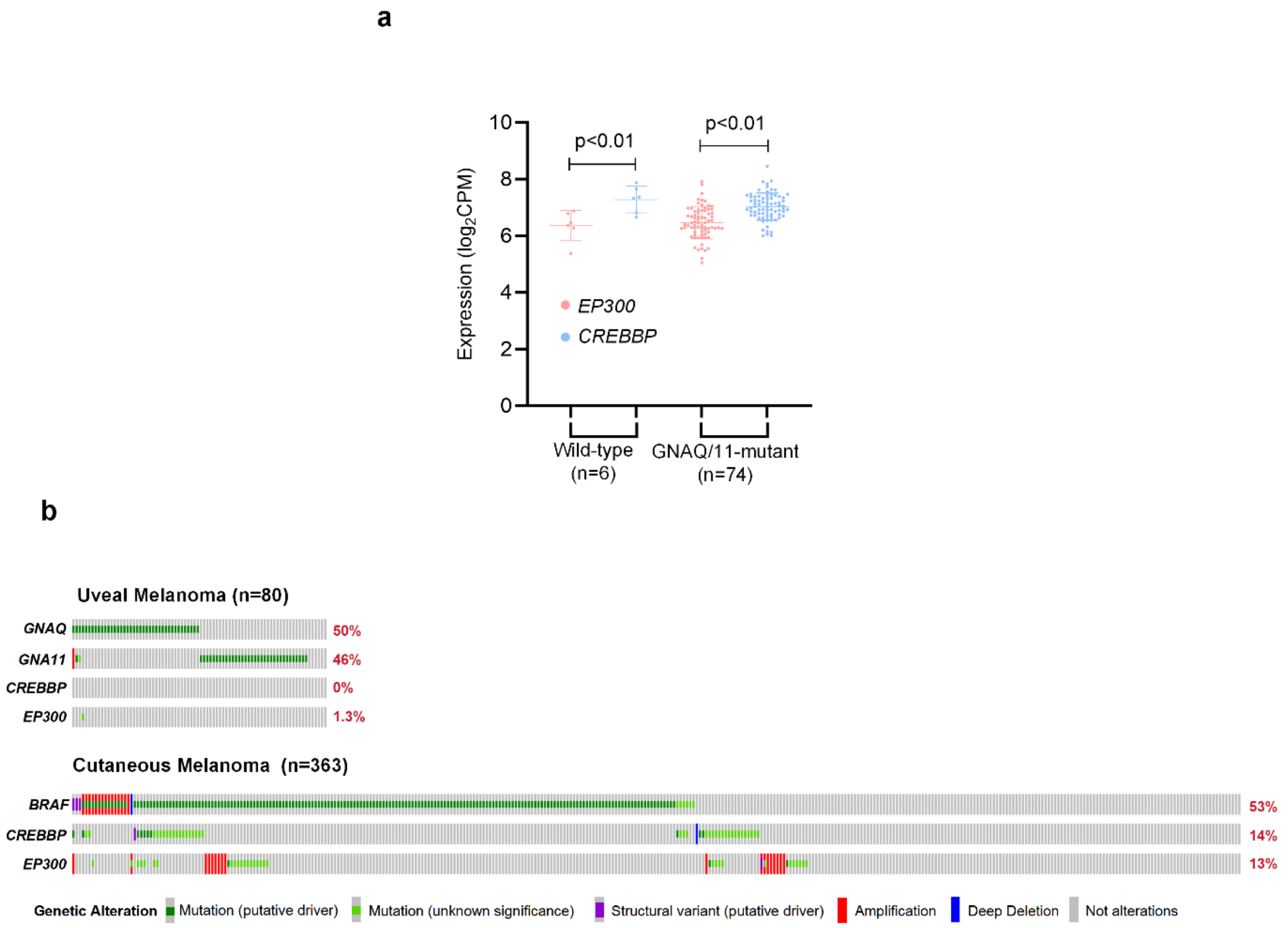

Supplementary Fig. 16: Expression and genomic landscape of *EP300* and *CREBBP* in uveal melanoma and skin cutaneous melanoma.

a, Expression levels of *EP300* and *CREBBP* in TCGA UM cohort (n = 80 patients), based on data from cBioPortal. Normality was assessed using the Shapiro-Wilk test, and normally distributed data were analyzed by two-sided Student's t-test. **b,** Oncoprint of the genomic landscape of *EP300* and *CREBBP* in TCGA UM cohort (n = 80 patients) and skin cutaneous melanoma cohort (n = 363 patients), based on data from cBioPortal.

The revised Discussion section (lines 979–1005) now reads as follows:

"Dependency on p300 and CBP appears to be context-dependent across cancers. In some tumor types, p300 dependency arises in the setting of CBP deficiency, consistent with a paralog addiction model [44, 45]. In other contexts, however, tumor cells may remain dependent on both p300 and CBP [46]. Our findings in Gαq-mutant UM instead support the latter possibility. In UM cells, depletion of either p300 or CBP impaired clonogenic growth, and co-depletion was not more inhibitory than depletion of either paralogue alone. *In vivo*, our study focused on p300, and p300 depletion was sufficient to induce robust anti-growth and anti-metastatic effects. Analysis of the UM TCGA cohort further showed that *CREBBP* expression was not lower than *EP300*, and *CREBBP* mutations were not observed (Supplementary Fig. 16), arguing against a CBP-deficient background in this disease. Consistent with functional involvement of both paralogues, *EP300* and *CREBBP* mRNA levels positively correlated with the YAP target genes *CYR61* and *CTGF* in the UM TCGA cohort (Supplementary Fig. 17). Together, these observations suggest that dependency on p300 or CBP in Gαq-mutant UM is unlikely to be explained by loss of the other paralogue. Evidence from knockout studies in the mouse has shown that CBP and p300 are critical for the development and function of normal cells and tissues [47, 48]. Therefore, inhibition of either p300 or CBP alone may be sufficient to exert anti-tumor effects and could still offer an expanded therapeutic index in Gαq-mutant UM. Because *EP300* emerged as the top-ranked hit in our screen and p300 was the paralogue validated most extensively *in vivo* in this study, p300-directed strategies provide a practical example for therapeutic development. While current HAT and BRD small molecule inhibitors cannot discriminate between CBP and p300, recently developed small-molecule and peptide proteolysis-targeting chimera (PROTAC) drugs demonstrate potent and selective p300 degradation [49, 50]. This highlights the potential of p300-targeting PROTACs as a promising therapeutic approach. Additionally, we propose that new therapeutics may be designed around the YAP-p300 interaction, particularly by targeting the p300 KIX domain. Interestingly, there has been growing interest in investigating KIX domain inhibitors, and several small molecule KIX inhibitors have been reported [51, 52, 53]."

Finally, although our data do not define the precise architecture of the relevant transcriptional complexes, they support a cautious working model. Prior studies suggest that YAP and TAZ can form homo- and heterodimers (**Murakami M, 2005; Khanal P, 2018; Ben C, 2020**). Structural and biochemical studies have also shown that transcription factor dimerization can activate p300 by promoting trans-autoacetylation of its autoinhibitory loop, thereby relieving

autoinhibition and stimulating its acetyltransferase activity (**Ortega E, 2018**). Given the close paralogy and shared regulatory features of p300 and CBP, a similar mechanism may also apply to CBP. Furthermore, prior work has shown that a transcription factor can co-occupy genomic loci with both CBP and p300, accompanied by local regulation of H3K27ac (**Li S, 2018**). In light of these studies, together with our data showing that both CBP and p300 interact with YAP and are required for YAP-associated transcriptional output in UM cells, we propose that YAP-associated transcriptional complexes in *GNAQ*-mutant UM may engage both CBP and p300 to support YAP target gene expression. The precise composition and organization of such complexes, however, remain to be determined. We have revised the Discussion accordingly (**Discussion Section, lines 942-978**).

The revised Discussion section (lines 942–978) now reads as follows:

"Although p300/CBP have been proposed as general regulators of RNA polymerase II-dependent transcription, genome-wide studies have instead shown that p300/CBP inhibition does not globally or indiscriminately suppress cellular transcription but rather more selectively impedes transcription of distinct oncogenic networks across different cancer types[33]. In line with these findings, we demonstrated that p300/CBP inhibition selectively disrupts the oncogenic YAP transcriptional network, which may contribute to the marked sensitivity of Gαq-mutant UM to p300/CBP inhibition. This hypersensitivity likely arises because Gαq-mutant UM is highly dependent on YAP for survival and growth [13, 14, 39]. Additionally, this sensitivity may also reflect activation of p300/CBP HAT activity by YAP-associated complexes assembled on chromatin. It has been reported that not all p300/CBP recruitment events result in chromatin acetylation and gene expression, indicating that HAT activity at certain sites may be restrained [40, 41]. Indeed, p300/CBP exist in two states: basal and active. In the basal state, the auto-inhibitory loop (AIL) occupies the substrate-binding groove and interacts with the RING domain, thereby blocking the active site of the enzyme. In the active state, the AIL is autoacetylated and disengages from the active site, allowing the RING domain to shift position and the enzyme to become active [26]. Interestingly, transcription factor dimerization has been shown to activate p300 HAT [42]. Specifically, when transcription factors dimerize, they adopt a DNA-binding-competent conformation, enabling them to engage two p300 molecules in the nucleus. This interaction increases the likelihood of AIL disengagement from its inhibitory position in cis and its capture in trans by a second p300 molecule [42]. Notably, YAP can form stable homodimers, which exhibit enhanced

oncogenic behavior compared to their monomeric forms [43]. In light of these studies, together with our data showing that both CBP and p300 are required for YAP-associated transcriptional output in UM cells, we propose that YAP dimerization may promote the formation of YAP-associated transcriptional complexes that engage both p300 and CBP and facilitate HAT activation within these complexes to support YAP target gene expression in Gαq-mutant UM. The marked reduction of global H3K27ac after depletion of either paralogue, together with reduced H3K27ac at YAP target promoters, raises the possibility that YAP-associated p300/CBP complexes regulate H3K27ac as part of a dynamic acetylation–deacetylation process rather than through a simple static deposition mechanism. In this context, endogenous HDAC activity may also influence the turnover and homeostasis of H3K27ac at YAP target loci, adding another layer of complexity to YAP-dependent transcriptional regulation in Gαq-mutant UM. The precise composition, organization and functional regulation of these YAP-associated p300/CBP complexes, however, remain to be determined. This mechanism may help sustain and reinforce YAP transcriptional activity in Gαq-mutant UM, and the functional interdependency between YAP and p300/CBP may help explain the marked sensitivity of Gαq-mutant UM to p300/CBP inhibition."

References:

Ogiwara H, et al. Targeting p300 addiction in CBP-deficient cancers causes synthetic lethality by apoptotic cell death due to abrogation of MYC expression. Cancer Discov, 2016, 6(4):430-45.

Murakami M, et al. A WW domain protein TAZ is a critical coactivator for TBX5, a transcription factor implicated in Holt-Oram syndrome. Proc Natl Acad Sci USA, 2005, 102(50):18034-9.

Khanal P, et al. Cysteine residues are essential for dimerization of Hippo pathway components YAP2L and TAZ. Sci Rep, 2018, 8(1):3485.

Ben C, et al. Alternative splicing reverses the cell-intrinsic and cell-extrinsic pro-oncogenic potentials of YAP1. J Biol Chem, 2020, 295(41):13965-80.

Ortega E, et al. Transcription factor dimerization activates the p300 acetyltransferase. Nature, 2018, 562(7728):538-44.

Li S, et al. DYRK1A interacts with histone acetyl transferase p300 and CBP and localizes to enhancers. Nucleic Acids Res, 2018, 46(21):11202-13.

Minor points:

• Graphic abstract: "GNAQ/YAP NO" should be "GNAQ/YAP ON".

Response:

We thank the reviewer for noting this error. It has been corrected in the graphic abstract.

• Line 391 – "Fig. 2a-b" should be "Supplementary Fig. 2a-b"?

Response:

We thank the reviewer for catching this mistake. We have corrected the citation to "Supplementary Fig. 2a-b" in the revised manuscript.

• Fig. 5, it seems YAP ChIP-seq was conducted in shp300 cells, it would be helpful to show it in heatmap as Fig. 5d and 5e.

Response:

We thank the reviewer for this helpful suggestion. The YAP ChIP-seq heatmap in shp300 cells has now been incorporated into Fig. 5d.

Figure 5d

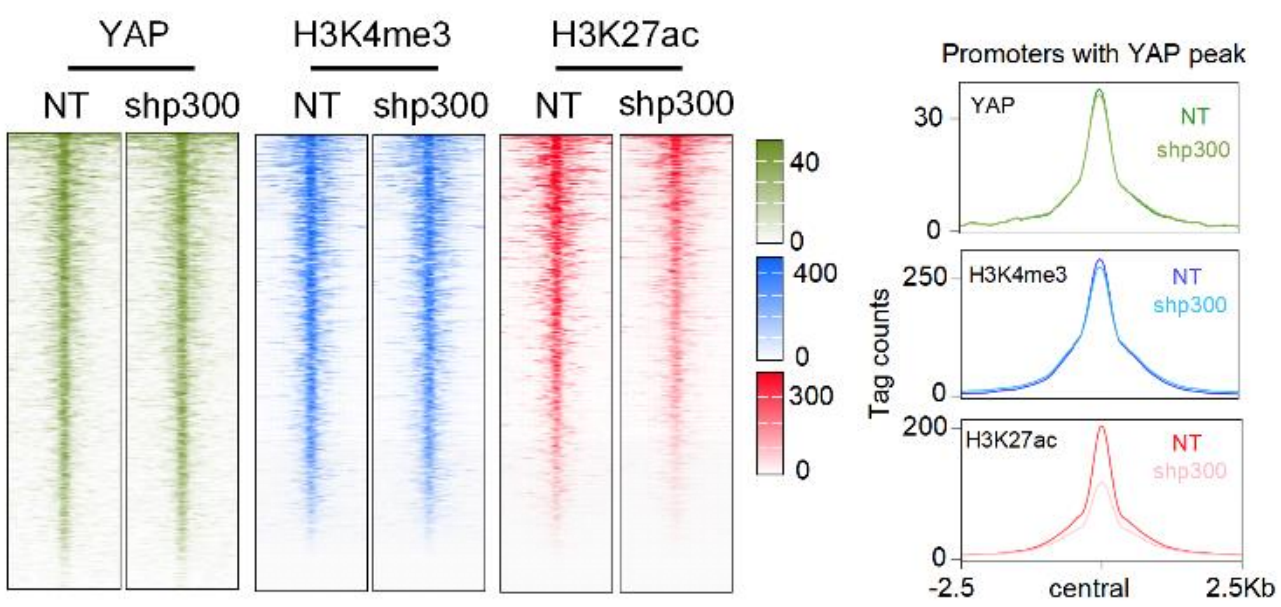

Figure 5d, Heatmaps (left) and average line plots (right) showing YAP, H3K4me3, and H3K27ac ChIP-seq signals at promoters bound by YAP in 92.1 cells, with or without p300 depletion.

Reviewer #4 (Remarks to the Author):

I co-reviewed this manuscript with one of the reviewers who provided the listed reports. This is part of the Nature Communications initiative to facilitate training in peer review and to provide appropriate recognition for Early Career Researchers who co-review manuscripts.

Response:

We thank the reviewer for the careful evaluation of our manuscript.

Point-by-point response to the reviewers' comments:

REVIEWER COMMENTS

Reviewer #1 (Remarks to the Author):

The authors have robustly responded to all my concerns. The authors are to be commended for such an extensive and conscientious rebuttal to all reviewer comments.

Response:

We sincerely thank the reviewer for the positive evaluation of our revised manuscript and for recognizing our efforts to address the previous concerns. We greatly appreciate the reviewer's constructive comments, which helped us improve the clarity, rigor, and overall presentation of the study.

Reviewer #2 (Remarks to the Author):

While the authors have made some improvements, several important issues raised in the previous round have not been adequately addressed, and new concerns have emerged. I therefore recommend further revision before the paper can be considered for publication in Nature Communications.

My specific comments are as follows:

1. The authors highlight in the introduction that direct Gαq inhibitors (e.g., FR900359, YM-254890) cannot differentiate between wild-type and mutant Gαq, leading to unacceptable toxicity. This raises the question: how might BET or p300 inhibitors be employed to selectively target Gαq-mutant uveal melanoma cells over wild-type Gαq-expressing cells, thus distinguishing efficacy from toxicity?

Response:

We thank the reviewer for raising this important point. We agree that BET or p300/CBP inhibitors are not expected to distinguish mutant from wild-type Gαq through direct target binding. Rather, their potential selectivity would be expected to arise from a context-dependent transcriptional dependency created by oncogenic Gαq signaling. We have revised

the Discussion to clarify this point. The revised Discussion now reads as follows (**lines 955-963** in the revised manuscript):

"The potential selectivity of targeting p300/CBP is unlikely to arise from direct discrimination between mutant and wild-type Gαq. Rather, it may reflect a context-dependent transcriptional dependency created by oncogenic Gαq signaling. In our study, this interpretation is supported by the selective depletion of *EP300* and *CREBBP* in the isogenic Gαq(Q209P) screen, the preferential sensitivity of Gαq-mutant UM cells to p300/CBP inhibition compared with Gαq-wild-type or *BRAF*-mutant melanoma cells, and the antitumor activity of p300 inhibition *in vivo* at tolerated doses. Conceptually, this is consistent with previous studies showing that BET inhibition can preferentially suppress YAP/TAZ-dependent transcriptional addiction through BRD4 [27]."

2. "However, accumulating evidence indicates that these downstream branches do not contribute equally to UM maintenance." The meaning of this statement is unclear, and it lacks a supporting reference.

Response:

We thank the reviewer for pointing out this unclear statement. We agree that the original wording was too general and lacked sufficient context. We have revised this sentence to specify the downstream branches being discussed, to more clearly reflect the supporting evidence, and to include the corresponding references. The revised text now reads as follows (**lines 75-79** in the revised manuscript):

"Although oncogenic Gαq activates multiple downstream pathways, available evidence points to YAP signaling as a particularly important effector in Gαq-driven UM [12, 13, 14, 15]. In this context, MEK inhibition has shown limited clinical benefit in metastatic UM [12], whereas YAP has been demonstrated to mediate key tumorigenic outputs of mutant Gαq/11 in experimental UM models [13, 14, 15]."

3. Supplementary Fig. 7a should include an immunoblot for anti-panTEAD on the IP samples. Furthermore, although p300 can directly interact with YAP, it remains unclear why this interaction would be important than other YAP independent function of p300. I believe domain-specific functional assays should be involved to address this.

Response:

We thank the reviewer for these helpful suggestions. First, we have now added an anti-panTEAD immunoblot for the IP samples in Supplementary Fig. 7a, using the same IP samples analyzed in the original experiment. This confirms TEAD depletion in the relevant samples and further supports that the YAP-p300 interaction detected in this assay is not dependent on TEAD.

Second, regarding the functional importance of the YAP-p300 interaction, we agree that domain-specific functional assays are important to distinguish the contribution of the YAP-binding region from other functional domains of p300. We did not intend to imply that the KIX-mediated interaction with YAP is more important than other YAP-independent functions of p300. Rather, our model is that p300 acts as a multi-domain coactivator whose full activity in YAP-dependent gene expression appears to require multiple functional domains.

To address this point, we have added new domain-deletion functional assays in Supplementary Fig. 8. We focused on the KIX, BRD, and HAT domains because the KIX domain mediates YAP binding in our mapping experiments, whereas the BRD and HAT domains are the pharmacologically targeted domains examined in our inhibitor studies. These experiments show that deletion of the KIX, bromodomain, or HAT domain impairs the ability of p300 to promote the expression of the YAP target genes *CTGF* and *CYR61*. The reduced activity of the ΔKIX mutant supports the functional relevance of the YAP-p300 interaction itself, whereas the reduced activity of the ΔBRD and ΔHAT mutants indicates that this interaction cooperates with other functional domains of p300 to support YAP-dependent gene expression. Together, these findings support a model in which the KIX-mediated YAP-p300 interaction is functionally important and works together with other p300 domains to promote YAP-dependent gene expression. We have incorporated these new data into the revised manuscript as follows (**lines 644-651** in the revised manuscript):

"To further define the p300 domains required for YAP-dependent gene expression, we generated p300 mutants lacking the KIX domain, bromodomain (BRD), or HAT domain and co-expressed them with YAP in HEK293T cells (Supplementary Fig. 8a, b). RT-qPCR analysis showed that deletion of any of these domains markedly impaired the ability of p300 to induce the expression of the YAP target genes *CTGF* and *CYR61* (Supplementary Fig. 8c). These results indicate that the KIX-mediated p300-YAP interaction, together with the

functional contributions of the BRD and HAT domains, contributes to p300-dependent regulation of YAP target gene expression."

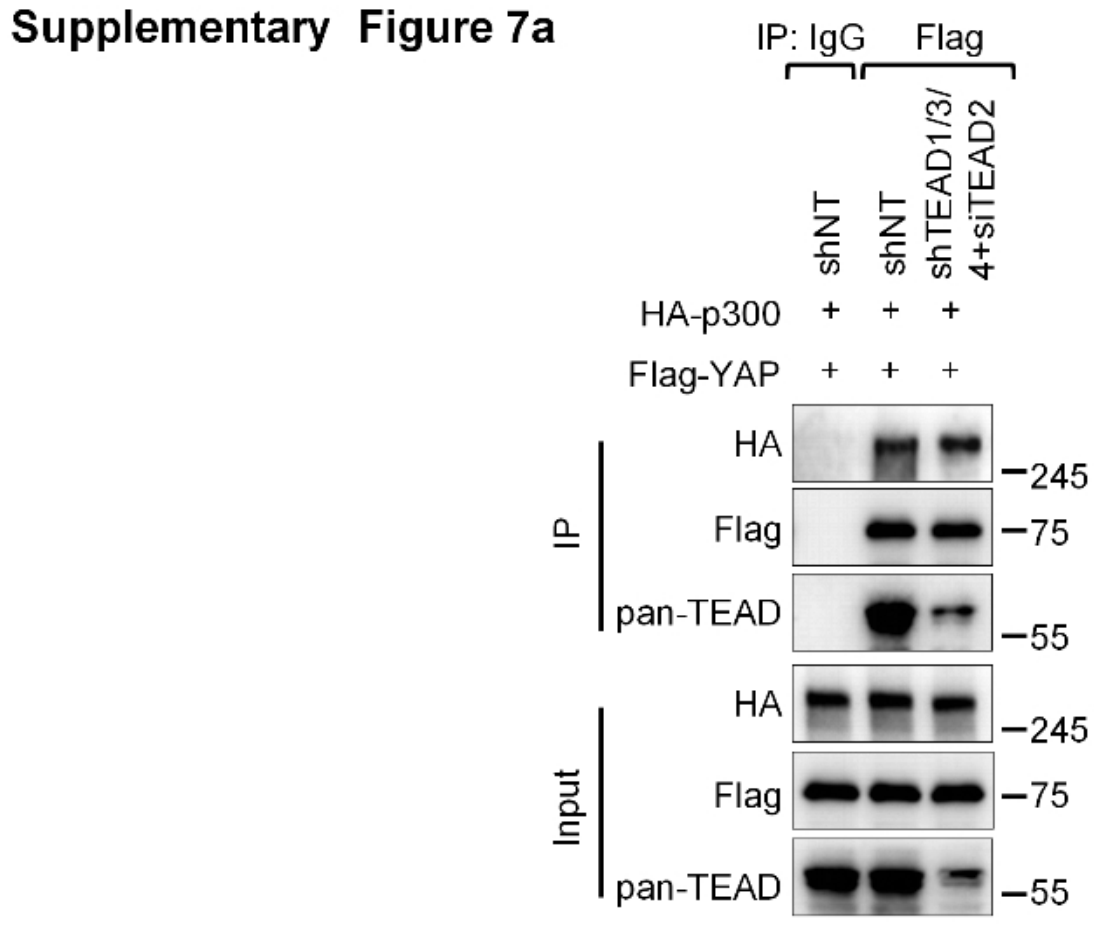

Supplementary Fig. 7: YAP directly binds p300 in a TEAD-independent manner.

a, TEAD knockdown does not impair the interaction between YAP and p300. HEK293T cells stably expressing shRNAs targeting TEAD1, 3, and 4 were transfected with TEAD2 siRNA together with YAP and p300 constructs. Cells were harvested 48 h after transfection and subjected to co-immunoprecipitation followed by immunoblotting with the indicated antibodies. Cells expressing non-targeting shRNA (shNT) served as controls.

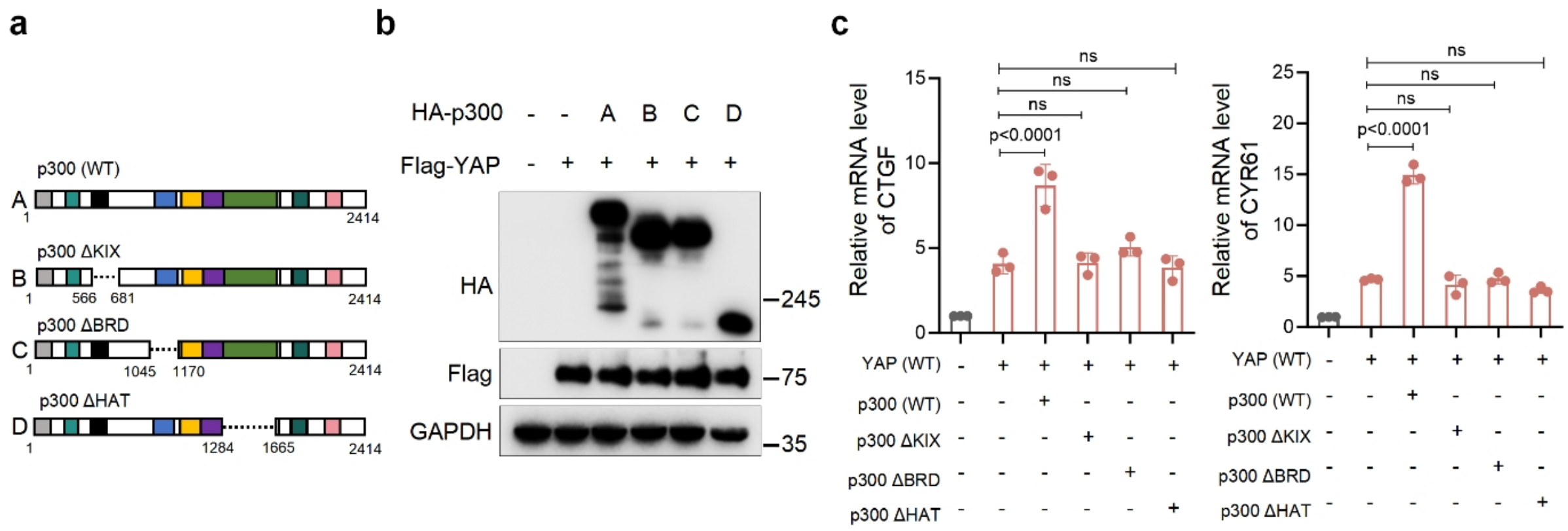

Supplementary Fig. 8: Deletion of the KIX, BRD, or HAT domain impairs p300-mediated induction of YAP target gene expression.

a, Schematic representation of the HA-tagged wild-type and domain-deletion p300 constructs used in this study.

b and c, Deletion of the KIX, BRD, or HAT domain impairs the ability of p300 to promote the expression of YAP target genes. HEK293T cells were transfected with the indicated constructs for 48 h. Expression of the p300 constructs was confirmed by immunoblotting (**b**), and mRNA levels of the YAP target genes *CTGF* and *CYR61* were measured by qPCR (**c**). Data are presented as the mean ± SD (n = 3) from one

representative experiment out of two. Statistical significance was determined by one-way ANOVA followed by Tukey's multiple-comparison test.

4. A previous review comment asked the authors to "explain how p300-mediated histone modifications influence plasmid reporter activity." I find that the authors neither adequately addressed nor correctly understood this comment. According to their proposed mechanism, p300 promotes YAP transcriptional activity via H3K27ac-dependent epigenetic modifications (Supplementary Fig. 4g). However, a transfected CTGF promoter-luciferase plasmid lacks histone-organized genomic DNA and should be insensitive to epigenetic regulation. Nonetheless, p300 knockdown reduced this reporter activity. The authors need to explain this discrepancy and clarify how their data support the original comment.

Response:

We thank the reviewer for raising this important point and for giving us the opportunity to clarify the interpretation of the *CTGF* promoter-luciferase assay. We agree that a transiently transfected *CTGF* promoter-luciferase reporter does not fully recapitulate the chromatin organization of the endogenous *CTGF* locus. At the same time, prior studies suggest that transiently transfected plasmids are not necessarily maintained as naked DNA, but can acquire episomal chromatin-like organization [**1-3**]. Studies using defined chromatinized templates have further shown that p300/CBP can promote transcription through their HAT/coactivator functions [**4-6**]. Consistent with this concept, prior studies using transient luciferase promoter-reporter assays similar to the system used in our study, including pGL3-based systems [**7**], have shown that p300/CBP overexpression, depletion, or functional inhibition can modulate promoter-reporter activity [**7-9**]. Thus, we interpret Fig. 2g as a promoter-reporter functional assay showing that p300 depletion reduces *CTGF* promoter-driven reporter activity, rather than as direct evidence by itself for H3K27ac-dependent regulation at the endogenous *CTGF* locus.

To complement this reporter-based analysis, we examined H3K27ac enrichment at the endogenous *CTGF* promoter in OMM2.3 cells and found that p300 depletion reduced local H3K27ac enrichment at the TEAD-binding region of the endogenous *CTGF* promoter (Supplementary Fig. 4d). Moreover, our proposed chromatin-based mechanism is supported by multiple endogenous-locus and genome-wide analyses, including RNA-seq, ChIP-seq for YAP, p300, H3K27ac, RNA polymerase II and CDK9, and ChIP-qPCR validation at YAP target loci. We have revised the manuscript text to clarify the interpretation of the reporter

assay and its relationship to the endogenous chromatin data. The revised text now reads (**lines 555-564** in the revised manuscript):

"We next focused on *CTGF*, a representative YAP target gene, to further examine the effect of p300 depletion on YAP target gene regulation. In OMM2.3 cells, p300 knockdown significantly reduced *CTGF* promoter–luciferase reporter activity (Fig. 2g). Because a transiently transfected promoter-luciferase reporter does not fully recapitulate the chromatin organization of the endogenous *CTGF* locus, we further examined H3K27ac enrichment at the endogenous *CTGF* promoter in the same cellular context. H3K27ac ChIP-qPCR at the endogenous *CTGF* promoter region spanning the TEAD-binding site showed that p300 depletion reduced local H3K27ac enrichment (Supplementary Fig. 4d). Together, these results indicate that p300 supports *CTGF* promoter-driven reporter activity and is also required for H3K27ac enrichment at the endogenous *CTGF* promoter in OMM2.3 cells."

References:

[1] Cereghini S, Yaniv M. Assembly of transfected DNA into chromatin: structural changes in the origin-promoter-enhancer region upon replication. EMBO J. 1984;3(6):1243-1253.
[2] Reeves R, Gorman CM, Howard B. Minichromosome assembly of non-integrated plasmid DNA transfected into mammalian cells. Nucleic Acids Res. 1985;13(10):3599-3615.
[3] Mallory BJ, Tullius TW, Biar CG, Gustafson JA, Bohaczuk SC, Dubocanin D, Starita LM, Stergachis AB. Principles and functional consequences of plasmid chromatinization in mammalian cells. bioRxiv. 2025.
[4] Lu H, Pise-Masison CA, Fletcher TM, Schiltz RL, Nagaich AK, Radonovich M, Hager G, Cole PA, Brady JN. Acetylation of nucleosomal histones by p300 facilitates transcription from tax-responsive human T-cell leukemia virus type 1 chromatin template. Mol Cell Biol. 2002;22(13):4450-4462.
[5] Hardy S, Brand M, Mittler G, Yanagisawa J, Kato S, Meisterernst M, Tora L. TATA-binding protein-free TAF-containing complex (TFTC) and p300 are both required for efficient transcriptional activation. J Biol Chem. 2002;277(36):32875-32882.
[6] Huang ZQ, Li J, Sachs LM, Cole PA, Wong J. A role for cofactor-cofactor and cofactor-histone interactions in targeting p300, SWI/SNF and Mediator for transcription. EMBO J. 2003;22(9):2146-2155.
[7] Etchegaray JP, Lee C, Wade PA, Reppert SM. Rhythmic histone acetylation underlies transcription in the mammalian circadian clock. Nature. 2003;421(6919):177-182.

[8] Zhai W, Ye X, Wang Y, Feng Y, Wang Y, Lin Y, Ding L, Yang L, Wang X, Kuang Y, Fu X, Eugene Chin Y, Jia B, Zhu B, Ren F, Chang Z. CREPT/RPRD1B promotes tumorigenesis through STAT3-driven gene transcription in a p300-dependent manner. Br J Cancer. 2021;124(8):1437-1448.
[9] Zhong Z, Harmston N, Wood KC, Madan B, Virshup DM. A p300/GATA6 axis determines differentiation and Wnt dependency in pancreatic cancer models. J Clin Invest. 2022;132(12).

Given the above concerns, I believe the manuscript requires substantial additional experimentation and/or rewriting to clarify these points. I look forward to seeing a revised version that addresses these issues.

Response:

We thank the reviewer for the constructive assessment and helpful suggestions. We agree that these points required further clarification, and we have made substantial revisions to the manuscript accordingly. In the revised manuscript, we have added new experimental data, including the anti-panTEAD immunoblot in Supplementary Fig. 7a and the domain-deletion functional assays in Supplementary Fig. 8, and have revised the relevant text to clarify the interpretation and mechanistic implications of these results. We believe that these additions and revisions have strengthened the manuscript and addressed the reviewer's concerns.

Reviewer #3 (Remarks to the Author):

The authors have adequately addressed my questions. However, given the added results, the title should be changed to "Synthetic lethal screens reveal p300/CBP as a targetable vulnerability for GNAQ-driven uveal melanoma" to better reflect the findings.

Response:

We thank the reviewer for this helpful suggestion. We agree that, in light of the additional CBP data included in the revised manuscript, the title should better reflect the involvement of both p300 and CBP. We have therefore revised the title to "Synthetic lethal screens reveal p300/CBP as targetable vulnerabilities for *GNAQ*-driven uveal melanoma." We have also made corresponding minor revisions to the Abstract to maintain consistency with the revised title.

Reviewer #4 (Remarks to the Author):

I co-reviewed this manuscript with one of the reviewers who provided the listed reports. This is part of the Nature Communications initiative to facilitate training in peer review and to provide appropriate recognition for Early Career Researchers who co-review manuscripts.

Response:

We thank the reviewer for their careful evaluation of our manuscript and for their contribution to the peer-review process.

Point-by-point response to the reviewers' comments:

REVIEWER COMMENTS

Reviewer #2 (Remarks to the Author):

The authors have adequately addressed all of my previous comments and concerns, and I have no further issues to raise.

Response:

We thank the reviewer for confirming that all previous comments and concerns have been adequately addressed and for the constructive feedback that helped us improve the manuscript.
